# Quantifying the regime of thermodynamic control for solid-state reactions during ternary metal oxide synthesis

**DOI:** 10.1126/sciadv.adp3309

**Published:** 2024-07-03

**Authors:** Nathan J. Szymanski, Young-Woon Byeon, Yingzhi Sun, Yan Zeng, Jianming Bai, Martin Kunz, Dong-Min Kim, Brett A. Helms, Christopher J. Bartel, Haegyeom Kim, Gerbrand Ceder

**Affiliations:** ^1^ Department of Materials Science and Engineering, UC Berkeley, Berkeley, CA 94720, USA.; ^2^ Materials Sciences Division, Lawrence Berkeley National Laboratory, Berkeley, CA 94720, USA.; ^3^ Energy and Photon Sciences Directorate, Brookhaven National Laboratory, Upton, NY 11973, USA.; ^4^ The Advanced Light Source, Lawrence Berkeley National Laboratory, Berkeley, CA 94720, USA.; ^5^ Molecular Foundry, Lawrence Berkeley National Laboratory, Berkeley, CA 94720, USA.; ^6^ Department of Chemical Engineering and Materials Science, University of Minnesota, Minneapolis, MN 55455, USA.

## Abstract

The success of solid-state synthesis often hinges on the first intermediate phase that forms, which determines the remaining driving force to produce the desired target material. Recent work suggests that when reaction energies are large, thermodynamics primarily dictates the initial product formed, regardless of reactant stoichiometry. Here, we validate this principle and quantify its constraints by performing in situ characterization on 37 pairs of reactants. These experiments reveal a threshold for thermodynamic control in solid-state reactions, whereby initial product formation can be predicted when its driving force exceeds that of all other competing phases by ≥60 milli–electron volt per atom. In contrast, when multiple phases have a comparable driving force to form, the initial product is more often determined by kinetic factors. Analysis of the Materials Project data shows that 15% of possible reactions fall within the regime of thermodynamic control, highlighting the opportunity to predict synthesis pathways from first principles.

## Teaser

In situ characterization reveals the conditions where reaction outcomes can be predicted using thermodynamic calculations.

## INTRODUCTION

Solid-state reactions are a cornerstone of modern inorganic chemistry, underpinning the synthesis and processing of countless materials (*1*–*3*). Despite their prevalence, it remains difficult to predict the outcomes of solid-state reactions, which typically proceed through a series of intermediate phases whose formation is governed by a combination of thermodynamic and kinetic factors (*4*–*10*). The pathway taken by a solid-state reaction is often set by the initial phase that forms, as it consumes much of the free energy associated with the starting materials (*11*). Understanding which product will emerge from specific precursors can greatly improve synthesis planning, allowing researchers to design reaction pathways that maintain a large driving force to produce their intended target (*12*, *13*). In this work, we advance predictive synthesis with the introduction and experimental validation of a quantitative theoretical framework to anticipate the initial phase formed in solid-state reactions.

There has been extensive work to understand the factors that govern solid-state reaction pathways. While ab initio computations are widely used to predict the equilibrium products at a given composition (*14*), these products may not be the first to form during synthesis. Instead, the first product formed is generally the one that is kinetically most accessible, and many studies have used in situ characterization to showcase the frequent occurrence of nonequilibrium intermediate phases during solid-state synthesis (*4*–*6*). The formation of these phases is largely influenced by diffusion and nucleation, each of which presents challenges from a modeling standpoint, though some understanding has developed. For example, when ion mobility is limited, the initial reaction product is usually the one that requires the least amount of diffusion to form (*15*–*17*). Structural templating can also influence which product is the first to form, especially in cases where nucleation is a limiting factor. Previous work has shown that phases with a high degree of structural similarity to the precursors tend to have reduced nucleation barriers, encouraging their formation before the equilibrium product (*7*, *18*–*20*).

Despite the critical role of kinetics in dictating solid-state reaction pathways, there has been some evidence that diffusion and nucleation do not need to be explicitly modeled when the reaction’s thermodynamic driving force is sufficiently large (*6*, *11*). Sometimes referred to as the max-∆*G* theory, this principle states that the initial product formed between a pair of reactants will be the one that leads to the largest decrease in the Gibbs energy (∆*G*), regardless of the amount of each reactant that is present in the sample. Predictions can accordingly be made by computing ∆*G* for each possible reaction in a compositionally unconstrained manner (i.e., neglecting reactant stoichiometry) and normalizing per atom of material formed. This approach is justified by the observation that solid products tend to form locally at particle interfaces without any knowledge of the sample’s overall composition (*8*, *21*). While several in situ studies have provided support for the max-∆*G* theory (*6*, *11*), its general applicability remains largely unproven. Recent work suggests that the theory should only be applied in cases where the driving force (∆*G*) to form one phase largely exceeds that to form all other competing phases (*22*, *23*), though experimental validation is still needed to confirm this hypothesis and determine precisely under what conditions it holds.

In this work, we outline a quantitative framework based on the max-∆*G* theory to predict the initial product formed in solid-state reactions. This framework is validated and refined using experimental data collected from in situ x-ray diffraction (XRD) measurements, which we performed on a variety of samples. Eleven pairs of reactants from two chemical spaces (Li-Mn-O and Li-Nb-O) were investigated in detail by using synchrotron radiation to scan frequently and with high resolution. We also carried out a separate high-throughput study on 26 additional pairs of reactants from 12 chemical spaces. These reactions were characterized using in situ XRD measurements guided by machine learning (ML), which steered the diffractometer toward features in each pattern that facilitated the identification of reaction intermediates. By determining which phase is the first to form in each case, and comparing the result with computed reaction energies, we derive a threshold of 60 meV/atom for thermodynamic control in solid-state reactions. Our analysis suggests that the max-∆*G* theory is valid when the driving force to form one product exceeds that of all other competing phases by this proposed threshold. Combined with large-scale analysis of ab initio computed data from the Materials Project, we identify 105,652 reactions (15% of all those considered) whose initial products we suspect can be predicted using the max-∆*G* theory.

## RESULTS

### Thermodynamic versus kinetic control

The thermodynamic driving force behind a solid-state reaction is set by the change in the Gibbs energy (∆*G*) as the reactants transform into the products. This quantity has a prominent influence on the nucleation rate (*Q*) for a given product, which can be estimated using

$$Q=A\;\text{exp}\left(-\frac{16\pi \gamma^{3}}{3n^{2}k_{B}T∆G^{2}}\right)$$ (1)

In this equation from classical nucleation theory, the prefactor (*A*) depends on a variety of properties related to thermal fluctuations and diffusion rates (*24*). Although the prefactor is difficult to calculate from first principles, its magnitude is often comparable for products with similar compositions (*25*, *26*). In contrast, the exponential term can vary by several orders of magnitude, and therefore, it tends to have a greater influence on the overall nucleation rate (*27*). In addition to the atomic density (*n*), which varies little for most solids, the value of the exponential is primarily set by the product’s interfacial energy (γ), its bulk reaction energy (∆*G*), and the temperature (*T*) at which the reaction occurs. Whereas the bulk reaction energy depends on both the product and the reactants that precede it, the interfacial energy is often simplified to depend only on the product’s surface in cases where homogenous nucleation is assumed. However, heterogeneous nucleation is often prevalent in solid-state reactions, and we will discuss several cases throughout this work where the interfacial energy of a product is believed to be lowered by structural templating with its reactants.

The max-∆*G* theory suggests that when two solid phases react, they initially form the product with the largest compositionally unconstrained thermodynamic driving force (∆*G*). From inspection of the nucleation rate (Eq. 1), this theory is most likely to be valid when applied to reactions with competing products that are primarily distinguished by ∆*G*, outweighing any differences in their interfacial energies and prefactors. We therefore propose a regime for thermodynamic control, in which the driving force to form one product greatly exceeds that to form all other competing products. Recent work has shown that reactions within this regime are selective in the sense that they often lead to a high yield of the thermodynamically favored product (*22*, *23*). Here, we demonstrate that the selectivity of such reactions can be attributed to the initial formation of the product with the largest ∆*G*, bypassing all other potential intermediates. We also highlight a separate regime of kinetic control, in which two or more competing products have a comparable driving force to form. It will be shown that reactions within this regime do not have outcomes that can be predicted using the max-∆*G* theory.

**Fig. 1.**
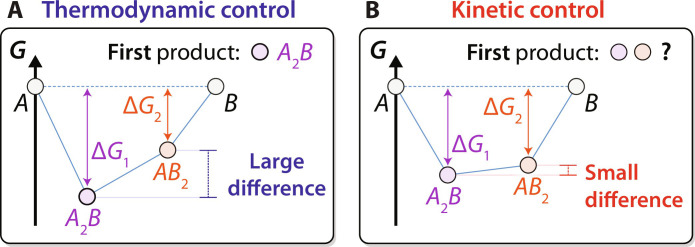
Regimes of thermodynamic and kinetic control. Two binary convex hulls are shown between arbitrary phases, *A* and *B*. Panel (**A**) illustrates the regime of thermodynamic control, where the driving force to form one phase (*A*_2_*B*) is much larger than that of the next competing phase (*AB*_2_). In this case, *A*_2_*B* can be predicted as the first phase to form. Panel (**B**) illustrates the regime of kinetic control, where each potential reaction product has a similar driving force to form. The first phase to form under kinetic control is more difficult to predict, potentially being influenced by factors related to nucleation and diffusion rates.

To schematically illustrate the proposed regimes for thermodynamic and kinetic control, we display in Fig. 1 two binary convex hulls between arbitrary reactants (denoted *A* and *B*). The left panel represents the case where the driving force (∆*G*) to form one product (*A*_2_*B*) greatly exceeds that to form any competing products (e.g., *AB*_2_), and it can therefore be predicted as the initial phase to form under the max-∆*G* theory. The right panel shows the opposite case where both the potential reaction products (*A*_2_*B* and *AB*_2_) have a comparable driving force to form, which suggests that neither can be predicted as the initial product with a high degree of confidence. Building upon the presumption that the magnitude of ∆*G* dictates whether a reaction will proceed under thermodynamic or kinetic control (*11*), we performed a series of experiments to determine how large ∆*G* must be (relative to any competing products) to ensure thermodynamic control. In its current form, this analysis only considers thermodynamically stable phases (on the convex hull) and therefore neglects the possibility of metastable phase formation.

### Synchrotron XRD on Li-Nb-O

To evaluate the behavior of solid-state synthesis reactions under thermodynamic or kinetic control, the Li-Nb-O chemical space was selected as an initial test case. It contains three well-studied ternary compounds that are reported to be thermodynamically stable in the Materials Project (*28*): LiNb_3_O_8_, LiNbO_3_, and Li_3_NbO_4_. Each compound has been previously synthesized from solid-state reactions using LiOH or Li_2_CO_3_ as the Li source and Nb_2_O_5_ as the Nb source (*29*–*33*). Analysis of the system’s reaction energies, determined using a combination of experimental and computed data (see Methods), reveals a strong thermodynamic preference to form Li_3_NbO_4_ when LiOH is used as the Li source (note S1). In contrast, the use of Li_2_CO_3_ leads to much smaller differences between the driving forces to form LiNb_3_O_8_, LiNbO_3_, or Li_3_NbO_4_. The distinct behavior of each Li source makes this a well-suited test case for our proposed framework. To this end, we performed in situ XRD measurements on two pairs of reactants: (i) two LiOH + Nb_2_O_5_ and (ii) Li_2_CO_3_ + Nb_2_O_5_. Each pair was mixed in a 1:1 Li:Nb ratio, heated to 700°C at a rate of 10°C/min, held at 700°C for 3 hours, and naturally cooled to room temperature. During this process, XRD measurements were performed at a rate of two scans per minute using beamline 12.2.2 at the Advanced Light Source (ALS; see Methods).

**Fig. 2.**
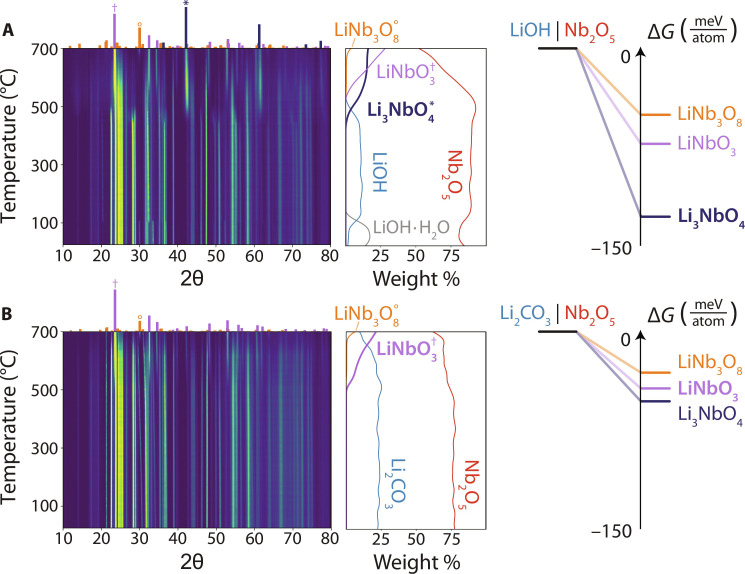
In situ XRD on reactions in the Li-Nb-O space. The pairwise reaction sequence between (**A**) LiOH and Nb_2_O_5_ is shown by the heatmap representing intensities obtained from synchrotron XRD measurements applied while heating. A second heatmap is shown for the reactions that occur between (**B**) Li_2_CO_3_ and Nb_2_O_5_. Reference XRD patterns are provided for the observed reaction products above each heatmap, which are labeled and denoted with symbols (*, °, and †) in the weight fraction plots to the right. Also shown are the reaction energies (∆*G*) to form three different ternary phases in the Li-Nb-O space when starting from LiOH as compared to Li_2_CO_3_. These energies are computed at the temperature where each Li source begins to react with Nb_2_O_5_: 450°C for LiOH and 500°C for Li_2_CO_3_.

The heatmap in Fig. 2A displays the XRD intensities obtained from the first pair of reactants (two LiOH + Nb_2_O_5_) as they were heated to 700°C. The weight fraction of each phase detected in XRD is plotted as a function of temperature to the right of the heatmap. These data show that besides the dehydration of LiOH-H_2_O near 100°C to form anhydrous LiOH, the reactants remain inert and do not decompose until 450°C, at which point they react to form Li_3_NbO_4_ (fig. S1). The computed driving force associated with Li_3_NbO_4_ formation (∆*G* = −127 meV/atom) at 450°C is much larger than that of the next most favorable product, LiNbO_3_ (∆*G* = −62 meV/atom). The observation that Li_3_NbO_4_ forms before LiNbO_3_, despite the reactants being mixed in a 1:1 Li:Nb ratio, is therefore consistent with the max-∆*G* theory. We also performed two additional experiments using samples with varied Li:Nb ratios (1:4 and 4:1) and found that Li_3_NbO_4_ was the first product to form in each case (fig. S2). The reactant stoichiometry only affected the final products, which tend to match the equilibrium phases at the sample’s overall composition. Samples with a higher Li:Nb ratio produced more Li-rich phases (Li_3_NbO_4_), whereas samples with a lower Li:Nb ratio produced more Nb-rich phases (LiNbO_3_ and LiNb_3_O_8_) in the final products.

XRD intensities obtained from the second pair of reactants (Li_2_CO_3_ + Nb_2_O_5_) during heating are shown by the heatmap in Fig. 2B. Refined weight fractions of any phases detected from these measurements are also plotted to the right of the heatmap. In contrast to the first pair of reactants, Li_2_CO_3_ and Nb_2_O_5_ initially form LiNbO_3_ when they react at 500°C (fig. S3). This outcome differs from the prediction of the max-∆*G* theory since Li_3_NbO_4_ has the largest computed driving force (∆*G* = −35 meV/atom) to form at 500°C. However, this differs only slightly from the driving force to form the observed product, LiNbO_3_ (∆*G* = −30 meV/atom). We, therefore, conclude that because the potential reaction products resulting from Li_2_CO_3_ and Nb_2_O_5_ have a comparable driving force to form, they fall within the regime of kinetic control (Fig. 1B), where the max-∆*G* theory is not expected to be applicable. This outcome is also found to be independent of the sample’s initial stoichiometry, as additional experiments performed with varied ratios of Li_2_CO_3_ and Nb_2_O_5_ still led to the formation of LiNbO_3_ (fig. S4) before any other products.

The results presented in Fig. 2B raise the question as to which kinetic factors dictate the initial reaction product of Li_2_CO_3_ and Nb_2_O_5_. While it is difficult to answer this question in a rigorous fashion using theoretical methods alone, we speculate that the interfacial energy (denoted γ in Eq. 1) plays a role in causing the reaction to deviate from the max-∆*G* theory. The interfacial energy for a product can often be lowered in cases where it heterogeneously nucleates onto another phase with a high degree of structural similarity. As a crude approximation, we quantify the similarity of LiNbO_3_ and Nb_2_O_5_ by comparing their structural fingerprints using an approach described in the Materials Project (*34*). These fingerprints were obtained using matminer and provide an average description of the nearest-neighbor coordination environments in each compound (*35*). Structural similarity is then determined by calculating the distance (L2 norm) between the fingerprints of two compounds (see Methods). Using this method, we find that LiNbO_3_ and Nb_2_O_5_ share 25% increased structural similarity than Li_3_NbO_4_ and Nb_2_O_5_. This finding is consistent with our hypothesis that structural templating enables heterogeneous nucleation of LiNbO_3_ with a lowered interfacial energy.

### Synchrotron XRD on Li-Mn-O

We next studied the validity of the max-∆*G* theory when applied to 12 pairs of reactants in the Li-Mn-O space. These include all the pairwise combinations of three Li sources (Li_2_O, LiOH, and Li_2_CO_3_) with four Mn sources (MnO, Mn_3_O_4_, Mn_2_O_3_, and MnO_2_). Each pair of reactants was mixed in a 1:1 Li:Mn ratio, heated to 1000°C at a rate of 8°C/min, and held at 1000°C for 1 hour before letting them naturally cool to room temperature. All the heating and cooling processes were performed in the air. During this time, XRD measurements were performed at a rate of one scan per minute using beamline 28-ID-2 at National Synchrotron Light Source II (NSLS-II) (see Methods). The resulting data were analyzed with Rietveld refinement to determine the first product that formed in each reaction pathway (figs. S5 to S13).

While many ternary compounds have been reported in the Li-Mn-O space, we only considered three phases when performing XRD analysis and computing reaction energies: LiMn_2_O_4_, Li_2_MnO_3_, and LiMnO_2_. These compounds are thermodynamically stable and well-characterized throughout previous work (*36*). Because they are often prepared with off-stoichiometry, most other reported ternaries in the Li-Mn-O space adopt the same structural framework as one of these three primary phases. For example, a wide variety of compositions (e.g., Li_4_Mn_5_O_12_ and Li_4_Mn_7_O_16_) have been synthesized in the LiMn_2_O_4_ spinel framework (*37*). However, it is generally difficult to distinguish these compositions by XRD alone, and therefore, we did not attempt to quantify the precise compositions of the products observed during our in situ measurements. Instead, we identified the structural framework from XRD and used it to approximate the reaction thermodynamics. For instance, the Gibbs energy of any spinel-type phase is estimated using LiMn_2_O_4_. Similar approximations are applied to structures within the Li_2_MnO_3_ and LiMnO_2_ frameworks.

**Fig. 3.**
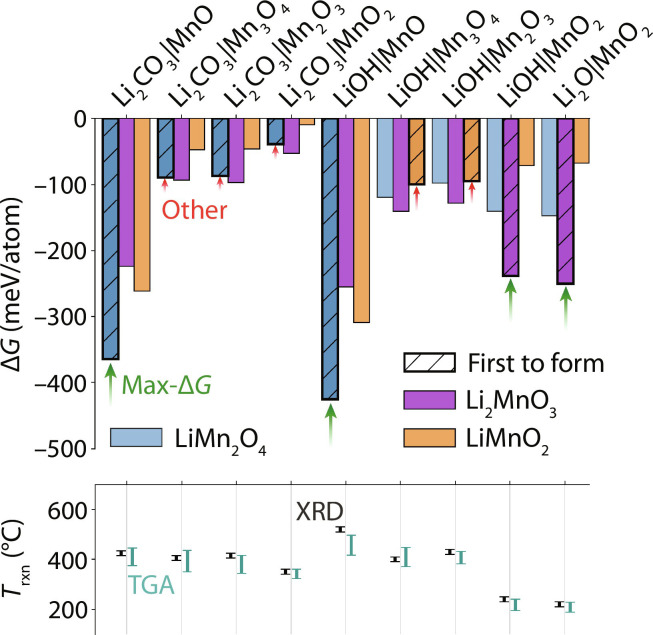
Reaction selectivity in the Li-Mn-O space. The top panel shows the driving force (∆*G*) to form each ternary phase, represented by the bar heights corresponding to different reactant combinations. Each driving force was computed at the temperature where the starting materials began to react. These onset temperatures (bottom panel) were determined using in situ XRD (left, black) and TGA (right, cyan), whose uncertainties are represented by the range of each bar (see Methods). The first product observed in the reach reaction is denoted in the top panel by hashed bars with diagonal lines. In four cases, the first product to form is the one with the largest driving force (max-∆*G*). The remaining five cases form products with a smaller driving force (other).

The driving force (∆*G*) to form each ternary phase in the Li-Mn-O space is plotted in Fig. 3 for nine of the reactant pairs that we tested. Three pairs including Li_2_O were excluded because of the formation of LiOH from reaction with humid air before any ternary product was produced (figs. S14 to S16). In four of the other nine pairs, the first product to form is the one with the largest driving force, consistent with the predictions of the max-∆*G* theory. These four cases are labeled with green arrows in Fig. 3. We also observe five separate reactions (labeled with red arrows) where the first product to form is not the one with the largest driving force. These two groups of reactions can be distinguished by their absolute and relative thermodynamic driving forces (∆*G*). The reactions whose outcomes obey the max-∆*G* theory tend to form highly exergonic products with a driving force that greatly exceeds all other competing phases. In contrast, the reactions whose outcomes deviate from the max-∆*G* theory involve products with smaller driving forces that are more comparable in magnitude.

In the bottom panel of Fig. 3, we plot the onset temperature where each pair of compounds begins to react and form a ternary product. These temperatures were determined from in situ XRD measurements and thermogravimetric analysis (TGA) measurements (see figs. S17 to S24). There appears to be some correlation between the reaction onset temperature and the choice of Li and Mn source. For instance, Mn compounds with high oxidation states (e.g., MnO_2_) generally reacted at lower temperatures than those with lower oxidation states (e.g., MnO). Similarly, pairs of reactants that included Li_2_O reacted at lower temperatures (<300°C) than pairs that included LiOH and Li_2_CO_3_, which often did not react until the samples were heated >400°C. The temperature at which these reactions occur does not appear to have any influence on whether their initial product form is the one with maximal driving force. Instead, it is only the relative driving forces among competing products that seem to dictate whether a reaction forms the phase anticipated by the max-∆*G* theory.

**Fig. 4.**
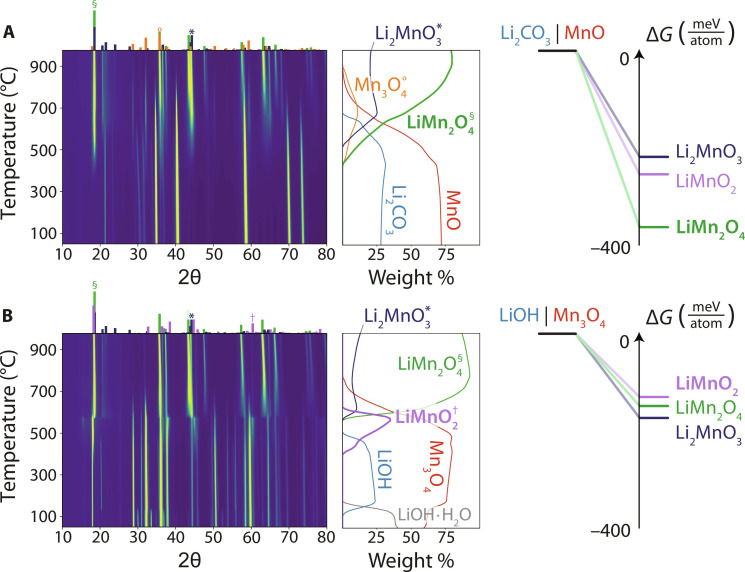
In situ XRD on reactions in the Li-Mn-O space. The pairwise reaction sequence between (**A**) Li_2_CO_3_ and MnO is shown by the heatmap representing intensities obtained from synchrotron XRD measurements applied while heating. A second heatmap is shown for the reactions that occur between (**B**) LiOH and Mn_3_O_4_. Reference XRD patterns are provided for the observed reaction products above each heatmap, which are labeled and denoted with symbols (*, °, †, and §) in the weight fraction plots to the right. Also shown are the reaction energies (∆*G*) to form three different ternary phases in the Li-Mn-O space when starting from Li_2_CO_3_ as compared to LiOH. These energies are computed at the temperature where each pair of reactants begins to form a ternary product: 420°C for LiOH|MnO and 410°C for Li_2_CO_3_|Mn_3_O_4_.

To provide more detailed examples of reactions under thermodynamic and kinetic control in the Li-Mn-O chemical space, we plot in Fig. 4 the XRD intensities obtained from two different pairs of reactants: (i) Li_2_CO_3_ + two MnO and (ii) three LiOH + Mn_3_O_4_. The first pair has a strong thermodynamic preference to form LiMn_2_O_4_ (∆*G* = −367 meV/atom) at the observed reaction temperature of 420°C. The next most favorable competing phase (LiMnO_2_) has a much smaller driving force (∆*G* = −262 meV/atom) to form at this temperature. As shown in Fig. 4A, LiMn_2_O_4_ is the first product that forms when Li_2_CO_3_ reacts with MnO at 420°C, consistent with the outcome predicted by the max-∆*G* theory. After LiMn_2_O_4_ forms as the initial product, it partially reacts with the remaining Li_2_CO_3_ to form Li_2_MnO_3_ at higher temperature, drifting toward the sample’s overall 1:1 ratio of Li:Mn.

Results from the second pair of reactants (three LiOH + Mn_3_O_4_) are shown in Fig. 4B, revealing LiMnO_2_ as the first product to form at 410°C. While there is less driving force to form this product (∆*G* = −108 meV/atom) as opposed to Li_2_MnO_3_ (∆*G* = −141 meV/atom), the difference is small, and we therefore suspect that kinetic factors may dictate the reaction’s outcome. Refinement suggests that LiMnO_2_ forms in the lithiated spinel framework (sometimes referred to as Li_2_Mn_2_O_4_) that shares the structural arrangement as Mn_3_O_4_. This differs from the ground state of LiMnO_2_, which is an orthorhombic structure with edge-sharing LiO_6_ and MnO_6_ octahedra arranged in corrugated layers (*38*). Following the methods described in the previous section, we estimate the similarity of LiMnO_2_ (in its spinel-type polymorph) and Mn_3_O_4_ by comparing their structural fingerprints. The same procedure is applied to determine the similarity of Li_2_MnO_3_ (the max-∆*G* phase) and Mn_3_O_4_. Doing so reveals that LiMnO_2_ and Mn_3_O_4_ are 38% more similar to one another than Li_2_MnO_3_ is to Mn_3_O_4_. This supports the notion that LiMnO_2_ formation may be caused by preferential nucleation from the structurally similar Mn_3_O_4_ precursor. It also provides further evidence that kinetic control becomes prevalent when two (or more) competing phases have a comparable driving force to form.

### High-throughput in situ XRD on A-M-O

To quantify the conditions that distinguish thermodynamic and kinetic control in solid-state reactions, we obtained more data by performing a series of experiments using in-house XRD with a built-in heating stage for in situ scanning (see Methods). A recently developed ML algorithm was used to guide these measurements and steer them toward the features that matter most for phase identification (*39*). Such an adaptive approach to XRD allowed all scans to be completed within 5 min, increasing the likelihood that any short-lived reaction intermediates could be detected. These scans were applied to each sample described in the next paragraph, which was heated to 600°C and held for 1 hour. The heating process involved a 5-min hold every 10°C, during which constant-temperature XRD scans were performed. Therefore, although the heating rate used between temperatures was rapid (10°C/min), the effective heating rate (after accounting for the holds) was only about 1.67°C/min. We believe that this is slow enough to allow intermediates to form before reactions at high temperatures. It also provides contrast to the faster heating rates used in our earlier experiments on Li-Nb-O (10°C/min) and Li-Mn-O (8°C/min). In cases where no reactions occurred at temperatures ≤600°C, we repeated the experiments with a higher hold temperature of 800°C (see Methods).

**Fig. 5.**
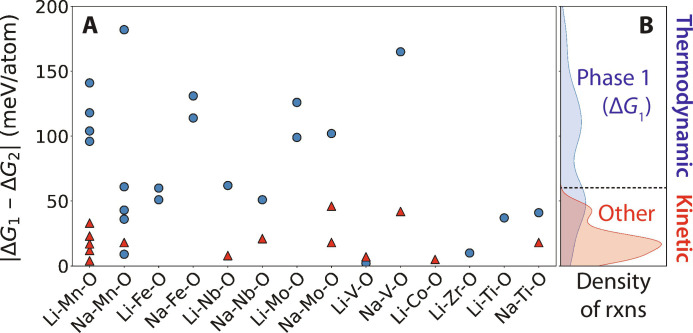
Thermodynamic versus kinetic control across alkali metal oxides. Data were collected from in situ XRD measurements performed on different pairs of alkali and transition metal reactants. (**A**) Reaction outcomes plotted as a function of the difference between the free energy of the phase with maximal driving force to form (∆*G*_1_) and the next most favorable phase (∆*G*_2_). Blue circles represent outcomes that form the max-∆*G* phase, whereas red triangles represent outcomes that form any other phase. (**B**) Kernel densities associated with reactions that do and do not form the max-∆*G* phase.

We tested 26 different pairwise combinations of reactants in the A-M-O chemical space, where A is an alkali metal (Li and Na) and M is a transition metal (Ti, V, Mn, Fe, Co, Zr, Nb, and Mo). For each alkali metal, we performed separate experiments with different sources of Li (Li_2_CO_3_ or LiOH) and Na (Na_2_CO_3_ or NaNO_3_). We also tested different sources for each transition metal, including binary metal oxides with varied oxidation states (see Methods and table S1). By varying the selection of reactants, we modified the driving force to form each possible ternary product in the corresponding A-M-O space (table S2). The experimental XRD data collected from these reactions are shown in figs. S25 to S50 with a summary of the first phases to form provided in Fig. 5, where the shape and color of each dot indicate whether the initial reaction product formed was the one with the largest driving force. Blue circles indicate the observation of products with the largest driving force to form (obeying the max-Δ*G* theory), while red triangles indicate the observation of products with less driving force to form (deviating from the max-Δ*G* theory). Data from the Li-Mn-O and Li-Nb-O reactions discussed previously are also included in Fig. 5 for comparison.

The results shown in Fig. 5 confirm that the max-∆*G* theory holds more often when the driving force to form one phase far exceeds those of any competing products. From these data, we approximate a threshold for thermodynamic control; when the driving force to form one phase exceeds all others by ≥60 meV/atom (or about 6 kJ per mole of atoms in the products), the thermodynamically preferred phase is consistently the first product to form. Of the 37 reactions tested throughout this work, 14 fall above the proposed threshold and therefore have outcomes that are predictable using the max-∆*G* theory. In contrast, alternative products that deviate from the max-Δ*G* theory are often observed when their driving force to form is within 60 meV/atom of the thermodynamically preferred product. They also tend to occur more frequently as the difference between these driving forces approaches zero. It is possible these results may depend on the heating rate used; for instance, rapid heating rates may provide less of an opportunity for intermediate phase formation. However, we do not observe any clear difference in trends between the data obtained at fast heating rates (Li-Mn-O and Li-Nb-O) and those obtained at a slow heating rate (all others).

The data presented up until this point suggest that it is the difference between the driving forces to form competing products that determines whether a reaction falls under thermodynamic or kinetic control, supporting the hypotheses made in recent work (*22*, *23*). We also compared the observed reaction outcomes with the magnitude of the driving force to form the most favorable (max-∆*G*) product, regardless of the driving force to form any competing phase. As shown in fig. S51, some correlation does exist between the magnitude of ∆*G* for a given product and whether it is the first to form in the experiment. However, it is generally less influential than the difference between ∆*G* of that product and the next most favorable one (as shown in Fig. 5). We suspect that such a correlation exists because reactions with larger ∆*G* also tend to have greater differences in their free energies, though this is not always the case. For instance, Na_4_Ti_5_O_12_ is the most favorable reaction product of NaNO_3_ and TiO_2_, with a large driving force of −241 meV/atom to form at 540°C. Yet, Na_2_Ti_3_O_7_ is observed at this temperature despite having less driving force (−220 meV/atom) to form. This outcome supports our hypothesis that small differences in relative driving forces, even when those driving forces have large magnitudes, can lead to kinetic control in solid-state reactions.

### Comparison with ab initio data

**Fig. 6.**
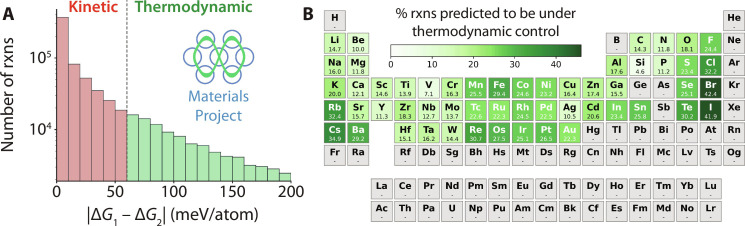
Prevalence of thermodynamic control in ab initio datasets. (**A**) Histogram showing the number of pairwise reactions available in the Materials Project, plotted with respect to the difference between the free energy of the product with the largest (∆*G*_1_) and second largest (∆*G*_2_) driving force to form. (**B**) The percentage of reactions predicted to be under thermodynamic control is shown for each element in the periodic table. Gray shading is used to signify elements that were not considered in our analysis.

On the basis of the threshold for thermodynamic control outlined in the previous section, we now broadly evaluate how many solid-state reactions have outcomes that may be predicted using the max-∆*G* theory. We considered a set of reactants composed of oxides, sulfates, phosphates, chalcogenides, carbonates, nitrates, silicates, and halides reported in the Materials Project (*28*). Our analysis only included binary (*MX*) and ternary (*M*_1_*M*_2_*X*) phases with a single anion (*X*) each. All possible pairwise combinations of these phases were enumerated, and those without any thermodynamically stable products between them were excluded, resulting in 699,189 pairs of reactants. For each pair, we identified the solid products with the largest (∆*G*_1_) and second largest (∆*G*_2_) driving force to form at a common reaction temperature of 600°C. The resulting difference in driving forces, ∣∆*G*_1_ − ∆*G*_2_∣, was then computed for each reaction. Figure 6A shows a histogram of these differences for all 699,189 reactant pairs that we considered. A dashed vertical line at 60 meV/atom represents the proposed threshold for thermodynamic control. All reactions plotted to the right of this line have ∣∆*G*_1_ − ∆*G*_2_∣> 60 meV/atom, with outcomes that we believe can be predicted using the max-∆*G* theory (i.e., the thermodynamically preferred product should be the first to form in these reactions). There exist 105,652 reactions within this regime for thermodynamic control, accounting for about 15% of all those considered.

The analysis described above includes chemistries that differ substantially from those used to develop our understanding of thermodynamic and kinetic control, which previously focused solely on the formation of ternary alkali metal oxides. As a result, the specific threshold where the max-∆*G* theory applies may vary depending on which elements and reactions are being considered. Nevertheless, the principle that reactions with greater differences in their driving forces to form competing products typically follow thermodynamic control should remain valid across chemistries. We therefore focus much of our discussion on the general trends in ∣∆*G*_1_ − ∆*G*_2_∣ throughout the periodic table, and its implications on the prevalence of thermodynamic versus kinetic control.

Figure 6B shows the occurrence of each element within compounds that participate in reactions predicted to fall under thermodynamic control. We find that elements lower on the periodic table are more often involved in reactions that have a strong thermodynamic preference to form one product. For instance, a sizeable portion (42.4 and 41.9%, respectively) of the reactions involving compounds with Br and I have ∣∆*G*_1_ − ∆*G*_2_∣> 60 meV/atom. By comparison, only 24.4 and 32.2% of reactions involving compounds with F and Cl satisfy the same constraint. Similar trends are observed in other elements as thermodynamic control appears to increase when moving down the alkali metals, alkali earth metals, and chalcogenides. We suspect that this is a function of ionic radius, as large ions are more likely to have a strong thermodynamic preference for structures that can accommodate their size. For example, ternaries containing Ba^+^ (135-pm radius) often adopt a perovskite *AB*O_3_ structure, where the *A* site has 12-fold coordination and can generally accommodate larger ions.

A second trend observed in Fig. 6B is the tendency for halides to fall under thermodynamic control more frequently than all other anions. Only 12% of reactions without any halides are predicted to have ∣∆*G*_1_ − ∆*G*_2_∣> 60 meV/atom, whereas 35% of reactions with halides satisfy this constraint. The increased prevalence of thermodynamic control for halides is likely caused by the fact that they often form strong ionic bonds in reactions that are highly exergonic (large ∆*G*). This would also explain why reactions involving alkali metals (which usually form highly ionic bonds) are more often predicted to fall under thermodynamic control than those involving alkali earth and transition metals.

Among the transition metals, it appears that reactions involving elements with partially filled d orbitals tend to have a stronger thermodynamic preference for one product than those involving d^0^ or d^10^ metals. Of all the transition metals we evaluated, Re (30.7%) and Fe (29.4%) most often participate in reactions predicted to fall under thermodynamic control. This may be related to the fact that metals with partially filled d orbitals typically have a strong preference toward one coordination environment. For example, the vast majority of Mn^4+^-containing materials adopt structures with sixfold, octahedral coordination environments (*40*) which provide a large crystal field stabilization energy for Mn^4+^ (d^3^).

One caveat of this analysis is that it may depend on how well the computed phase diagram is populated for each chemical space. Combinations of elements that are underexplored are more likely to contain reactions that we predict to fall under thermodynamic control since there are fewer products to compete with. This could provide a second explanation as to why reactions involving heavier elements (low on the periodic table) show higher percentages of thermodynamic control. Another caveat is that our analysis did not consider oxidation or decomposition reactions, whose driving force varies substantially with temperature. A more comprehensive investigation would therefore require some knowledge of these reaction temperatures.

## DISCUSSION

In this work, we studied 37 pairs of reactants using in situ characterization and ab initio computations to understand when a solid-state reaction outcome is governed primarily by thermodynamics. Our findings suggest that when the driving force to form one product exceeds all other competing phases by at least 60 meV/atom, that product can be predicted as the first one to form. The validity of this statement can be rationalized using classical nucleation theory. When the bulk reaction energies (∆*G*) of competing products outweigh any differences in their interfacial energies and pre-exponential factors, it is these reaction energies that primarily influence the products’ relative nucleation rates (Eq. 1). In contrast, kinetic factors can modify the relative nucleation rates in cases where the competing products have a comparable driving force to form. For instance, LiMnO_2_ and LiNbO_3_ were both observed as initial products (from three LiOH + Mn_3_O_4_, and Li_2_CO_3_ + Nb_2_O_5_, respectively) despite alternative compounds having larger thermodynamic driving forces to form. Their selective formation was attributed to a higher degree of structural similarity with the starting materials, which we suspect lowered the interfacial energies of these products and facilitated their nucleation. Even without explicitly modeling these kinetic factors, the proposed threshold for thermodynamic control (60 meV/atom) may be used to predict initial product formation. Of 37 reactions tested experimentally in this work, 14 satisfied this criterion, and all these reactions formed their thermodynamically preferred products regardless of their interfacial energy.

Knowledge regarding the first product that will form in a solid-state reaction sequence provides critical insight for the planning and optimization of targeted synthesis procedures. This product is the one that determines how much driving force remains to produce the desired synthesis target, and if this driving force is too low, the target’s formation can be slowed or, in some cases, prevented completely. For instance, recent studies on the synthesis of YBa_2_Cu_3_O_6 + x_ (YBCO) showed that by simply replacing the BaCO_3_ precursor with BaO_2_, the time required to form the target was reduced from >12 hours to just 30 min (*11*, *41*). In situ synchrotron XRD measurements revealed the origin of this difference, as BaO_2_ initially reacts with CuO to form an intermediate ternary phase (Ba_2_Cu_3_O_6_) that later facilitates the rapid formation of YBCO. Using the understanding developed in our work, one could predict the initial formation of Ba_2_Cu_3_O_6_ from first principles, as the driving force to form this phase exceeds all other competing Ba-Cu-O ternary phases by at least 70 meV/atom.

The importance of the first product to form in a reaction pathway is further demonstrated by several experiments reported in the current study. For example, the yield of LiNbO_3_ was shown to be highly sensitive to the choice of Li source despite all reactant combinations exhibiting a 1:1 Li:Nb ratio that matches the stoichiometry of LiNbO_3_. When starting from a mixture of LiOH and Nb_2_O_5_ (Fig. 2A), the yield of LiNbO_3_ was relatively low (62%) even after holding the sample at 700°C for 3 hours. The initial product formed (Li_3_NbO_4_) by these reactants was slow to react during this hold, preventing the growth of LiNbO_3_ and limiting its final yield (fig. S52). Density functional theory (DFT) calculations reveal only a small driving force (∆*G* = −32 meV/atom) to form LiNbO_3_ when preceded by Li_3_NbO_4_, explaining its slow growth. In contrast, when Li_2_CO_3_ and Nb_2_O_5_ were used as reactants, LiNbO_3_ formed as the initial product (Fig. 2B). This reaction sequence led to a much higher yield of 87% after holding the sample at 700°C for 3 hours (fig. S53). Its purity was limited primarily by the presence of a LiNb_3_O_8_ impurity that formed through secondary reactions between LiNbO_3_ and Nb_2_O_5_. Recent work has outlined how these secondary reactions should be avoided to achieve the maximal yield of a desired product (*23*).

The principles introduced in this work can be readily integrated with existing methods for optimizing solid-state synthesis. Two separate reports have developed algorithms that aim to select precursor combinations with favorable selectivity for a given target, which is defined as having a large thermodynamic driving force to produce that target while having a much smaller driving force to form any competing phases (*22*, *23*). Our threshold of 60 meV/atom further quantifies how large this difference must be to confidently predict the target’s initial formation. Another recently developed method, referred to as Autonomous Reaction Route Optimization with Solid-State Synthesis (ARROWS^3^) (*13*), aims to identify favorable reaction pathways with a large thermodynamic driving force at the target-forming step. It accomplishes this by creating a database of pairwise reactions based on experimental synthesis outcomes. Our work suggests that DFT-calculated thermochemical data can help inform this algorithm, contributing predicted outcomes to the pairwise reaction database in cases where such reactions fall under the regime of thermodynamic control. These predictions would pertain to initial reactions that occur between precursors, as well as to secondary reactions that occur between the intermediates that frequently appear during solid-state synthesis (*4*–*6*), although it should be noted that reactions that occur later in a synthesis pathway (after the first intermediate forms) often proceed with small driving force and, as such, are less likely to be influenced by thermodynamics alone.

Despite the progress made here, much work remains in understanding and controlling the factors that dictate solid-state reaction pathways. Our proposed threshold for thermodynamic control is a conservative estimate which was calibrated using reactions from a subset of ternary metal oxides. All reactions exceeding this threshold were confirmed to form the initial product anticipated by the max-∆*G* theory. However, we suspect that there is likely some dependence of this threshold on the elements contained in each sample. For example, some ions diffuse faster than others and therefore may be less restricted by kinetics. Relative diffusion rates could to some extent be estimated by considering the melting points of each solid reactant and referring to Tamman’s rule. The transition metal reactants included in this work span a relatively broad range of melting points (670° to 2,715°C), which are representative of many compounds used in solid-state synthesis. But to further refine the conditions for thermodynamic control, it is still necessary to collect more data from in situ measurements in diverse chemistries.

In addition to the use of in situ diffraction techniques to monitor phase evolution, it would be beneficial to characterize the particle size and morphology of any precursors used in solid-state synthesis. Previous work has demonstrated that particle size can have a substantial effect on the temperature and rate at which a reaction occurs (*42*, *43*), which can modify the relative thermodynamic driving forces of competing products. This is particularly important when gas formation is involved, in which case the entropy term (−*T*∆*S*) has a large influence over the reaction’s Gibbs energy change (table S3). Gas formation can also affect the reactivity of a given compound, whose particle size may be reduced upon decomposition. For instance, our experiments showed that MnO_2_ tends to react quickly when it forms an O_2_ byproduct, whereas Mn compounds like MnO and Mn_3_O_4_ (which require O_2_ uptake to form Mn^3+^-containing products) react more slowly. However, further effort is needed to determine how general this trend is.

Our understanding of the factors that dictate thermodynamic versus kinetic control could also be improved by considering polymorphism, as many compositions are known to exist in several different structures that are energetically similar. While solid-state reactions performed at high temperatures most often produce a thermodynamically stable structure, this is not always the case. Recent work showed that metastable polymorphs with low interfacial energies can have low nucleation barriers, allowing them to form before the ground-state polymorphs (*44*). This finding is reflected in the current study, as LiMnO_2_ was observed to form in a metastable, spinel framework when using LiOH and Mn_3_O_4_ as reactants. However, because this was the only metastable polymorph observed across the 37 reactions that we tested, it is likely reasonable to only consider products that lie on the convex hull when computing relative thermodynamic driving forces. The infrequent occurrence of metastable polymorphs in our experiments may also provide evidence that diffusion has a more dominant role in deciding reaction outcomes. Given that different polymorphs are identical in composition, the first one to form is unlikely to be dictated by factors related to diffusion. Nevertheless, more work is needed to confirm this hypothesis and advance our understanding of polymorphism. Improvements on this front will expand the number of solid-state reactions whose initial products can be predicted, thus facilitating the development of approaches for synthesis-by-design.

## METHODS

### Synchrotron measurements

All measurements involving compounds in the Li-Nb-O space were performed using beamline 12.2.2 at the ALS of Lawrence Berkeley National Laboratory (*45*). The powder samples were mixed using a Hauschild DAC mixer and then loaded into quartz capillaries with a 0.75-mm inner diameter. These capillaries were mounted into an infrared-heated SiC tube furnace at the beamline (*46*). Samples were heated in air up to 700°C at a ramp rate of 10°C/min, followed by a 3-hour hold. Diffraction data were acquired every 30 s in angle-dispersive transmission mode with a focused 25-keV monochromatic beam (λ = 0.4959 Å, 15-μm spot size) and a PILATUS3 S 1M detector. The sample-to-detector distance was calibrated using LaB_6_ (NIST_SRM_660a) placed at the sample position before each experimental run. For plotting and analysis, all data were converted into values of 2θ based on CuKα radiation (λ = 1.5406 Å).

The Li-Mn-O experiments were performed using Beamline 28-ID-2 of the NSLS-II at Brookhaven National Laboratory. The powder samples were mixed in ethanol with five 10-mm and 10 2-mm stainless balls in a 50-ml stainless steel jar using a Retsch PM200 planetary ball mill at 300 rpm for 12 hours. The slurry resulting from each sample was dried and then pressed into a pellet with a thickness of 0.5 mm and a diameter of 7 mm. These pellets were loaded into a Linkam TS1500 furnace which allows characterization by XRD while heating. A temperature ramp of 8°C/min was applied until the samples reached 1000°C. This was followed by a 1-hour hold before letting the sample cool to room temperature. During this process, diffraction patterns with a wavelength of 0.1846 Å were collected using a two-dimensional detector (Perkin-Elmer XRD 1621) that was placed 1493 mm from the sample. At each step in the heating profile, an XRD pattern was acquired once every minute. As with the Li-Nb-O data, all patterns were plotted and analyzed by first converting them into CuKα radiation (λ = 1.5406 Å). Reaction onset temperatures were determined from XRD by identifying the lowest temperature at which a ternary product can first be detected. Because each scan takes place over 8°C of continuous heating, the onset temperatures can only be determined with a precision of ±4°C. It is the average temperature in this range that is used to compute the driving force associated with each reaction.

Rietveld refinement was performed using Profex, an open-source package for the analysis of XRD patterns (*47*). During refinement, the background signal was fit using Lagrangian polynomials. As much as 1% lattice strain was allowed for each crystalline phase considered. The preferred orientation was refined using fourth-order spherical harmonics (*gewicht* = 4). Peak shapes were modeled using a convolution of Lorentzian and squared Lorentzian functions with Cauchy broadening (*rp* = 4). A uniform crystalline size (*k*_1_ = 0) in the range of *B*_1_ = 0.0 to 0.1 was assumed. Microstrain was not accounted for in any of the samples (*k*_2_ = 0) to avoid overfitting. We also refine neither atomic positions nor thermal displacement parameters. The weight fraction of each phase was calculated by normalizing the scaling factor (*gewicht*) of all constituent phases in each pattern.

### In-house measurements

A powder sample for each pair of reactants listed in table S1 was prepared in a 1:1 ratio of alkali to transition metal. We only considered materials that were sufficiently stable in the air to not fully oxidize or react with H_2_O/CO_2_ well below their reaction temperatures. This led to the exclusion of certain transition metal reactants, such as V_2_O_3_ (which readily oxidizes in air) (*48*) and CoO (which reacts with water vapor) (*49*). All reactants we tested were mixed with ethanol and milled for 10 min using a SPEX 800 mixer. The mixed slurries were then dried in air at 70°C for 1 hour. After drying, each sample was loaded into the Anton Paar HTK 1200N oven chamber of a Bruker D8 ADVANCE x-ray diffractometer. Heating was performed at a rate of 10°C/min up to 600°C for most samples. During this process, XRD scans were carried out once every 10°C for all temperatures ≥100°C. The measurements were guided by ML algorithms to ensure that more time was spent scanning the angles (2θ) with features that were most relevant for phase identification. Further details on this ML-driven approach can be found in previous work (*39*). On the basis of the results of these measurements, we identified one case (Li_2_CO_3_ and ZrO_2_) where no reactions occurred at temperatures ≤600°C. The sample was therefore prepared again using the same procedure and then heated to 800°C, while in situ XRD scans were carried out. The patterns acquired from all the samples were analyzed using a previously developed ML package (XRD-AutoAnalyzer) (*50*), followed by Rietveld refinement using the same procedure described in the previous section. The initial product formed in each case is listed in table S2.

We performed TGA measurements using a TGA5500 (TA Instruments) with an alumina pan, with a 10°C/min heating rate in air. The temperature at which each reaction occurs is estimated from TGA by detecting the onset of weight loss from the sample, which corresponds to the beginning of decomposition reactions through O_2_, CO_2_, or H_2_O evolution. Because decomposition occurs continuously during heating, we estimate the uncertainty of each reaction onset temperature based on the range of temperatures over the weight loss curve is nonlinear (fig. S54).

### Computed driving forces

Our method of computing the driving force associated with each reaction considered here follows a similar procedure to that outlined in previous work (*51*). Experimentally determined formation enthalpies were used for any solid reactants and products that are reported in the National Institute of Standards and Technology (NIST) database (*52*). For solid phases without experimental data available, we used formation energies obtained from the Materials Project (*28*). These energies were determined using DFT calculations based on the r^2^SCAN functional (*53*). Finite temperature effects were accounted for by using a machine-learned descriptor of the vibrational entropy (*54*). We also approximated the effects of configurational entropy on a known disordered phase, Li_3_NbO_4_, by using an ideal solution model. These contributions were summed to obtain the temperature-dependent Gibbs energy of each solid phase. Gaseous byproducts (O_2_, CO_2_, N_2_, and H_2_O) required to balance certain reactions (e.g., with carbonates, nitrates, or hydroxides) were accounted for using experimental free energies reported in the NIST database (*52*). These were determined on the basis of the partial pressure of each species in the air: 21,280 Pa for O_2_, 40 Pa for CO_2_, 79,030 Pa for N_2_, and 1010 Pa for H_2_O.

The driving force of each reaction was calculated by taking the difference between the Gibbs energy of the products and reactants at the temperature where that reaction was observed. We assume the sample and furnace temperatures are equal, neglecting any self-heating effects that may arise from exothermic reactions. While such effects can be substantial in cases where the enthalpy change is large (e.g., on the order of ~1000 meV/atom), most of the reactions considered in our work display more modest enthalpy changes (table S3). We also used small sample quantities (≤2 g) to allow fast heat transfer with the surroundings. In cases where more than one reactant was present for a single element (e.g., due to partial oxidation of MnO to Mn_3_O_4_), only the reactant with the larger weight fraction was used to compute the driving force. We also only considered reactions that included a single solid product when assessing relative thermodynamic driving forces. These driving forces were normalized by the total number of atoms in the reaction product(s) formed.

### Structural similarity

Our approach used to estimate the interfacial energy between two compounds involves measuring their structural similarity. To accomplish this, we first iterate through all the occupied sites in a structure and determine the nearest neighbors surrounding each site using the CrystalNN method (*34*). A set of descriptors is then generated for each coordination environment. These descriptors include features related to the coordination number (i.e., how many nearest neighbors each site has) and geometry (tetrahedral, octahedral, etc.), and further details on each descriptor are provided in the documentation for matminer (*35*). After gathering a set of descriptors for each site in a given structure, its “fingerprint” (***v***) is constructed by computing the minimum, maximum, mean, and SD in the sites’ descriptors. This approach can be used to generate fingerprints for two different structures (*i* and *j*), and their similarity can then be quantified by taking the L2 norm of their difference

$$|v_{i}-v_{j}|=\sqrt{\sum_{n}{\left(v_{i}^{n}-v_{j}^{n}\right)}^{2}}$$ (2)

where *n* iterates over all the descriptors present in each fingerprint. The resulting metric is unitless and has no direct physical interpretation; however, lower values generally indicate increased structural similarity. More details on the process of computing structural similarity can be found in the Materials Project documentation (*34*).

## Supplementary Materials

### This PDF file includes:

Note S1 Figs. S1 to S54 Tables S1 to S3 Legends for data S1 and S2
