# Supplementary material for: Quantifying the regime of thermodynamic control for solid-state reactions during ternary metal oxide synthesis
Source: Sci Adv. 2024 Jul 3;10(27):eadp3309. doi: 10.1126/sciadv.adp3309 (PMC11221506; doi:10.1126/sciadv.adp3309)
Supplement: Supplementary file 1 — Note S1 Figs. S1 to S54 Tables S1 to S3 Legends for data S1 and S2 [file sciadv.adp3309_sm.pdf]

Science Advances
AAAS

Supplementary Materials for

Quantifying the regime of thermodynamic control for solid-state reactions during ternary metal oxide synthesis

Nathan J. Szymanski *et al.*

Corresponding author: Haegyeom Kim, haegyumkim@lbl.gov; Gerbrand Ceder, gceder@berkeley.edu

Sci. Adv. **10**, eadp3309 (2024)
DOI: 10.1126/sciadv.adp3309

The PDF file includes:

Note S1
Figs. S1 to S54
Tables S1 to S3
Legends for data S1 and S2

Other Supplementary Material for this manuscript includes the following:

Data S1 and S2

Supplementary Note 1

The use of LiOH as a reactant tends to increase the driving force to form Li-rich compounds. For example, LiOH and Nb_2O_5 have a large driving force to form Li_3NbO_4 at the observed reaction temperature of 450 °C:

$$6\ LiOH + Nb_2O_5 \rightarrow 2\ Li_3NbO_4 + 3\ H_2O\ (\Delta G = –127\ \text{meV/atom})$$

Whereas the two competing products with lower Li content have much less thermodynamic incentive to form at the same reaction temperature:

$$2\ LiOH + Nb_2O_5 \rightarrow 2\ LiNbO_3 + H_2O\ (\Delta G = –62\ \text{meV/atom})$$

$$2\ LiOH + 3\ Nb_2O_5 \rightarrow 2\ LiNb_3O_8 + 2\ H_2O\ (\Delta G = –32\ \text{meV/atom})$$

If we instead consider the reactions needed to form these products using Li_2CO_3, there exists less preference toward the formation of Li-rich phases. In this case, all three ternary products have a comparable driving force to form at the observed reaction temperature of 500 °C:

$$3\ Li_2CO_3 + Nb_2O_5 \rightarrow 2\ Li_3NbO_4 + 3\ CO_2\ (\Delta G = –35\ \text{meV/atom})$$

$$Li_2CO_3 + Nb_2O_5 \rightarrow 2\ LiNbO_3 + CO_2\ (\Delta G = –30\ \text{meV/atom})$$

$$Li_2CO_3 + 3\ Nb_2O_5 \rightarrow 2\ LiNb_3O_8 + CO_2\ (\Delta G = –23\ \text{meV/atom})$$

This difference originates from the contrasting thermodynamic properties of each Li source. Under standard conditions, Li_2CO_3 has a much lower Gibbs energy of formation ($\Delta G_\mathrm{f}^0 = -1.94$ eV/atom) than LiOH ($\Delta G_\mathrm{f}^0 = -1.52$ eV/atom). Because we define the driving of a reaction as the change in its Gibbs energy as the reactants transform to the products, reactions that consume large amounts of LiOH will be more favorable than those that consume large amounts of Li_2CO_3. In other words, one can increase the driving force to form a Li-rich product by using LiOH instead of Li_2CO_3.

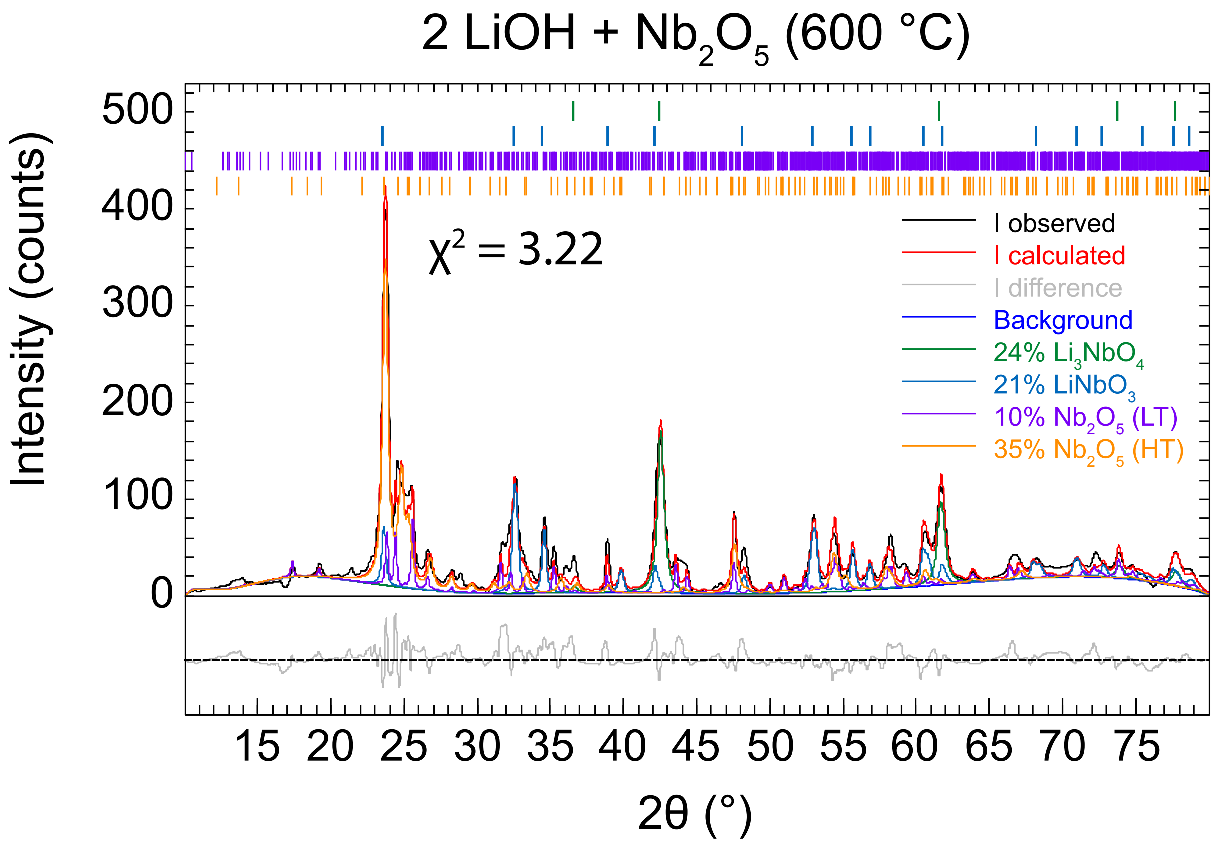

Supplementary Fig. 1 | Refinement of the products initially formed between LiOH and Nb_2O_5. Rietveld refinement performed on the XRD pattern collected at 600 °C from the sample initially containing LiOH and Nb_2O_5 in 2:1 molar ratio. Two polymorphs of Nb_2O_5 were observed: the low-temperature (LT) polymorph (P2/m) and the high-temperature (HT) polymorph (I4/mmm). At 600 °C, the majority products are Li_3NbO_4 and $LiNbO_3$. The percentage listed next to each phase corresponds to its weight fraction in the sample.

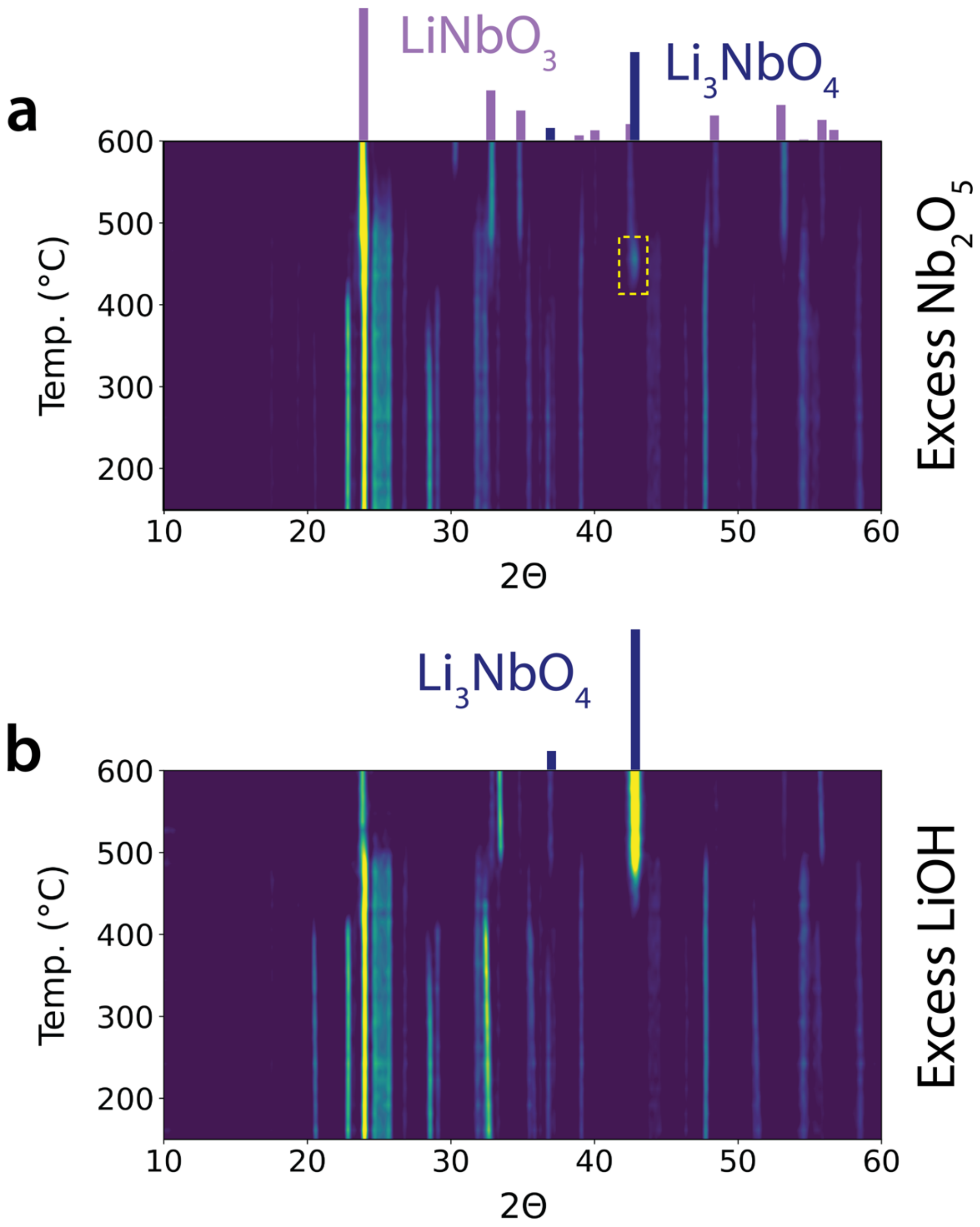

Supplementary Fig. 2 | *In-situ* characterization of LiOH-Nb_2O_5 with varied reactant amounts. (**a**) The first heatmap shows the temperature dependent XRD intensities measured from a sample containing LiOH and Nb_2O_5, mixed in a 1:4 ratio of Li to Nb. (**b**) The second heatmap shows the temperature dependent XRD intensities measured from a sample containing LiOH and Nb_2O_5, mixed in a 4:1 ratio of Li to Nb. In each case, Li_3NbO_4 is observed as the initial product, and its reference pattern is plotted above the heatmaps. This product remains the majority phase in the sample containing excess Li, whereas it appears only as a transient intermediate in the sample containing excess Nb. In this case, its largest peak is highlighted by the yellow dashed box.

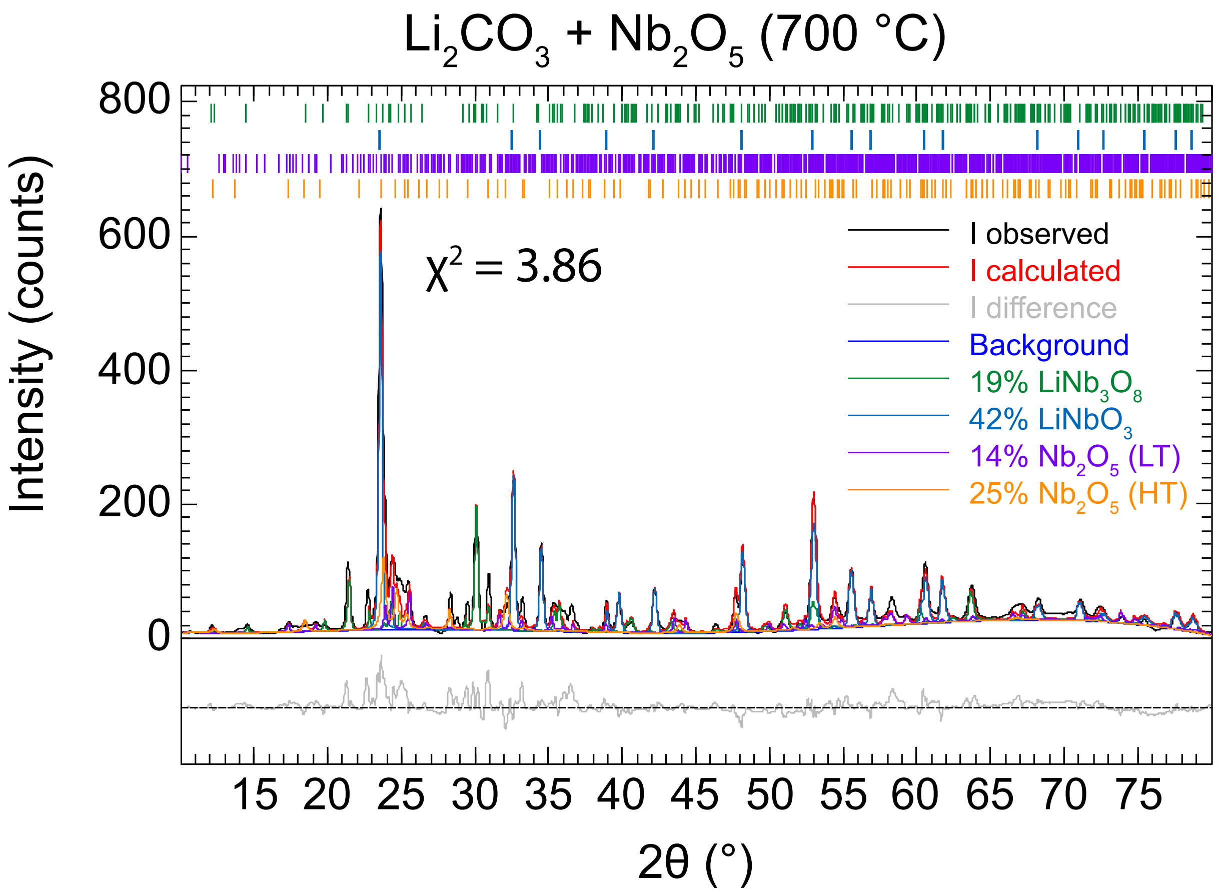

Supplementary Fig. 3 | Refinement of the products initially formed between Li_2CO_3 and Nb_2O_5. Rietveld refinement performed on the XRD pattern collected at 700 °C from the sample initially containing Li_2CO_3 and Nb_2O_5 in 1:1 molar ratio. Two polymorphs of Nb_2O_5 were observed: the low-temperature (LT) polymorph (P2/m) and the high-temperature (HT) polymorph (I4/mmm). At 700 °C, the majority products are $LiNbO_3$ and $LiNb_3O_8$. The percentage listed next to each phase corresponds to its weight fraction in the sample.

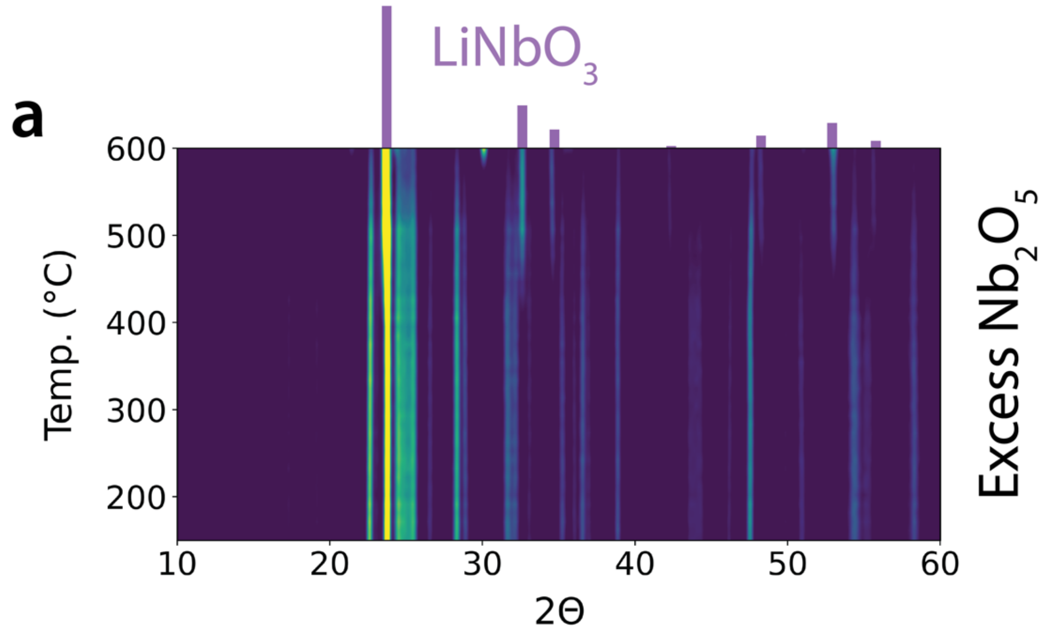

Supplementary Fig. 4 | *In-situ* characterization of Li_2CO_3-Nb_2O_5 with varied reactant amounts. (**a**) The first heatmap shows the temperature dependent XRD intensities measured from a sample containing Li_2CO_3 and Nb_2O_5, mixed in a 1:4 ratio of Li to Nb. (**b**) The second heatmap shows the temperature dependent XRD intensities measured from a sample containing Li_2CO_3 and Nb_2O_5, mixed in a 4:1 ratio of Li to Nb. In each case, $LiNbO_3$ is observed as the initial product, and its reference pattern is plotted above the heatmaps. A partial transformation of $LiNbO_3$ to $LiNb_3O_8$ is later observed near 600 °C in the sample containing Nb_2O_5.

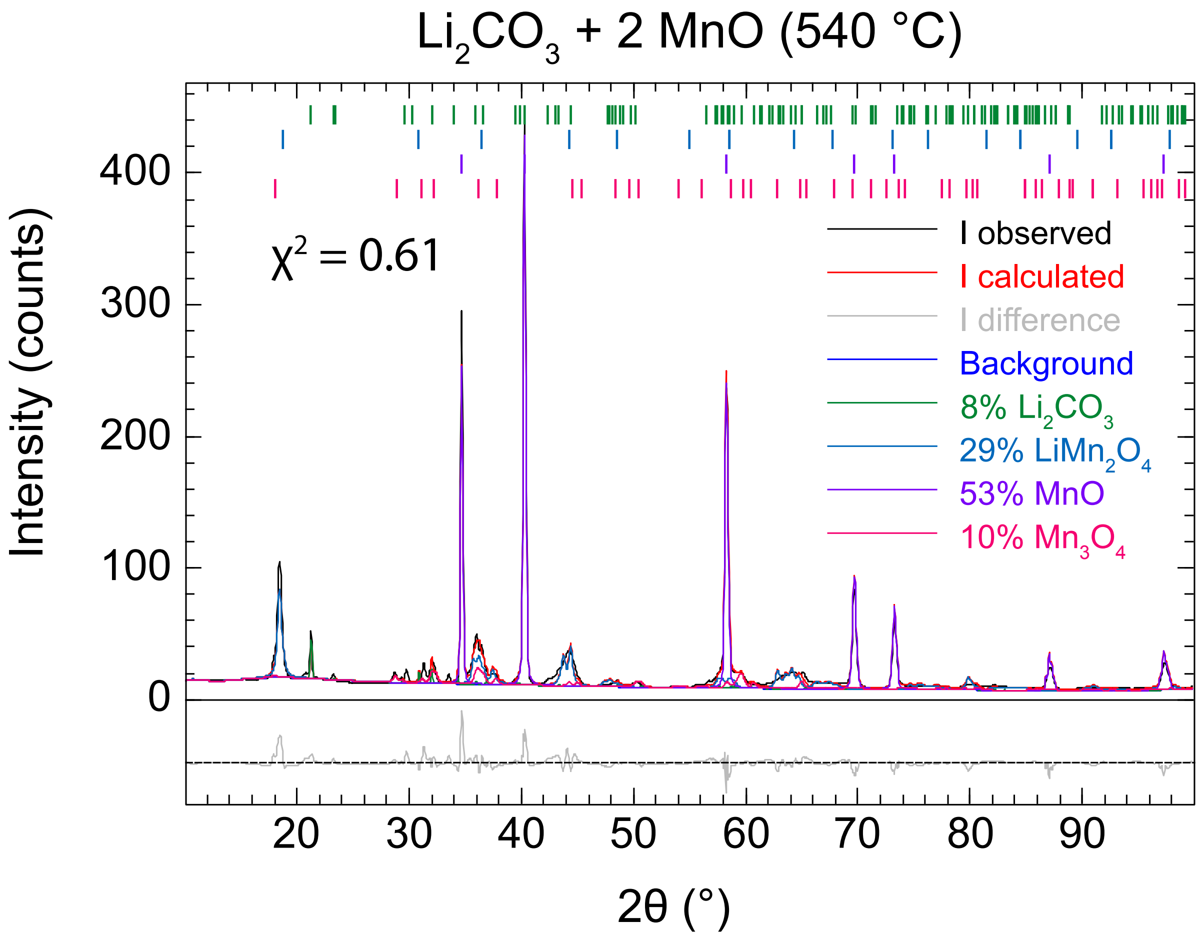

Supplementary Fig. 5 | Refinement of the products initially formed between Li_2CO_3 and MnO. Rietveld refinement performed on the XRD pattern collected at 540 °C from the sample initially containing Li_2CO_3 and MnO in 1:2 molar ratio. At this temperature, $LiMn_2O_4$ is the majority product. The percentage listed next to each phase corresponds to its weight fraction in the sample.

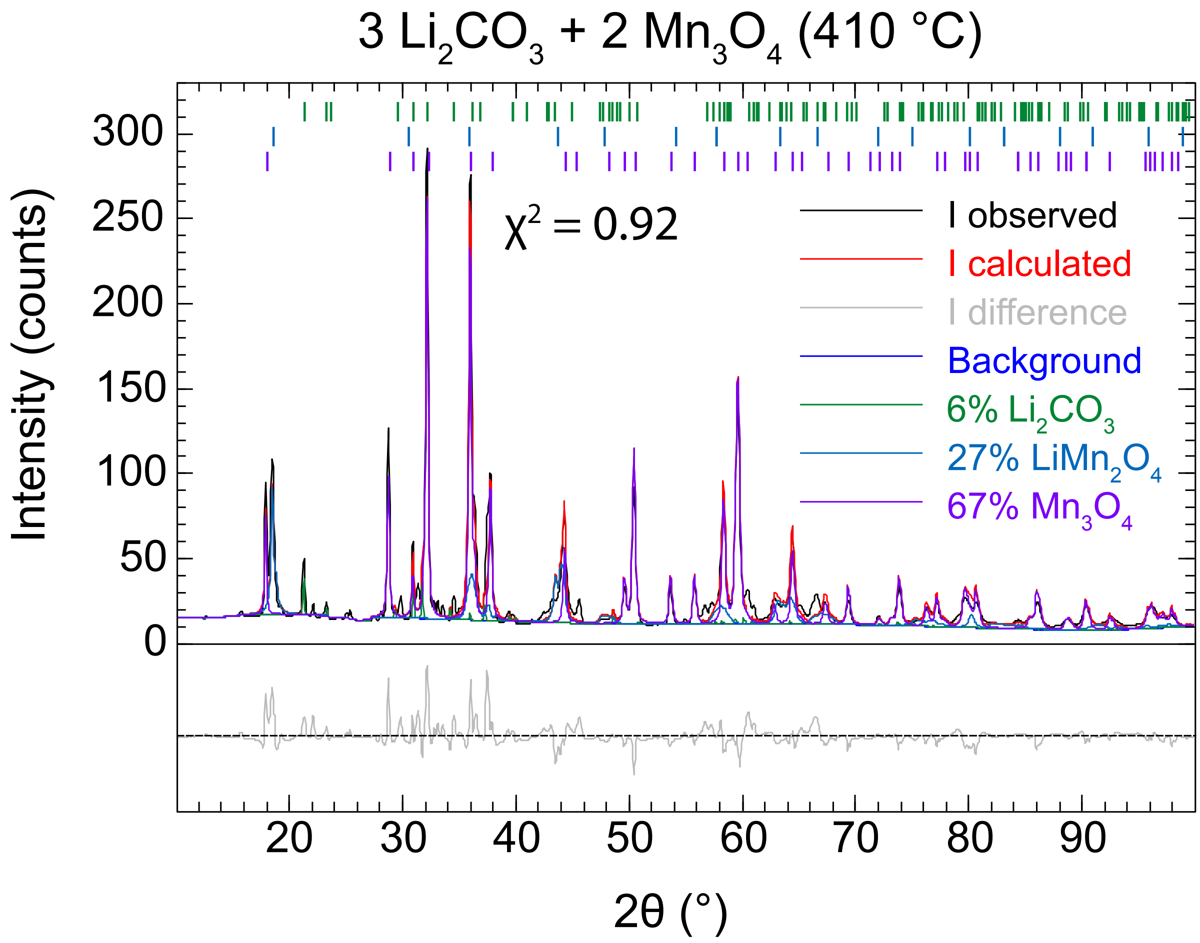

Supplementary Fig. 6 | Refinement of the products initially formed between Li_2CO_3 and Mn_3O_4. Rietveld refinement performed on the XRD pattern collected at 410 °C from the sample initially containing Li_2CO_3 and Mn_3O_4 in 3:2 molar ratio. At this temperature, $LiMn_2O_4$ is the majority product. The percentage listed next to each phase corresponds to its weight fraction in the sample.

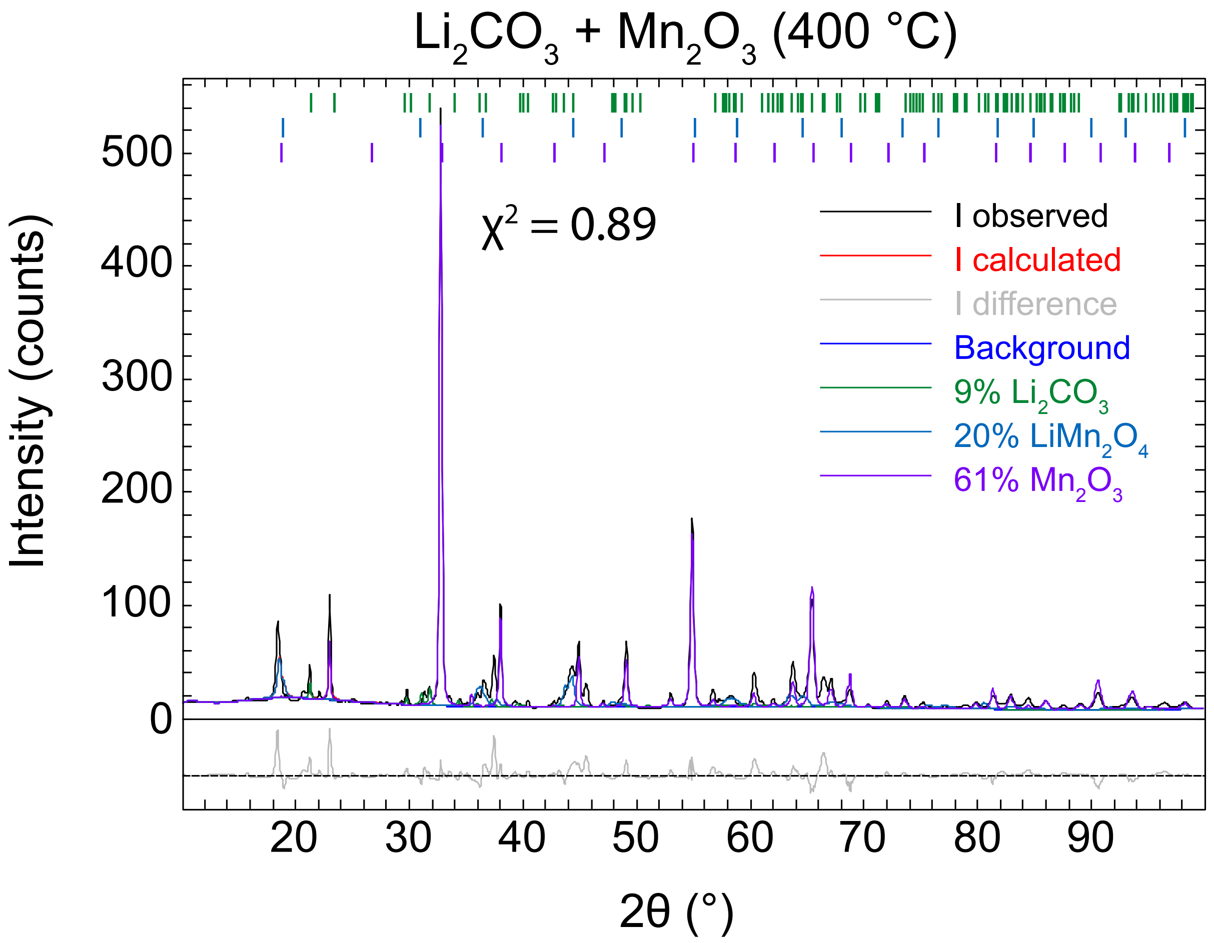

Supplementary Fig. 7 | Refinement of the products initially formed between Li_2CO_3 and Mn_2O_3. Rietveld refinement performed on the XRD pattern collected at 400 °C from the sample initially containing Li_2CO_3 and Mn_2O_3 in 1:1 molar ratio. At this temperature, $LiMn_2O_4$ is the majority product. The percentage listed next to each phase corresponds to its weight fraction in the sample.

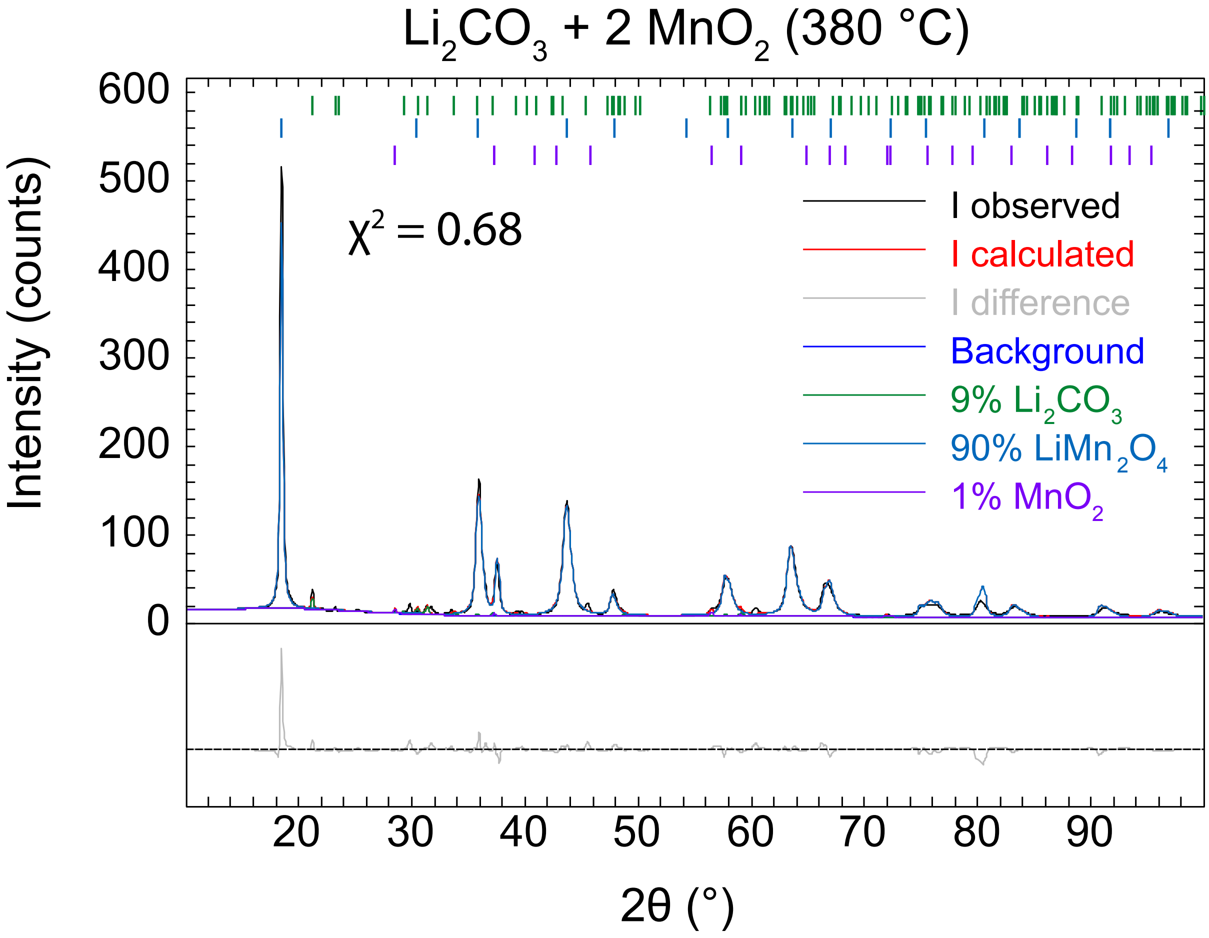

Supplementary Fig. 8 | Refinement of the products initially formed between Li_2CO_3 and MnO_2. Rietveld refinement performed on the XRD pattern collected at 380 °C from the sample initially containing Li_2CO_3 and MnO_2 in 1:2 molar ratio. At this temperature, $LiMn_2O_4$ is the majority product. The percentage listed next to each phase corresponds to its weight fraction in the sample.

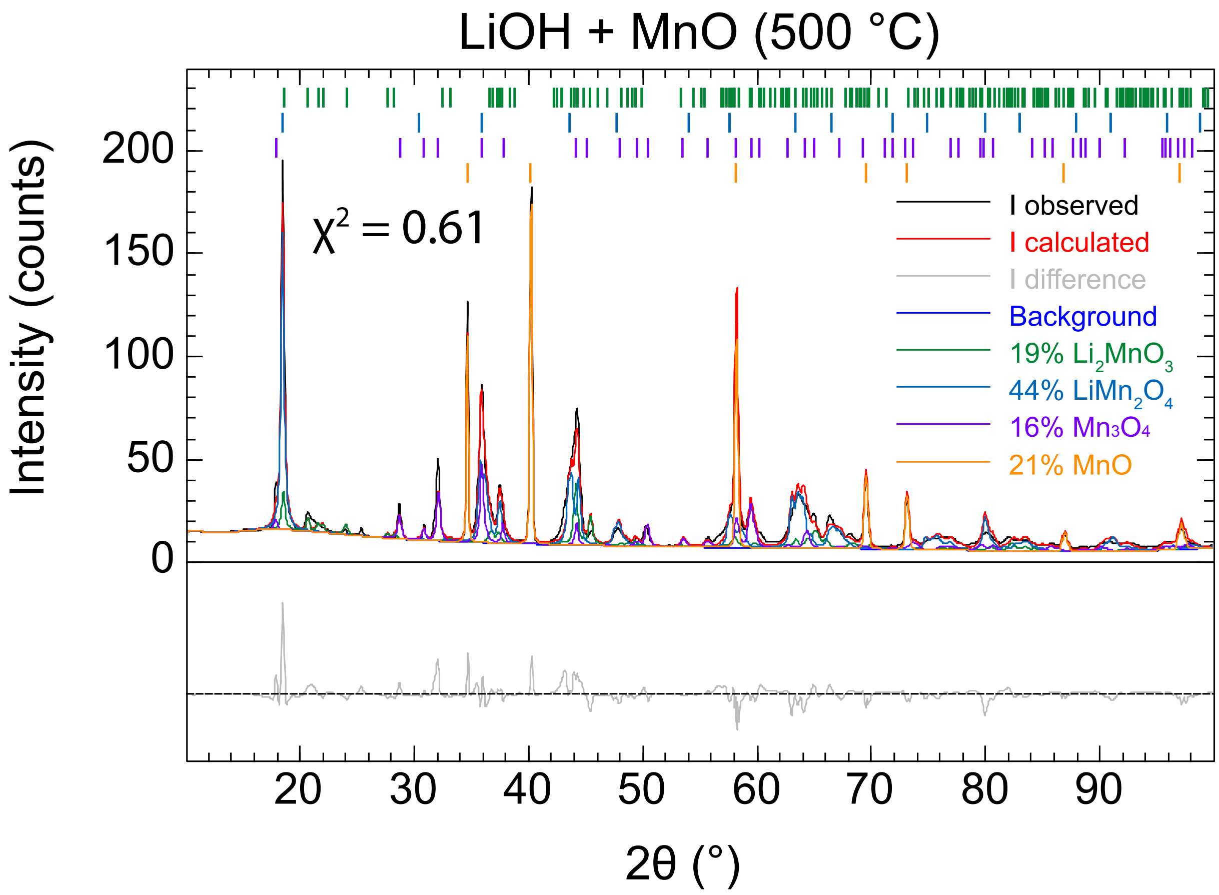

Supplementary Fig. 9 | Refinement of the products initially formed between LiOH and MnO. Rietveld refinement performed on the XRD pattern collected at 500 °C from the sample initially containing LiOH and MnO in 1:1 molar ratio. At this temperature, $LiMn_2O_4$ and Li_2MnO_3 are the majority products. The percentage listed next to each phase corresponds to its weight fraction in the sample.

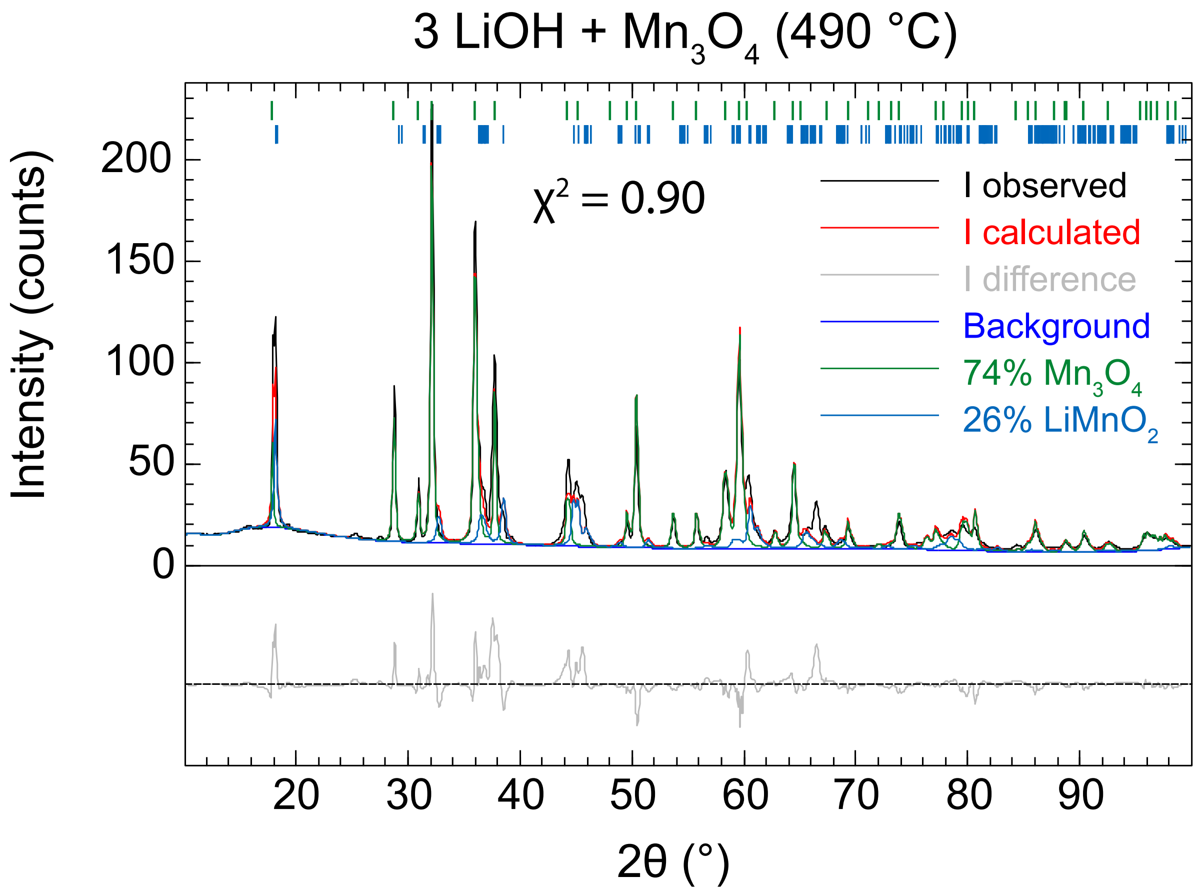

Supplementary Fig. 10 | Refinement of the products initially formed between LiOH and Mn_3O_4. Rietveld refinement performed on the XRD pattern collected at 490 °C from the sample initially containing LiOH and Mn_3O_4 in 3:1 molar ratio. At this temperature, $LiMnO_2$ is the majority product. The percentage listed next to each phase corresponds to its weight fraction in the sample.

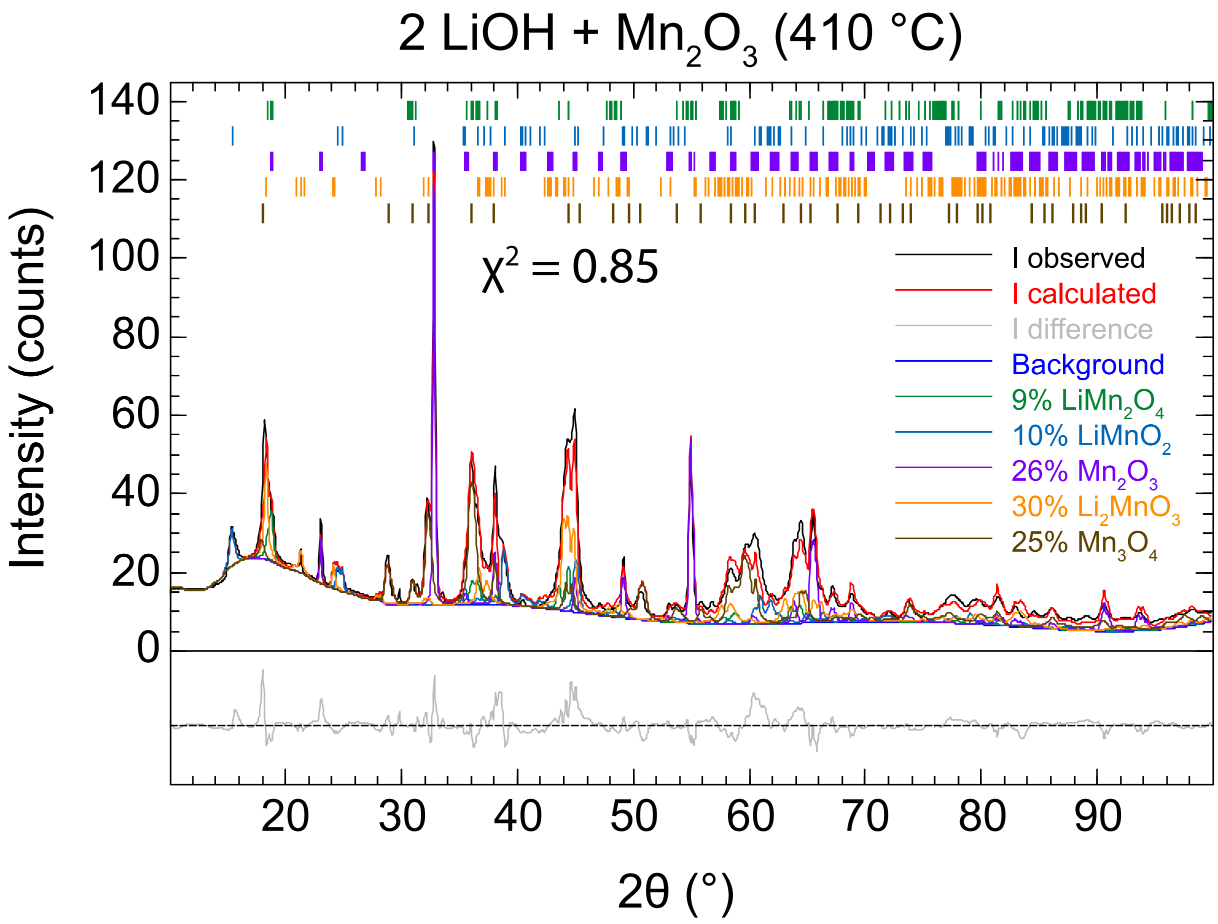

Supplementary Fig. 11 | Refinement of the products initially formed between LiOH and Mn_2O_3. Rietveld refinement performed on the XRD pattern collected at 410 °C from the sample initially containing LiOH and Mn_2O_3 in 2:1 molar ratio. $LiMnO_2$, $LiMn_2O_4$, and Li_2MnO_3 are the majority products at this temperature. The percentage listed next to each phase corresponds to its weight fraction in the sample.

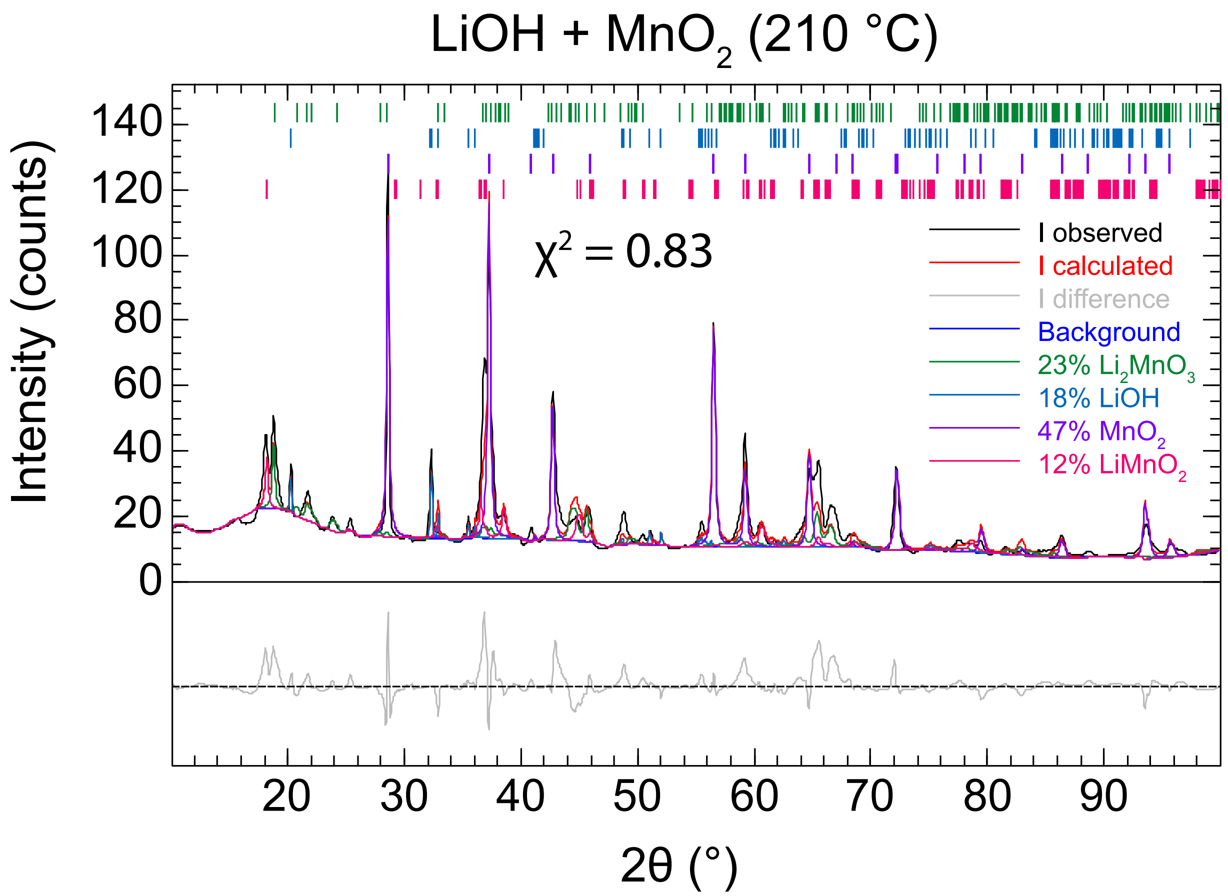

Supplementary Fig. 12 | Refinement of the products initially formed between LiOH and MnO_2. Rietveld refinement performed on the XRD pattern collected at 210 °C from the sample initially containing LiOH and MnO_2 in 1:1 molar ratio. At this temperature, Li_2MnO_3 is the majority product. The percentage listed next to each phase corresponds to its weight fraction in the sample.

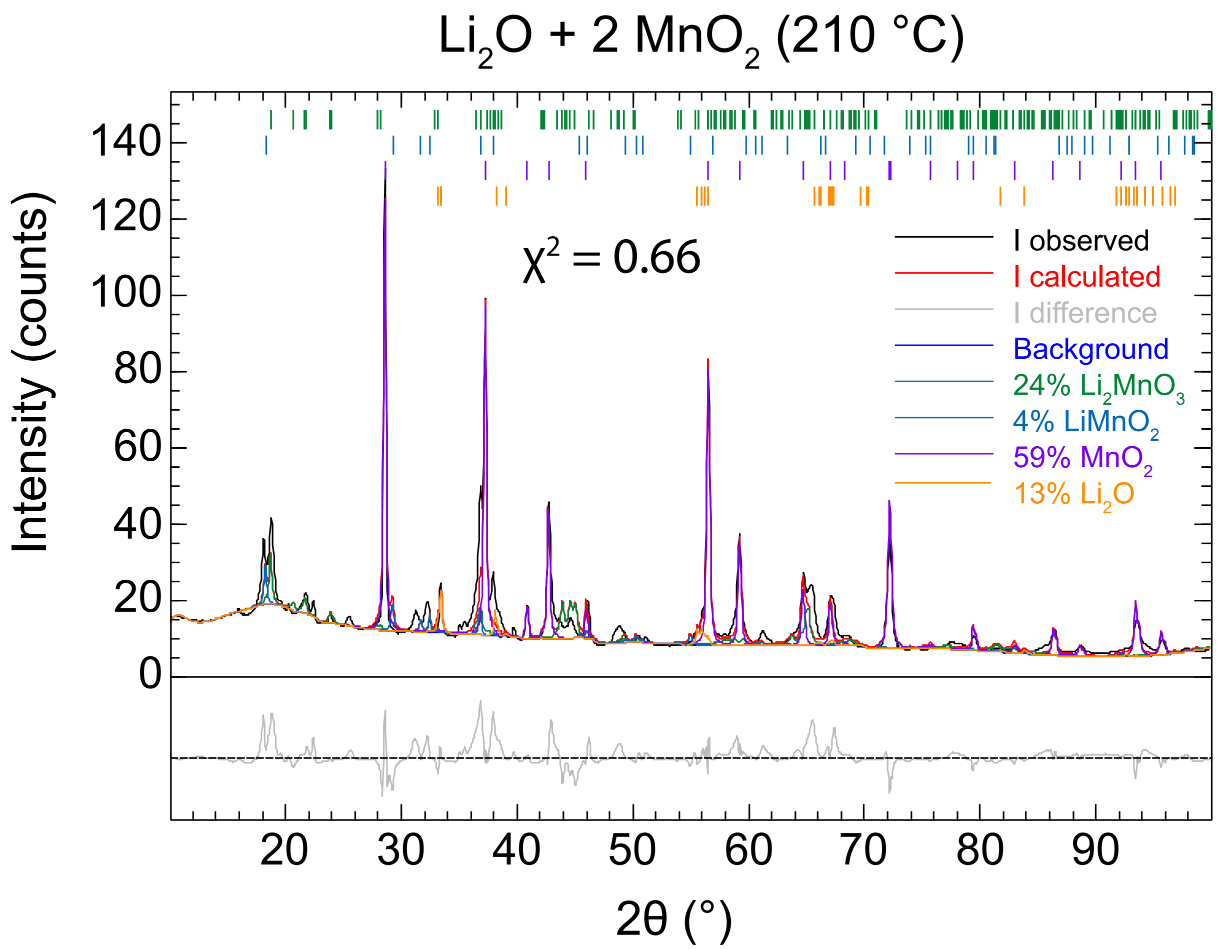

Supplementary Fig. 13 | Refinement of the products initially formed between Li_2O and MnO_2. Rietveld refinement performed on the XRD pattern collected at 210 °C from the sample initially containing Li_2O and MnO_2 in 1:2 molar ratio. At this temperature, Li_2MnO_3 and $LiMnO_2$ are the majority products. The percentage listed next to each phase corresponds to its weight fraction in the sample.

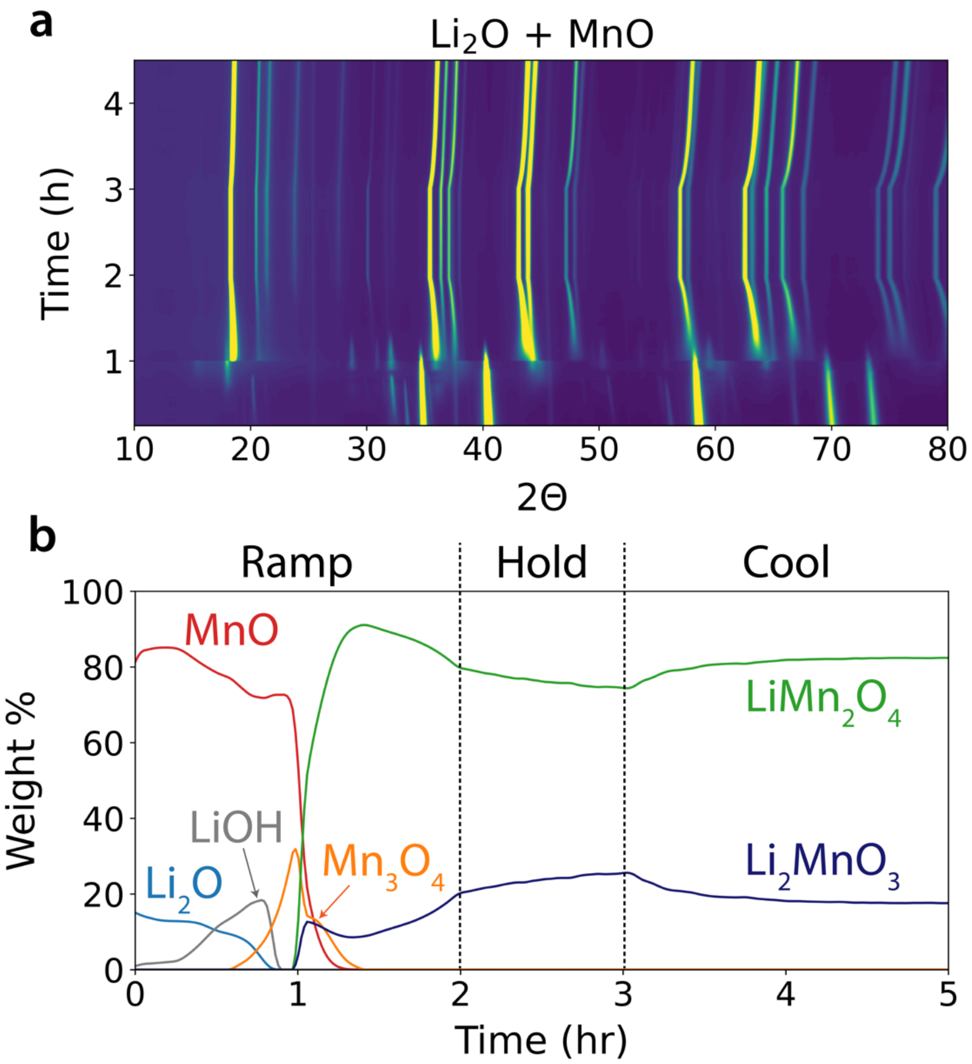

Supplementary Fig. 14 | *In-situ* characterization of the reaction sequence for Li_2O and MnO. (**a**) The heatmap shows the time dependent XRD intensities measured from a sample containing Li_2O and MnO, mixed in a 1:1 ratio of Li to Mn. The sample was heated from room temperature to 1000 °C during the ramp, held at 1000 °C for 1 h, then naturally cooled. (**b**) The weight fractions of all phases detected from the XRD measurements are also plotted as a function of time.

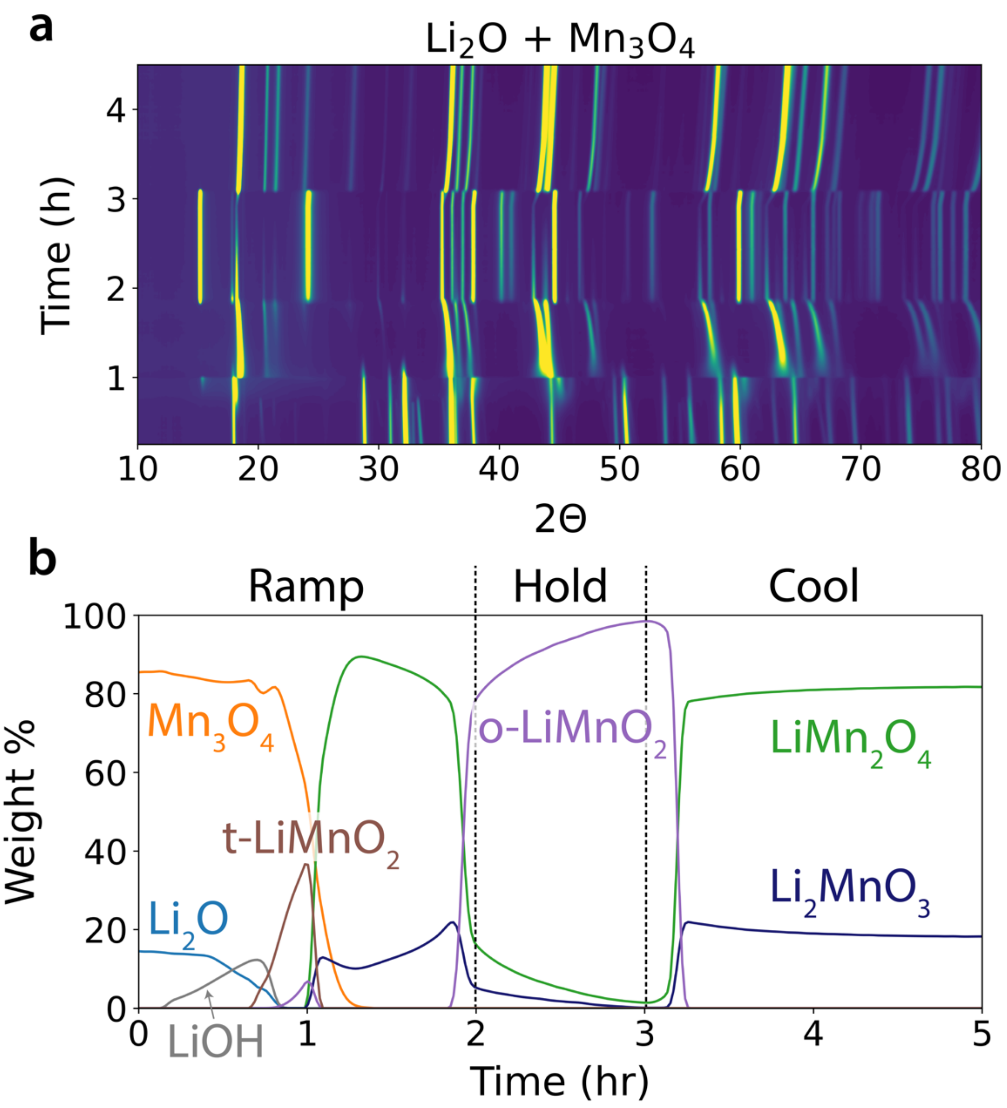

Supplementary Fig. 15 | *In-situ* characterization of the reaction sequence for Li_2O and Mn_3O_4. (**a**) The heatmap shows the time dependent XRD intensities measured from a sample containing Li_2O and Mn_3O_4, mixed in a 1:1 ratio of Li to Mn. The sample was heated from room temperature to 1000 °C during the ramp, held at 1000 °C for 1 h, then naturally cooled. (**b**) The weight fractions of all phases detected from the XRD measurements are also plotted as a function of time.

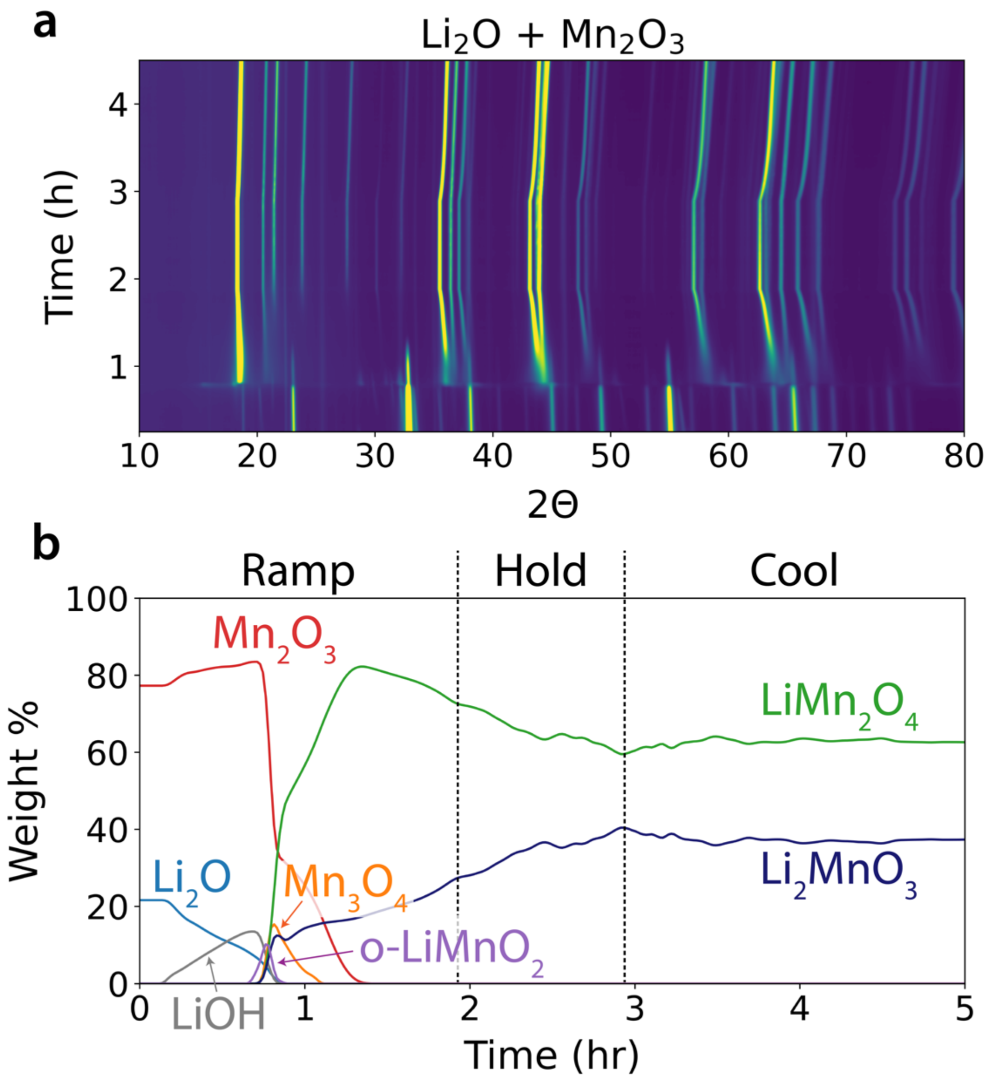

Supplementary Fig. 16 | *In-situ* characterization of the reaction sequence for Li_2O and Mn_2O_3. (**a**) The heatmap shows the time dependent XRD intensities measured from a sample containing Li_2O and Mn_2O_3, mixed in a 1:1 ratio of Li to Mn. The sample was heated from room temperature to 1000 °C during the ramp, held at 1000 °C for 1 h, then naturally cooled. (**b**) The weight fractions of all phases detected from the XRD measurements are also plotted as a function of time.

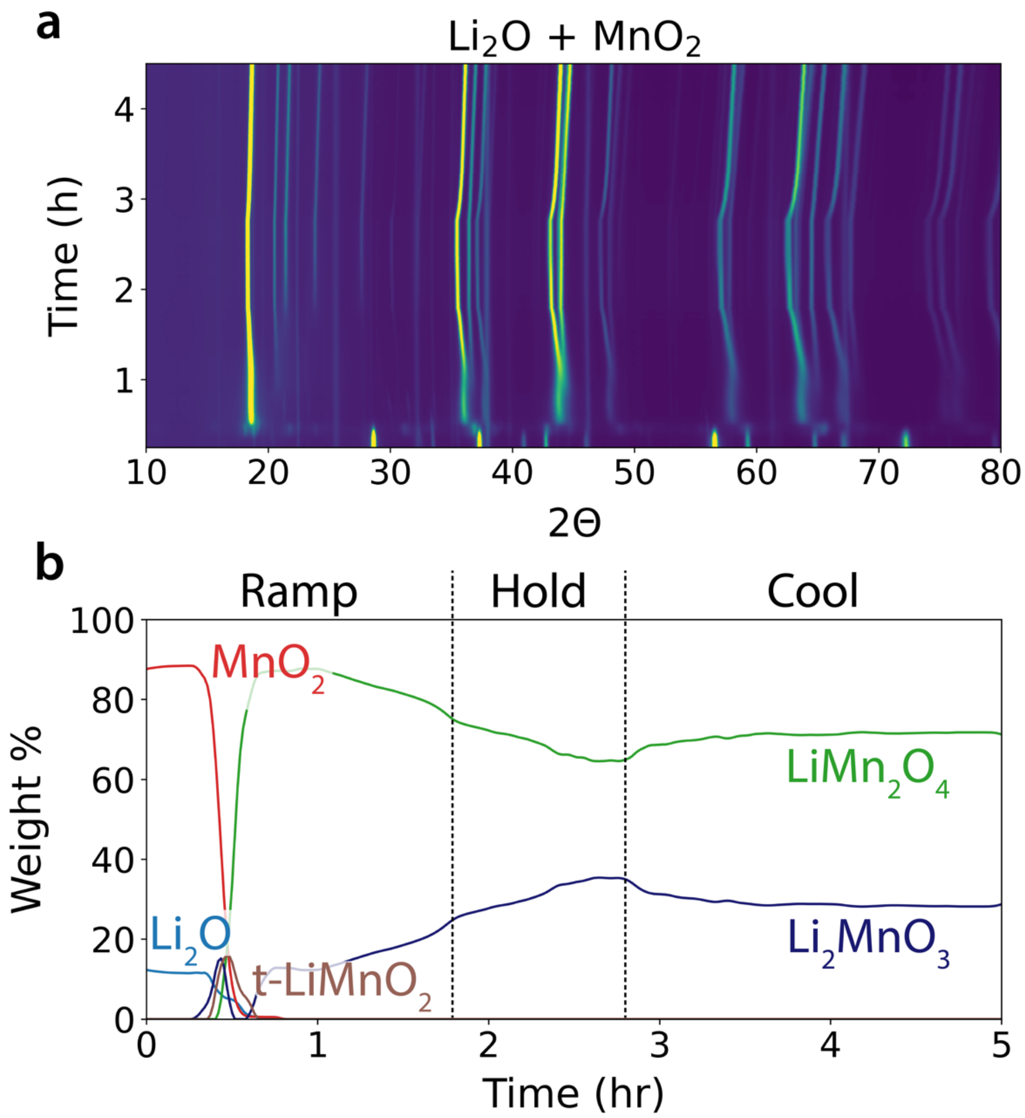

Supplementary Fig. 17 | *In-situ* characterization of the reaction sequence for Li_2O and MnO_2. (**a**) The heatmap shows the time dependent XRD intensities measured from a sample containing Li_2O and MnO_2, mixed in a 1:1 ratio of Li to Mn. The sample was heated from room temperature to 1000 °C during the ramp, held at 1000 °C for 1 h, then naturally cooled. (**b**) The weight fractions of all phases detected from the XRD measurements are also plotted as a function of time.

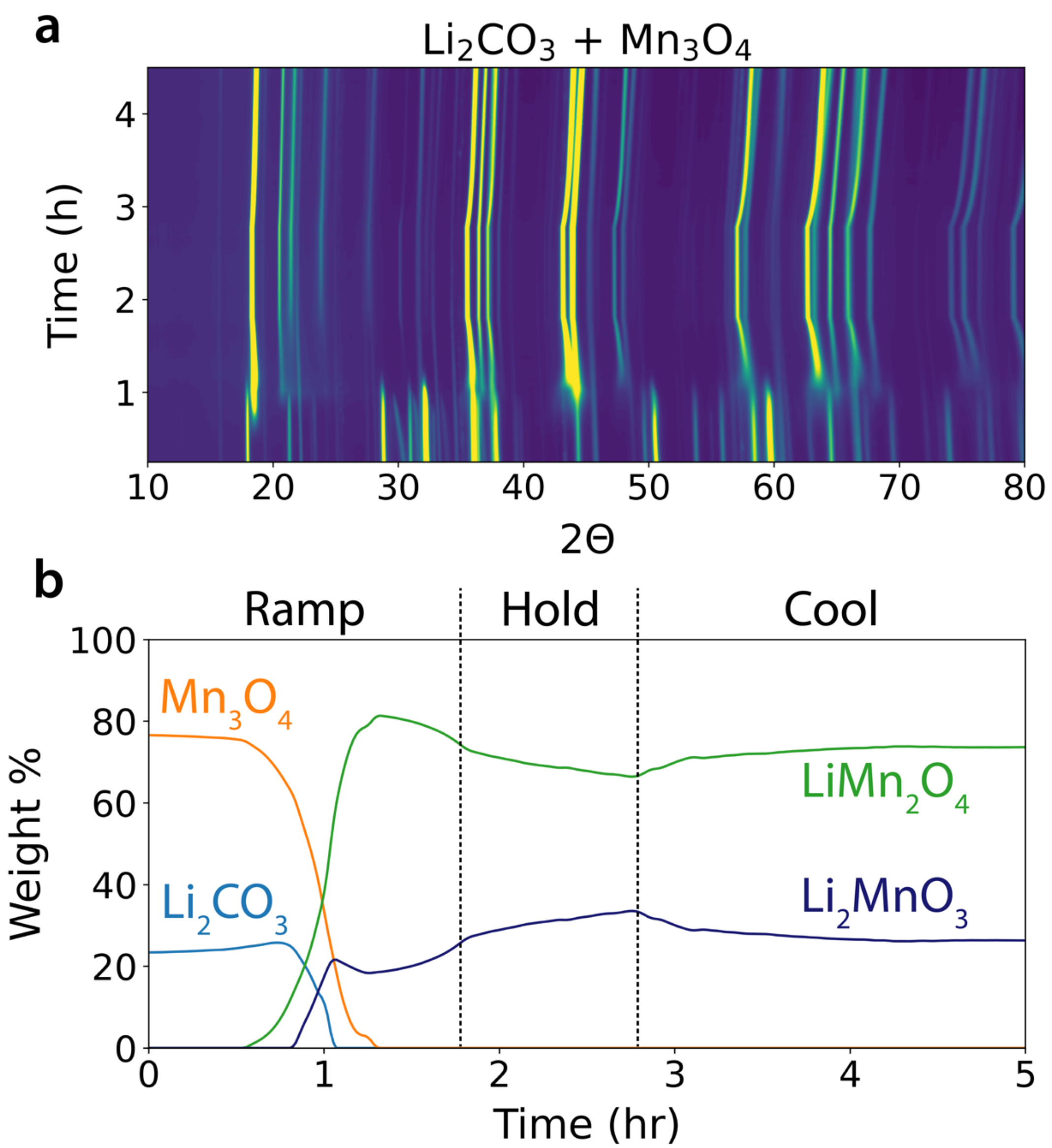

Supplementary Fig. 18 | *In-situ* characterization of the reaction sequence for Li_2CO_3 and Mn_3O_4. (**a**) The heatmap shows the time dependent XRD intensities measured from a sample containing Li_2CO_3 and Mn_3O_4, mixed in a 1:1 Li:Mn ratio. The sample was heated from room temperature to 1000 °C during the ramp, held at 1000 °C for 1 h, then naturally cooled. (**b**) The weight fractions of all phases detected from the XRD measurements are also plotted as a function of time.

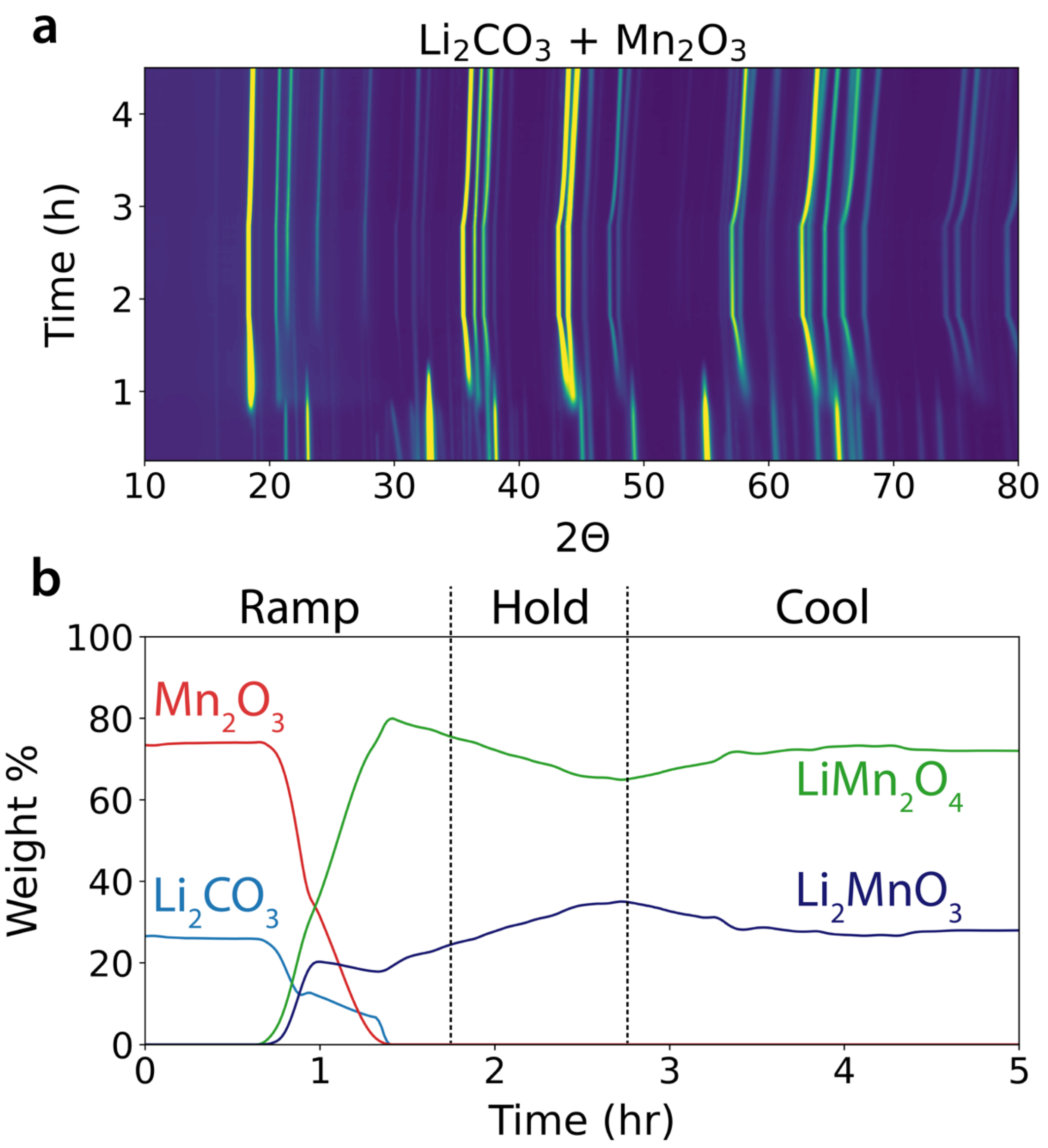

Supplementary Fig. 19 | *In-situ* characterization of the reaction sequence for Li_2CO_3 and Mn_2O_3. (**a**) The heatmap shows the time dependent XRD intensities measured from a sample containing Li_2CO_3 and Mn_2O_3, mixed in a 1:1 Li:Mn ratio. The sample was heated from room temperature to 1000 °C during the ramp, held at 1000 °C for 1 h, then naturally cooled. (**b**) The weight fractions of all phases detected from the XRD measurements are also plotted as a function of time.

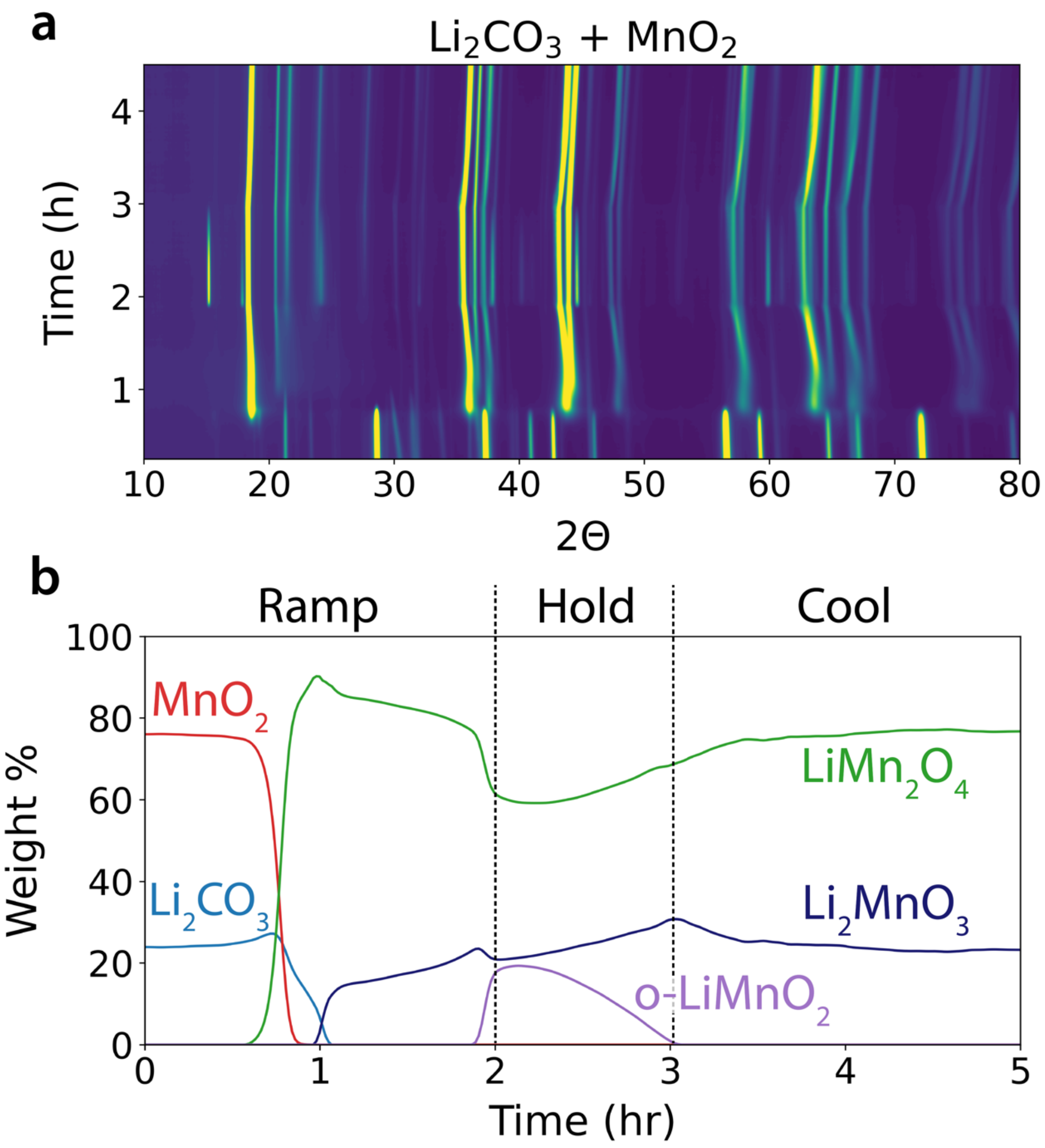

Supplementary Fig. 20 | *In-situ* characterization of the reaction sequence for Li_2CO_3 and MnO_2. (**a**) The heatmap shows the time dependent XRD intensities measured from a sample containing Li_2CO_3 and MnO_2, mixed in a 1:1 ratio of Li to Mn. The sample was heated from room temperature to 1000 °C during the ramp, held at 1000 °C for 1 h, then naturally cooled. (**b**) The weight fractions of all phases detected from the XRD measurements are also plotted as a function of time.

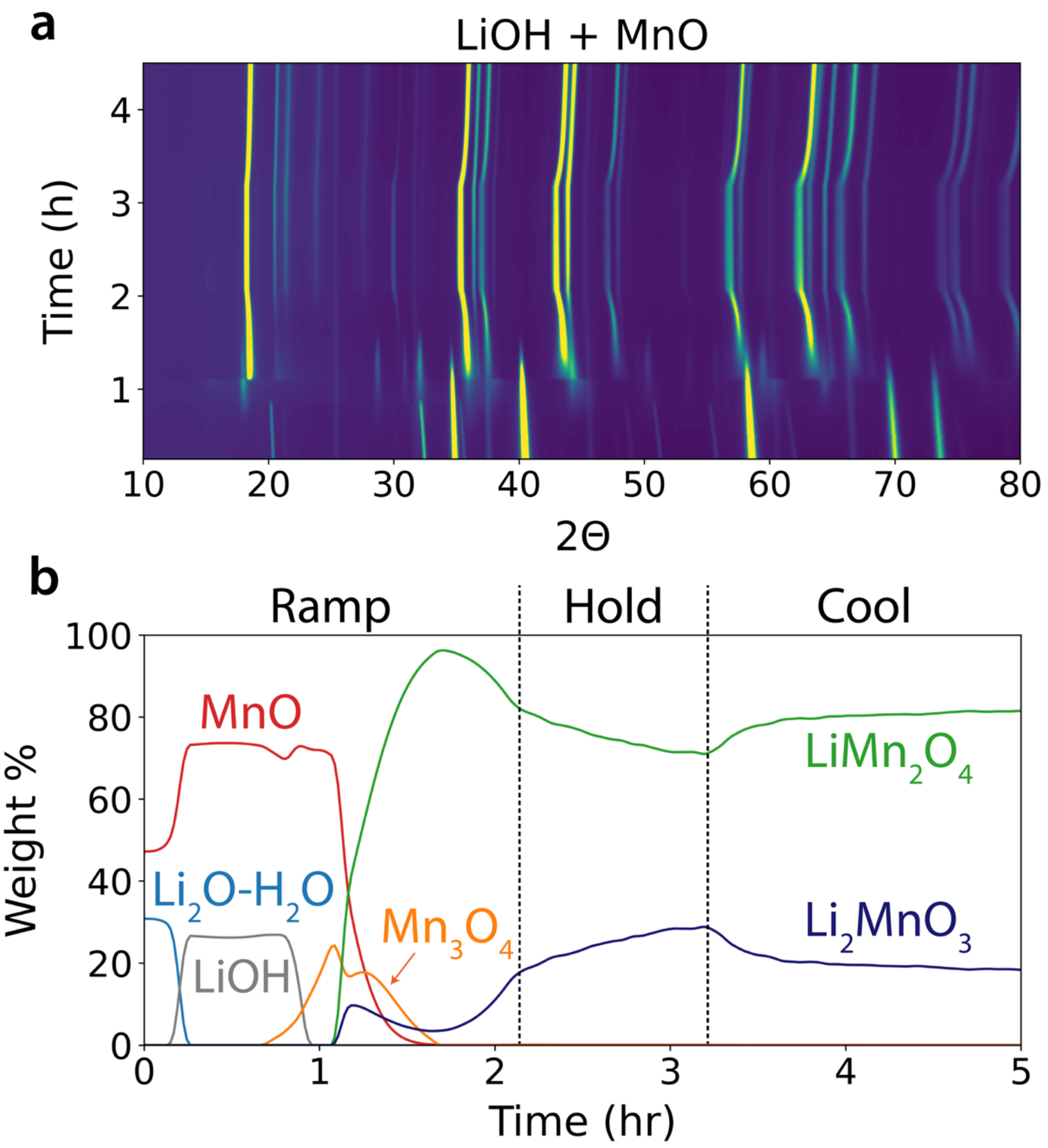

Supplementary Fig. 21 | *In-situ* characterization of the reaction sequence for LiOH and MnO. (**a**) The heatmap shows the time dependent XRD intensities measured from a sample containing LiOH and MnO, mixed in a 1:1 ratio of Li to Mn. The sample was heated from room temperature to 1000 °C during the ramp, held at 1000 °C for 1 h, then naturally cooled. (**b**) The weight fractions of all phases detected from the XRD measurements are also plotted as a function of time.

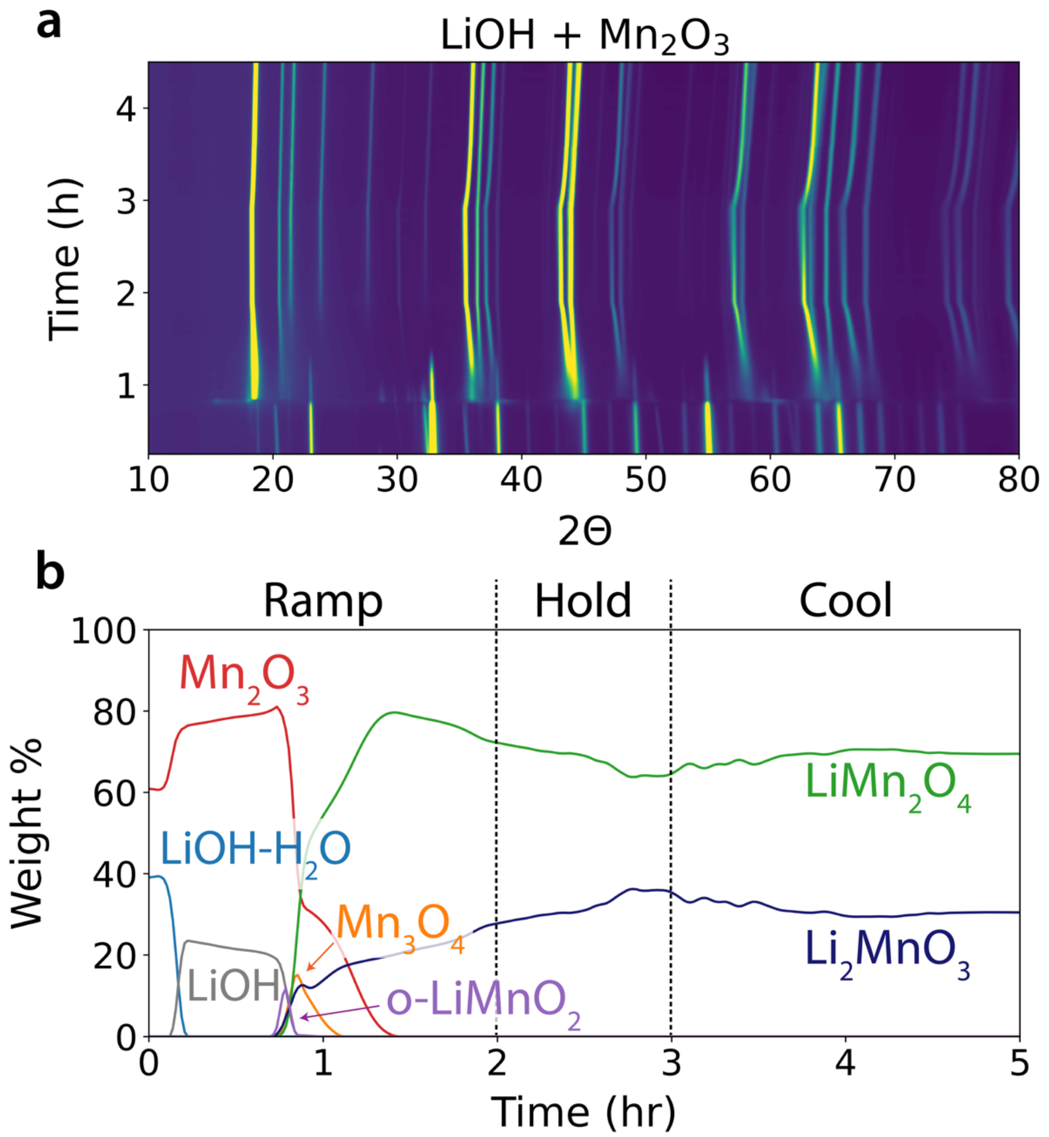

Supplementary Fig. 22 | *In-situ* characterization of the reaction sequence for LiOH and Mn_2O_3. (**a**) The heatmap shows the time dependent XRD intensities measured from a sample containing LiOH and Mn_2O_3, mixed in a 1:1 ratio of Li to Mn. The sample was heated from room temperature to 1000 °C during the ramp, held at 1000 °C for 1 h, then naturally cooled. (**b**) The weight fractions of all phases detected from the XRD measurements are also plotted as a function of time.

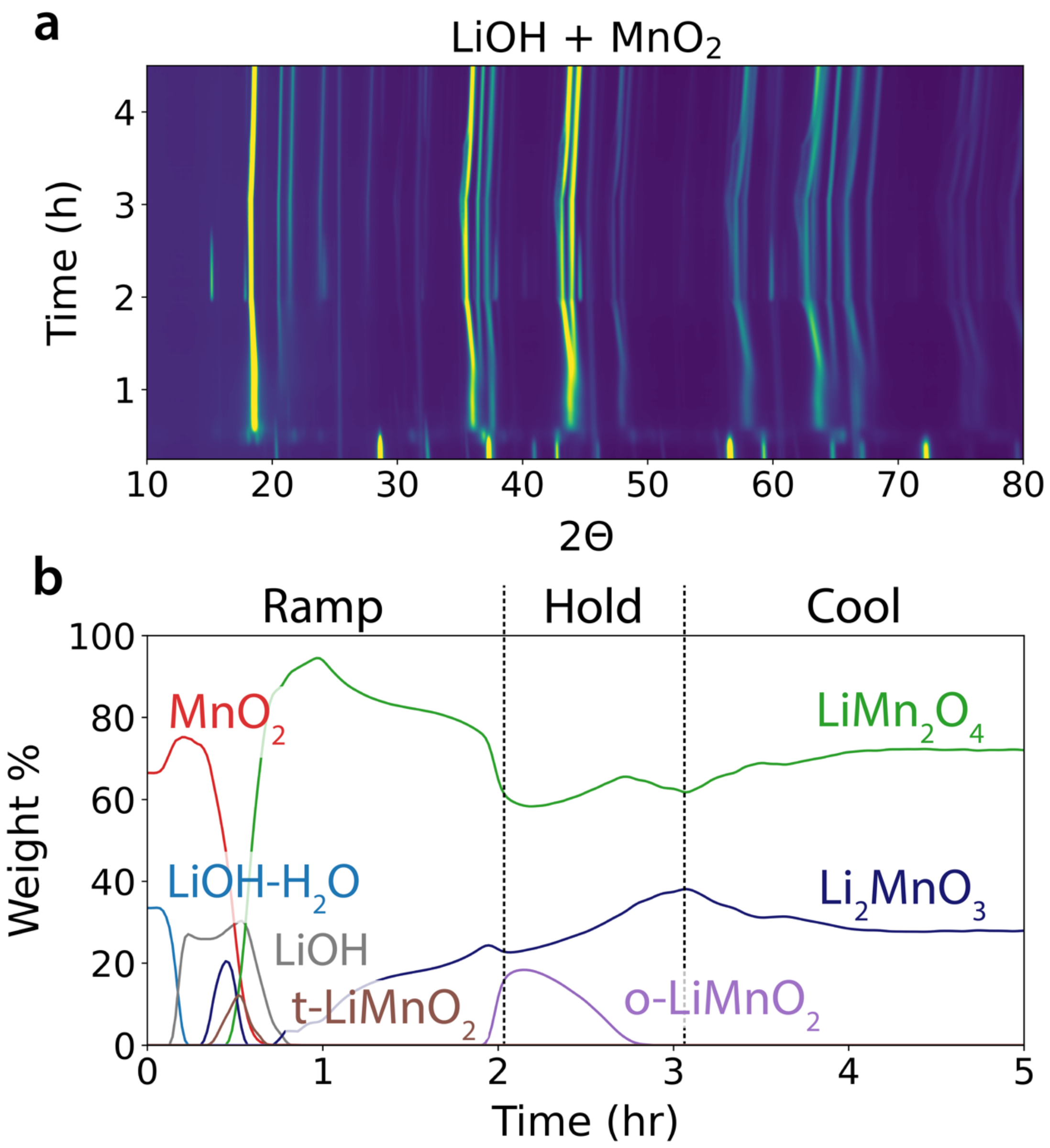

Supplementary Fig. 23 | *In-situ* characterization of the reaction sequence for LiOH and MnO_2. (**a**) The heatmap shows the time dependent XRD intensities measured from a sample containing LiOH and MnO_2, mixed in a 1:1 ratio of Li to Mn. The sample was heated from room temperature to 1000 °C during the ramp, held at 1000 °C for 1 h, then naturally cooled. (**b**) The weight fractions of all phases detected from the XRD measurements are also plotted as a function of time.

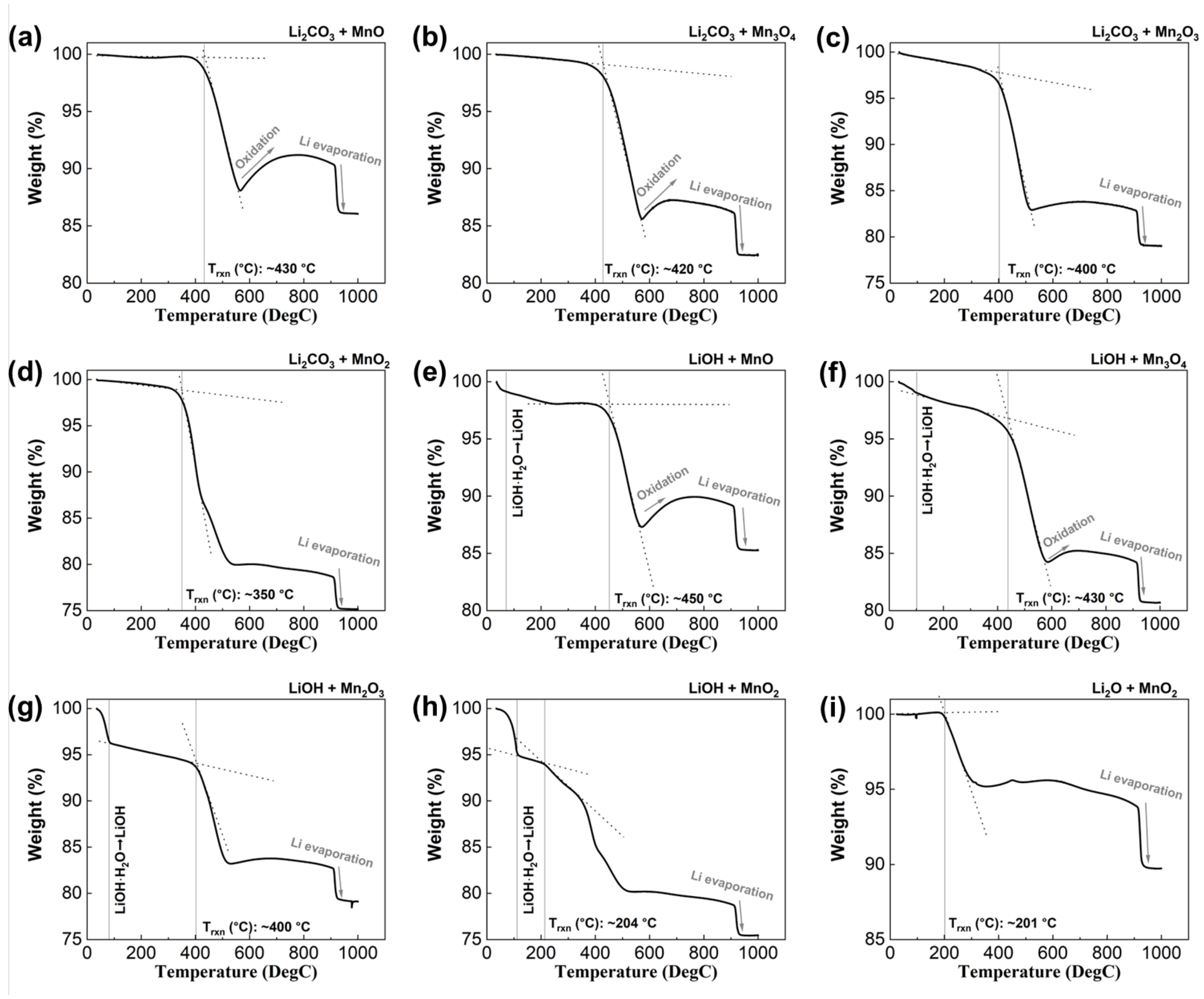

Supplementary Fig. 24 | TGA measurements to detect reaction temperatures in the Li-Mn-O space. Results from thermogravimetric analysis (TGA) measurements are shown for the pairs of Li and Mn reactants included in our final analysis. In each case, we specify the temperature where the starting materials begin to react (T_{rxn}). These are determined based on weight loss associated with the evolution of CO_2 or H_2O from the Li source, or with O_2 loss from the Mn source.

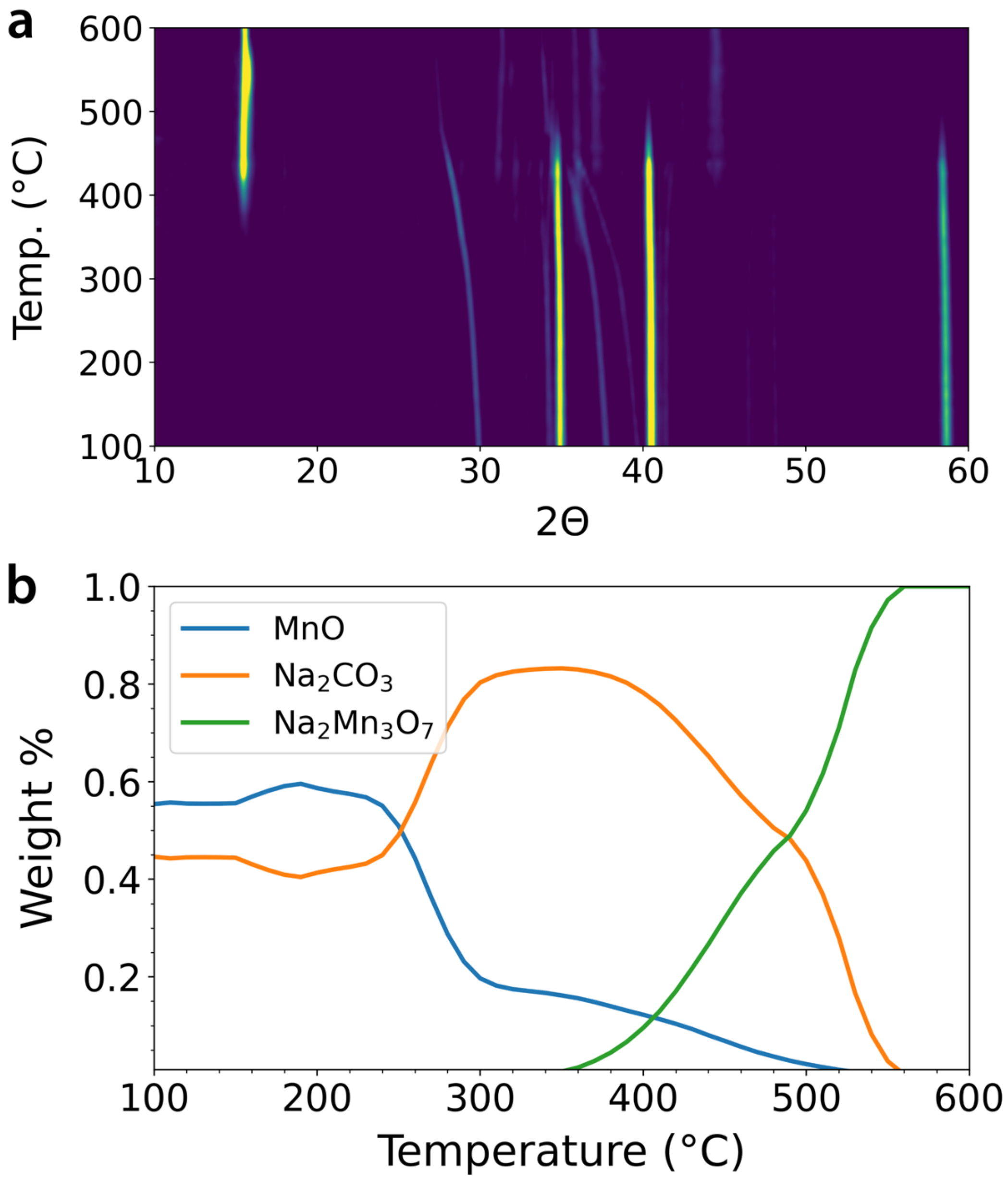

Supplementary Fig. 25 | *In-situ* characterization of the reaction sequence for Na_2CO_3 and MnO. (**a**) The heatmap shows the temperature dependent XRD intensities measured from a sample of Na_2CO_3 and MnO, mixed in a 1:1 ratio of Na to Mn. Heating was performed at a rate of 10 °C/min up to 600 °C, while XRD scans were carried out once every 10 °C. (**b**) The weight fractions of all phases detected from the XRD measurements are also plotted as a function of temperature.

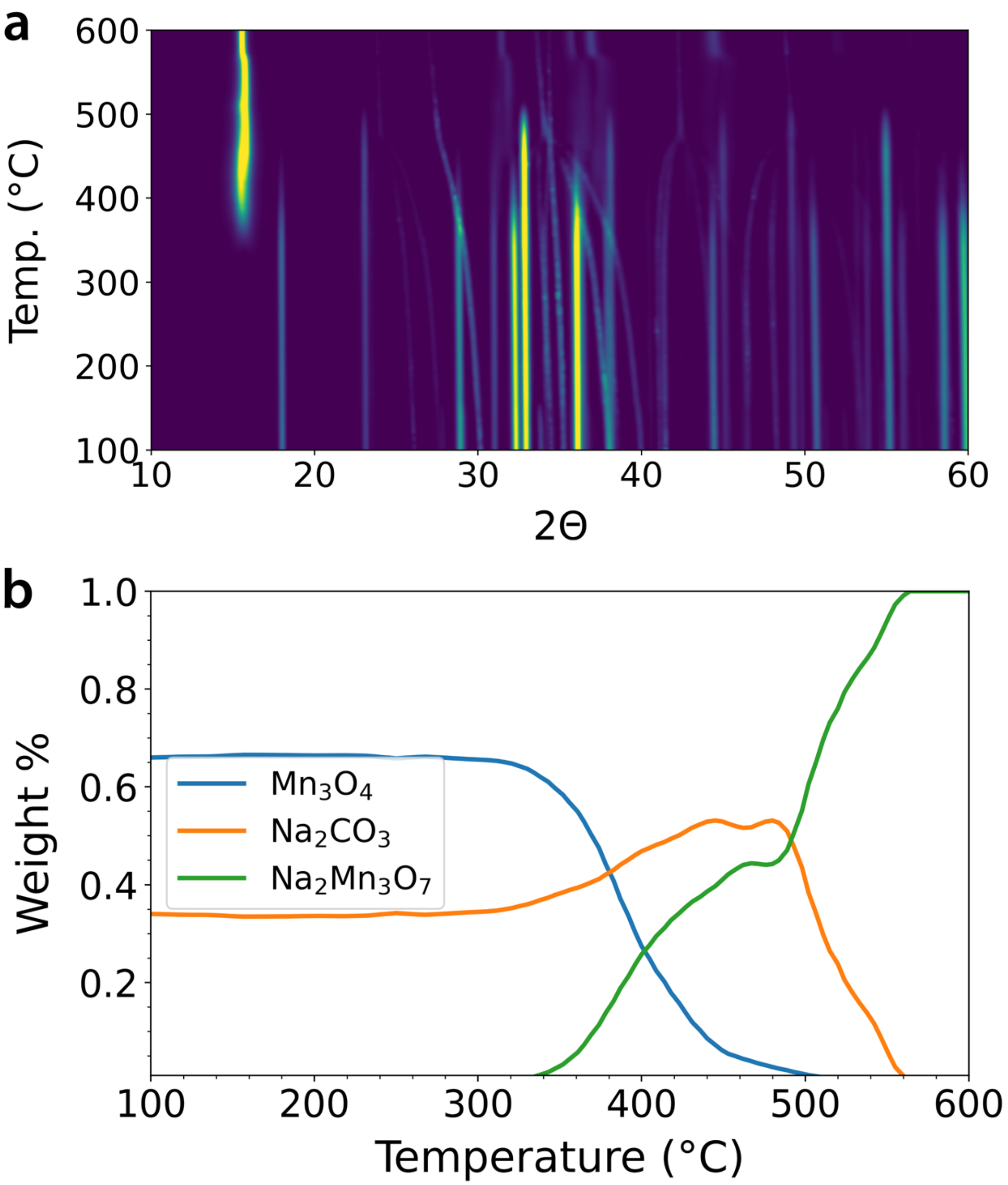

Supplementary Fig. 26 | *In-situ* characterization of the reaction sequence for Na_2CO_3 and Mn_3O_4. (**a**) The heatmap shows the temperature dependent XRD intensities measured from a sample of Na_2CO_3 and Mn_3O_4, mixed in a 1:1 Na:Mn ratio. Heating was performed at a rate of 10 °C/min up to 600 °C, while XRD scans were carried out once every 10 °C. (**b**) The weight fractions of all phases detected from the XRD measurements are also plotted as a function of temperature.

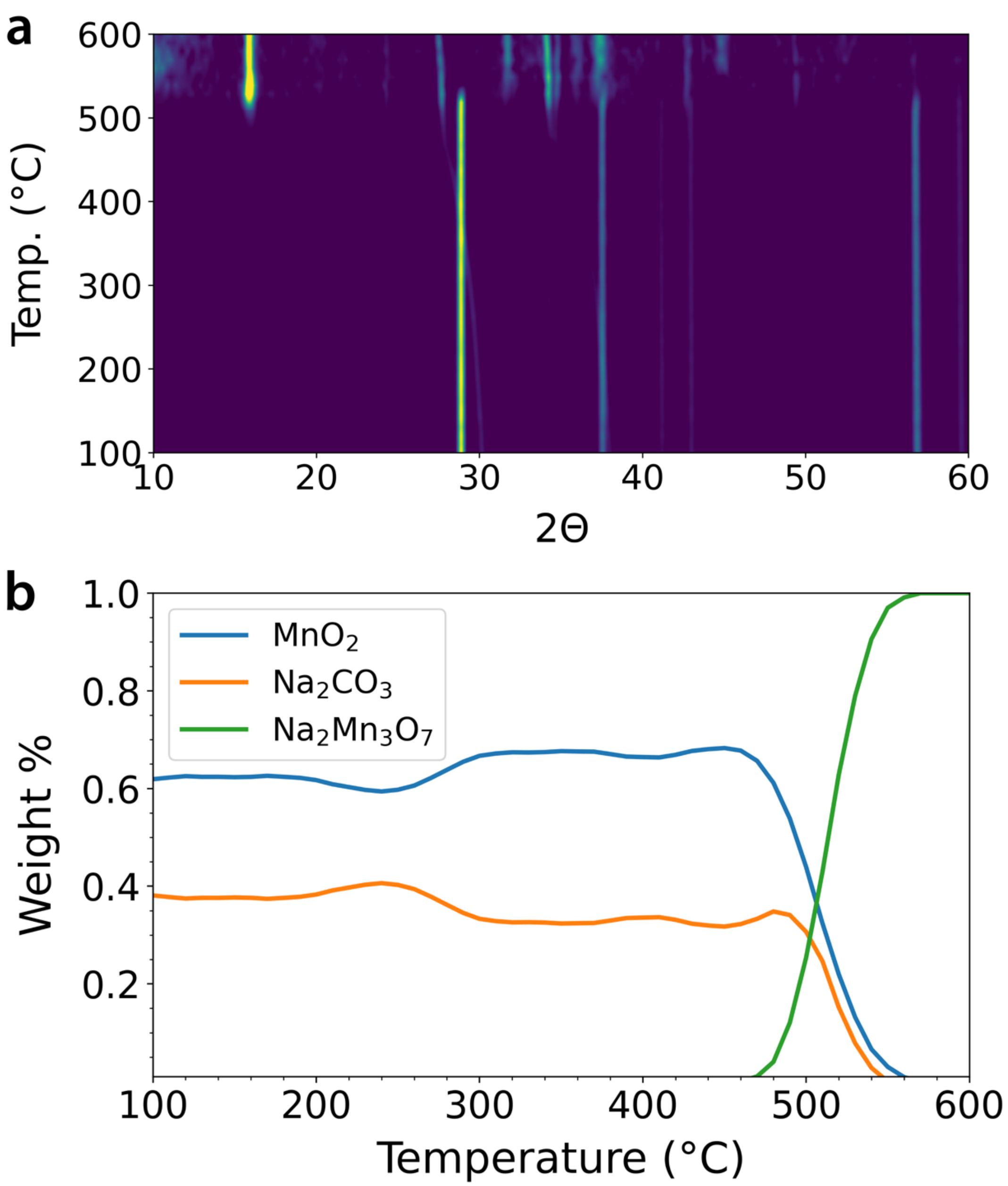

Supplementary Fig. 27 | *In-situ* characterization of the reaction sequence for Na_2CO_3 and MnO_2. (**a**) The heatmap shows the temperature dependent XRD intensities measured from a sample of Na_2CO_3 and MnO_2, mixed in a 1:1 Na:Mn ratio. Heating was performed at a rate of 10 °C/min up to 600 °C, while XRD scans were carried out once every 10 °C. (**b**) The weight fractions of all phases detected from the XRD measurements are also plotted as a function of temperature.

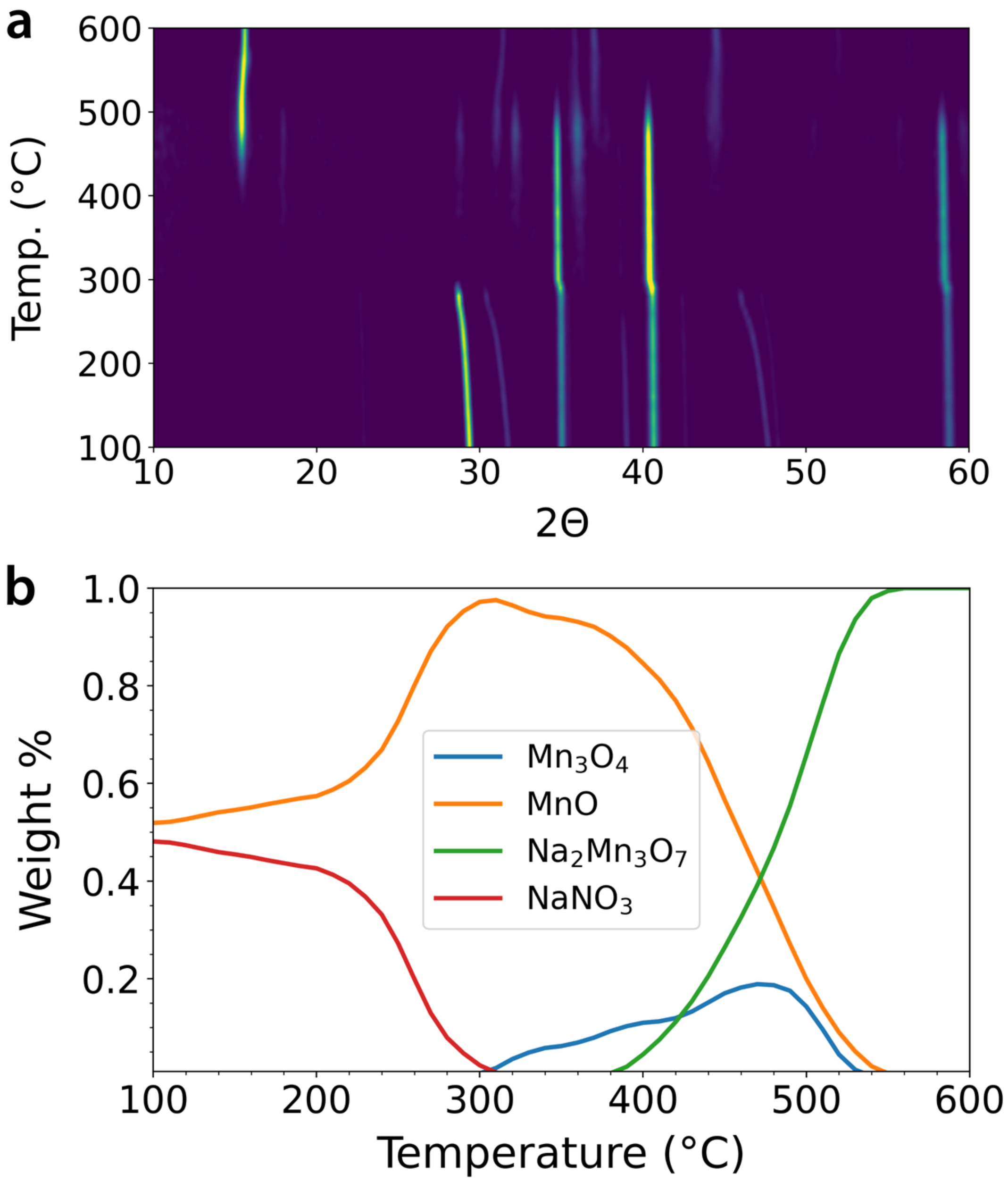

Supplementary Fig. 28 | *In-situ* characterization of the reaction sequence for $NaNO_3$ and MnO. (**a**) The heatmap shows the temperature dependent XRD intensities measured from a sample of $NaNO_3$ and MnO, mixed in a 1:1 ratio of Na to Mn. Heating was performed at a rate of 10 °C/min up to 600 °C, while XRD scans were carried out once every 10 °C. (**b**) The weight fractions of all phases detected from the XRD measurements are also plotted as a function of temperature.

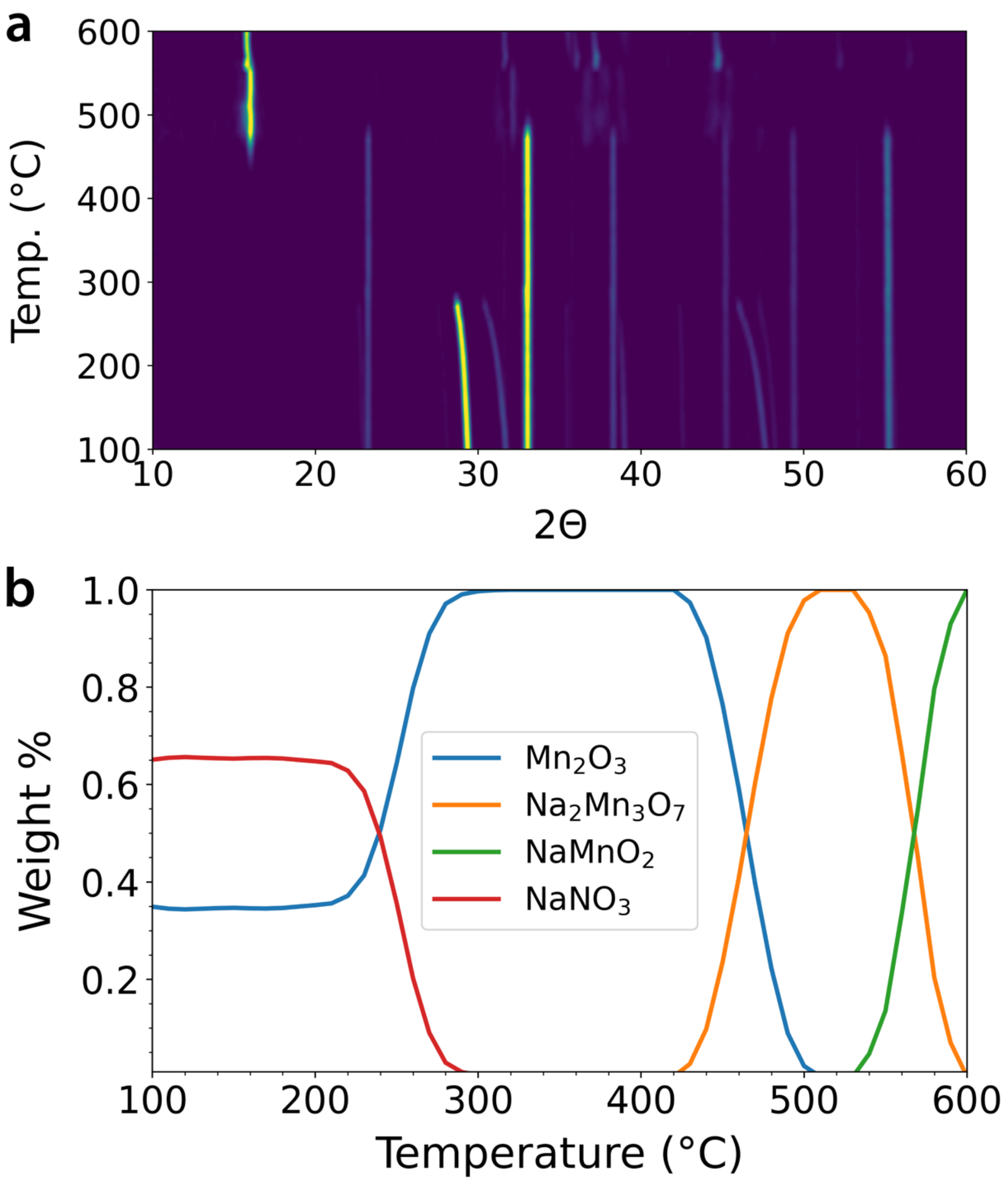

Supplementary Fig. 29 | *In-situ* characterization of the reaction sequence for $NaNO_3$ and Mn_2O_3. (**a**) The heatmap shows the temperature dependent XRD intensities measured from a sample of $NaNO_3$ and Mn_2O_3, mixed in a 1:1 Na:Mn ratio. Heating was performed at a rate of 10 °C/min up to 600 °C, while XRD scans were carried out once every 10 °C. (**b**) The weight fractions of all phases detected from the XRD measurements are also plotted as a function of temperature.

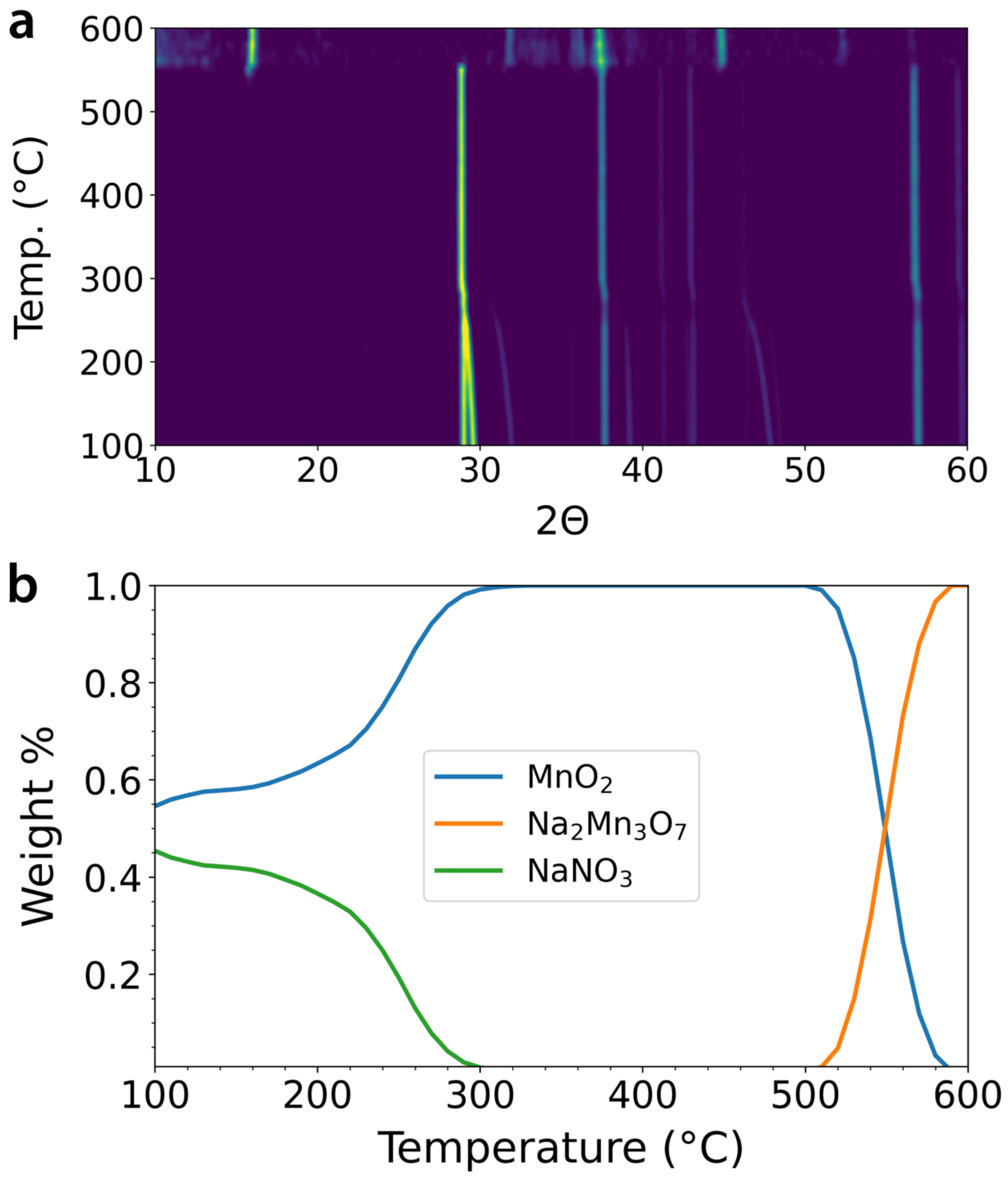

Supplementary Fig. 30 | *In-situ* characterization of the reaction sequence for $NaNO_3$ and MnO_2. (**a**) The heatmap shows the temperature dependent XRD intensities measured from a sample of $NaNO_3$ and MnO_2, mixed in a 1:1 ratio of Na to Mn. Heating was performed at a rate of 10 °C/min up to 600 °C, while XRD scans were carried out once every 10 °C. (**b**) The weight fractions of all phases detected from the XRD measurements are also plotted as a function of temperature.

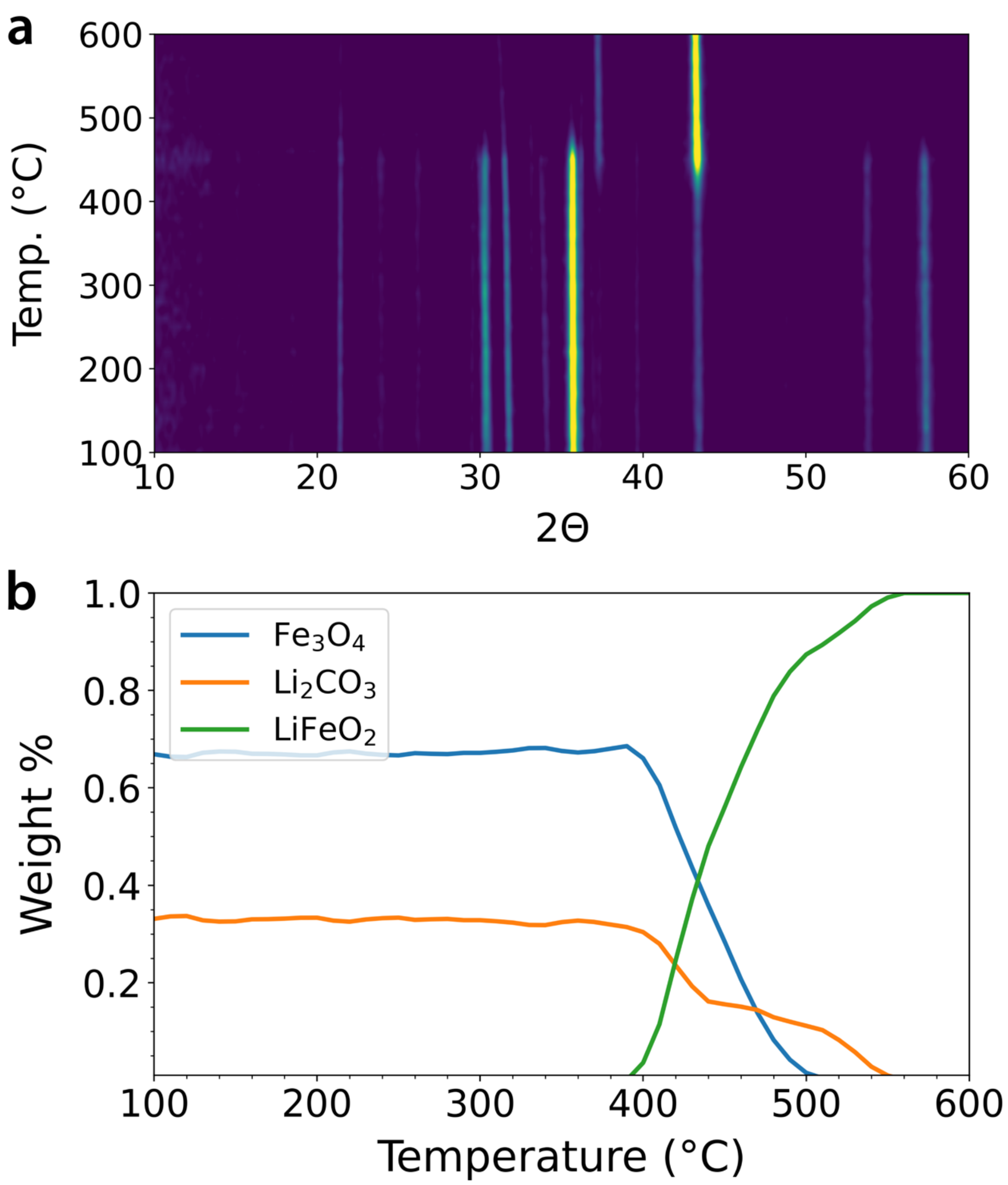

Supplementary Fig. 31 | *In-situ* characterization of the reaction sequence for Li_2CO_3 and Fe_3O_4. (**a**) The heatmap shows the temperature dependent XRD intensities measured from a sample of Li_2CO_3 and Fe_3O_4, mixed in a 1:1 Li:Fe ratio. Heating was performed at a rate of 10 °C/min up to 600 °C, while XRD scans were carried out once every 10 °C. (**b**) The weight fractions of all phases detected from the XRD measurements are also plotted as a function of temperature.

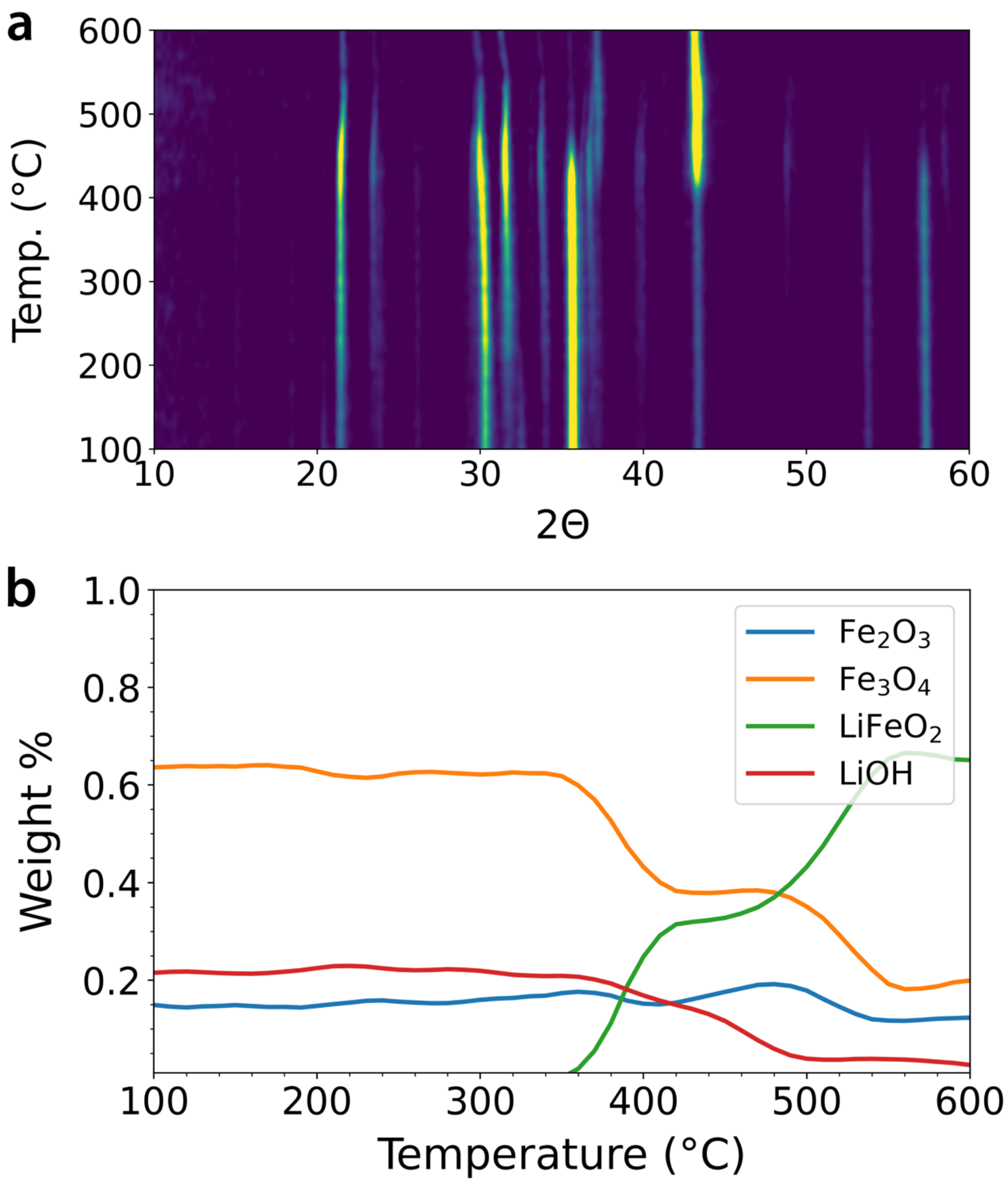

Supplementary Fig. 32 | *In-situ* characterization of the reaction sequence for LiOH and Fe_3O_4. (**a**) The heatmap shows the temperature dependent XRD intensities measured from a sample of LiOH and Fe_3O_4, mixed in a 1:1 ratio of Li to Fe. Heating was performed at a rate of 10 °C/min up to 600 °C, while XRD scans were carried out once every 10 °C. (**b**) The weight fractions of all phases detected from the XRD measurements are also plotted as a function of temperature.

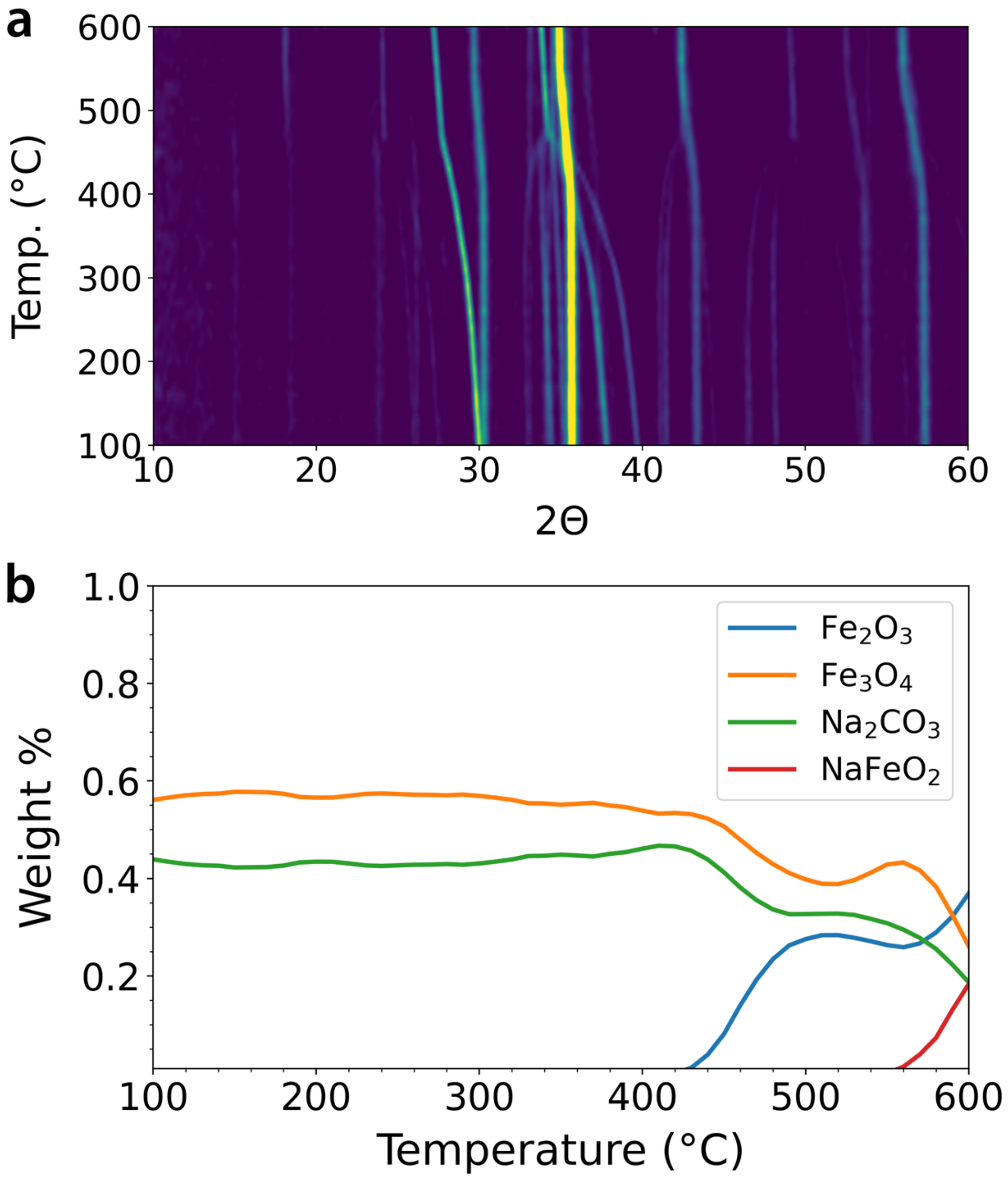

Supplementary Fig. 33 | *In-situ* characterization of the reaction sequence for Na_2CO_3 and Fe_3O_4. (**a**) The heatmap shows the temperature dependent XRD intensities measured from a sample of Na_2CO_3 and Fe_3O_4, mixed in a 1:1 Na:Fe ratio. Heating was performed at a rate of 10 °C/min up to 600 °C, while XRD scans were carried out once every 10 °C. (**b**) The weight fractions of all phases detected from the XRD measurements are also plotted as a function of temperature.

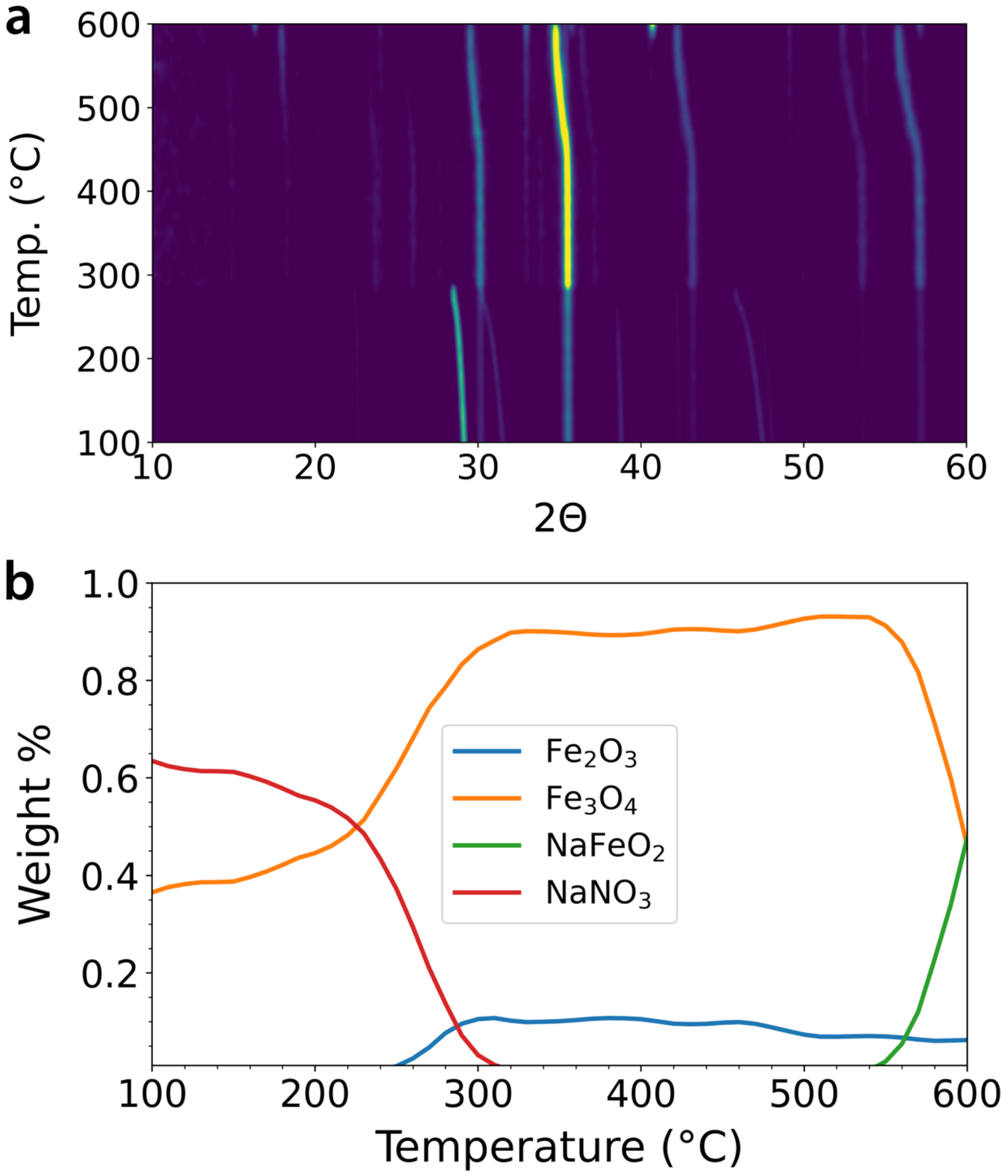

Supplementary Fig. 34 | *In-situ* characterization of the reaction sequence for $NaNO_3$ and Fe_3O_4. (**a**) The heatmap shows the temperature dependent XRD intensities measured from a sample of $NaNO_3$ and Fe_3O_4, mixed in a 1:1 ratio of Na to Fe. Heating was performed at a rate of 10 °C/min up to 600 °C, while XRD scans were carried out once every 10 °C. (**b**) The weight fractions of all phases detected from the XRD measurements are also plotted as a function of temperature.

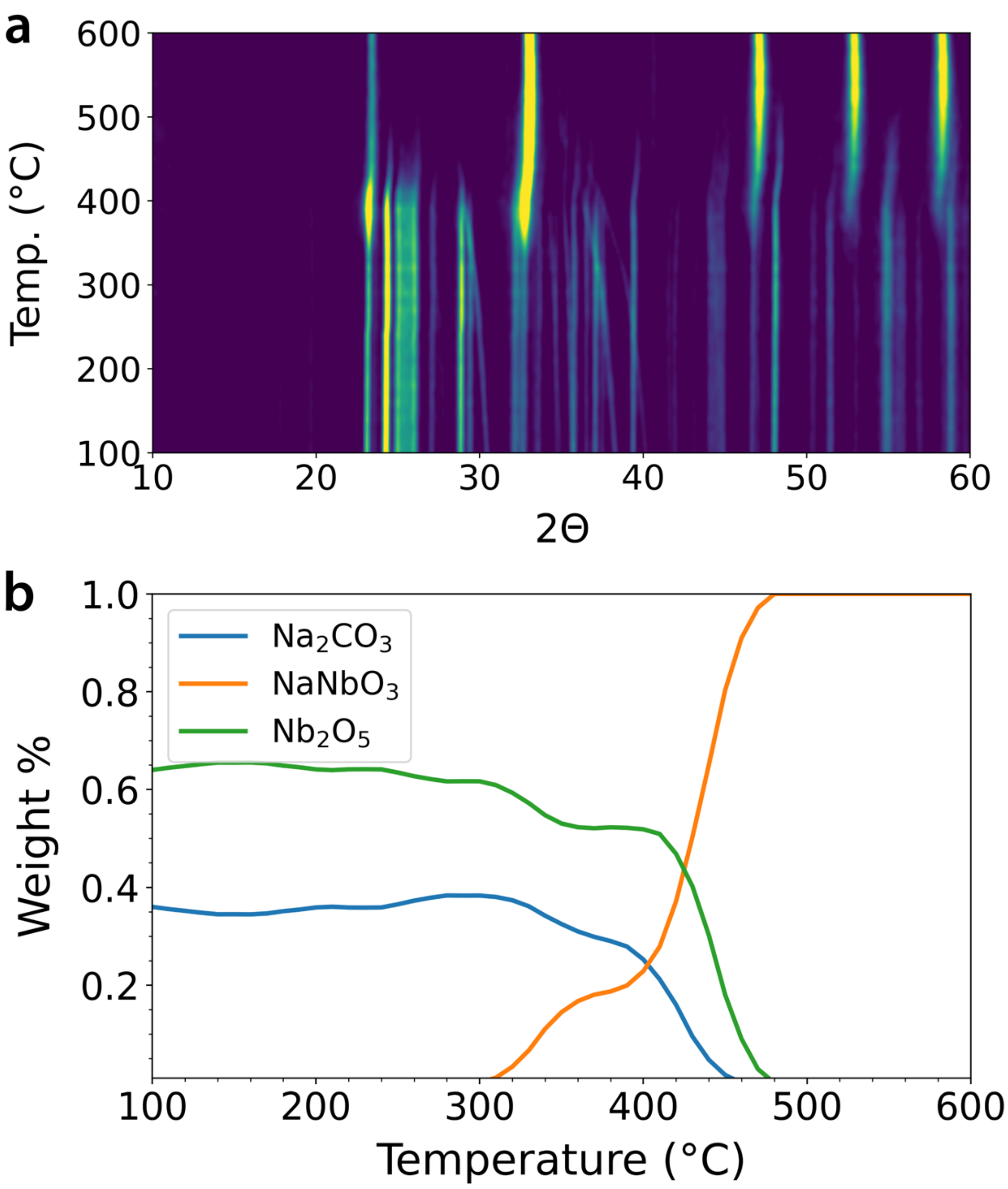

Supplementary Fig. 35 | *In-situ* characterization of the reaction sequence for Na_2CO_3 and Nb_2O_5. (**a**) The heatmap shows the temperature dependent XRD intensities measured from a sample of Na_2CO_3 and Nb_2O_5, mixed in a 1:1 Na:Nb ratio. Heating was performed at a rate of 10 °C/min up to 600 °C, while XRD scans were carried out once every 10 °C. (**b**) The weight fractions of all phases detected from the XRD measurements are also plotted as a function of temperature.

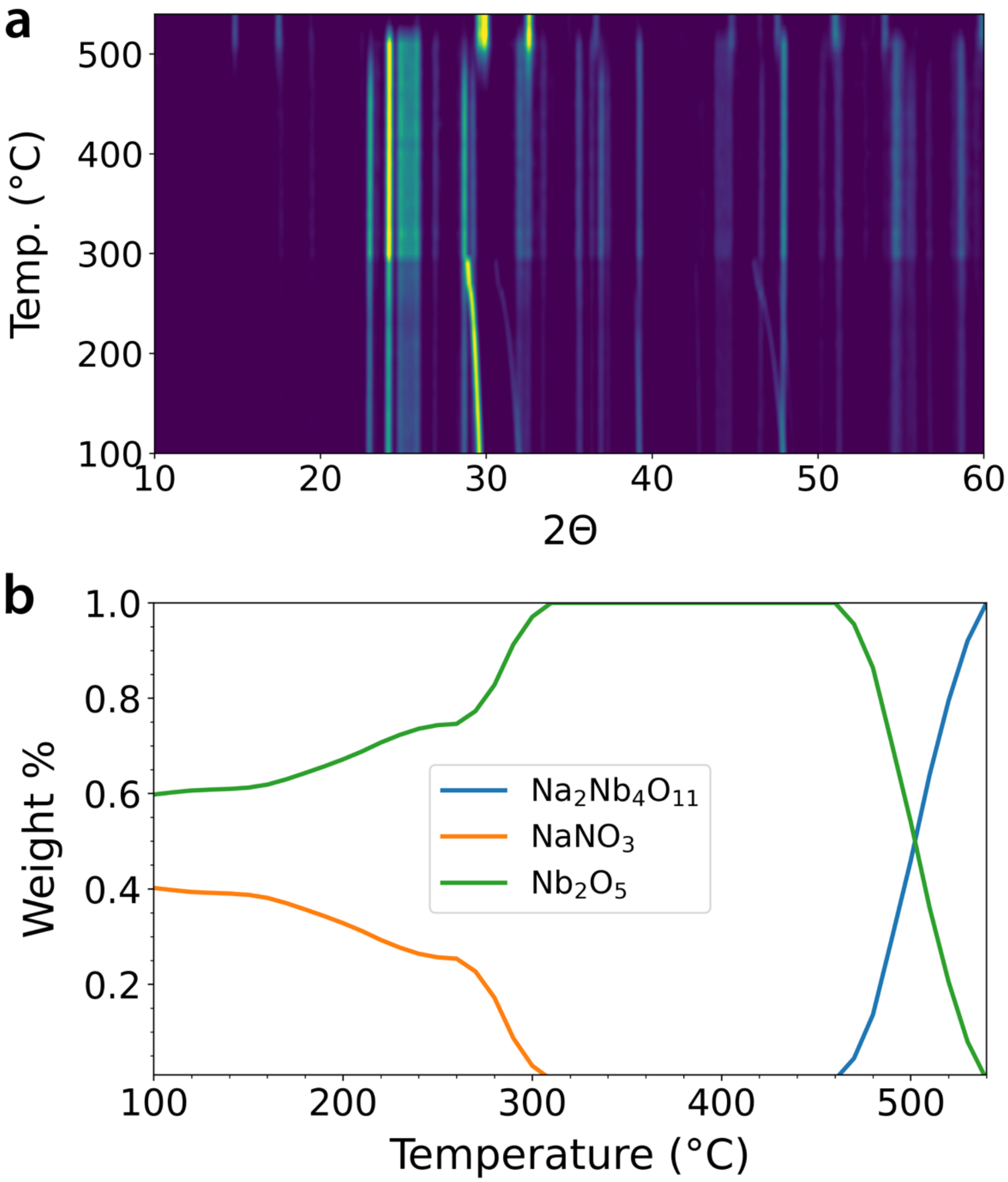

Supplementary Fig. 36 | *In-situ* characterization of the reaction sequence for $NaNO_3$ and Nb_2O_5. (**a**) The heatmap shows the temperature dependent XRD intensities measured from a sample of $NaNO_3$ and Nb_2O_5, mixed in a 1:1 ratio of Na to Nb. Heating was performed at a rate of 10 °C/min up to 540 °C, while XRD scans were carried out once every 10 °C. (**b**) The weight fractions of all phases detected from the XRD measurements are also plotted as a function of temperature.

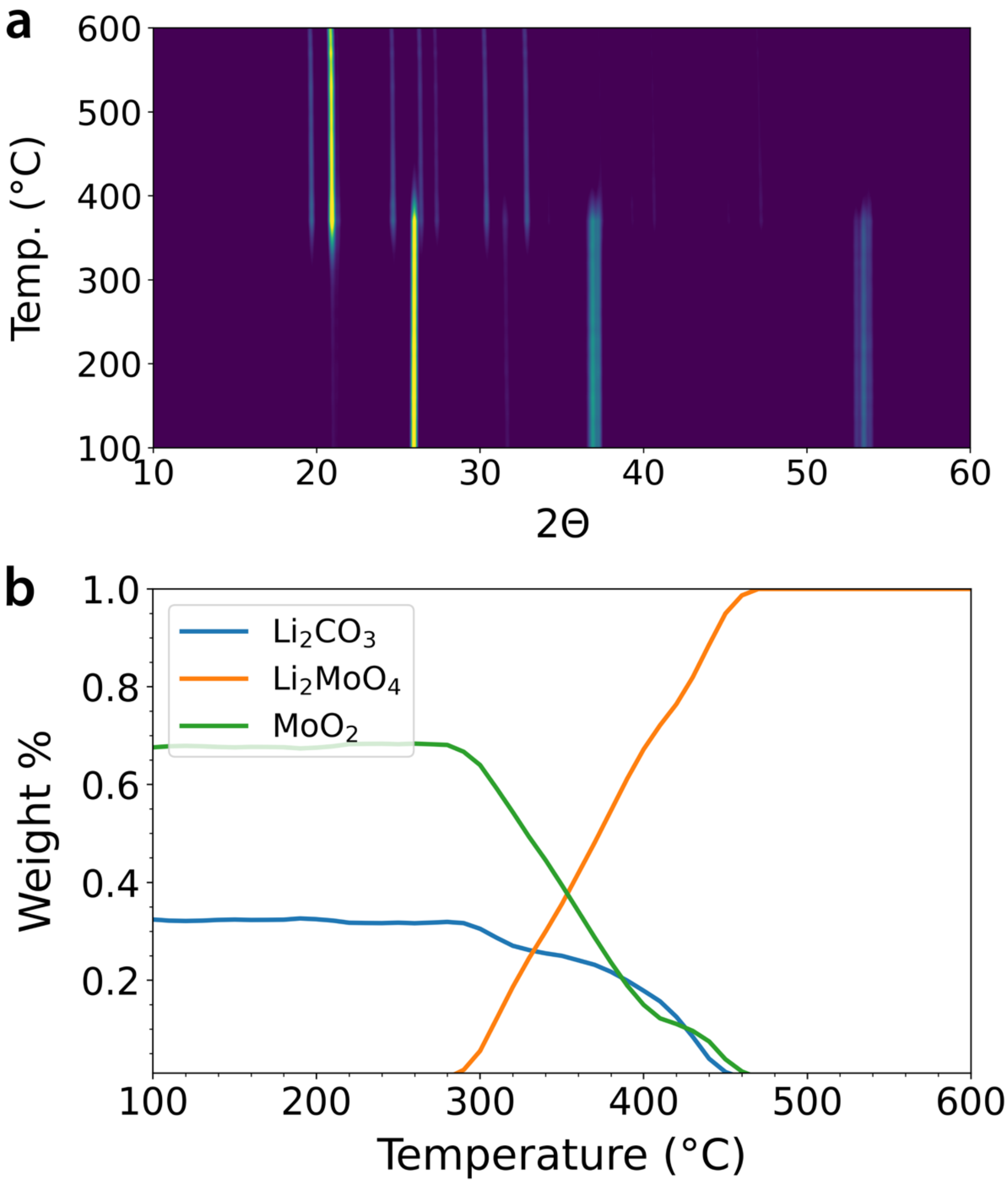

Supplementary Fig. 37 | *In-situ* characterization of the reaction sequence for Li_2CO_3 and MoO_2. (**a**) The heatmap shows the temperature dependent XRD intensities measured from a sample of Li_2CO_3 and MoO_2, mixed in a 1:1 ratio of Li to Mo. Heating was performed at a rate of 10 °C/min up to 600 °C, while XRD scans were carried out once every 10 °C. (**b**) The weight fractions of all phases detected from the XRD measurements are also plotted as a function of temperature.

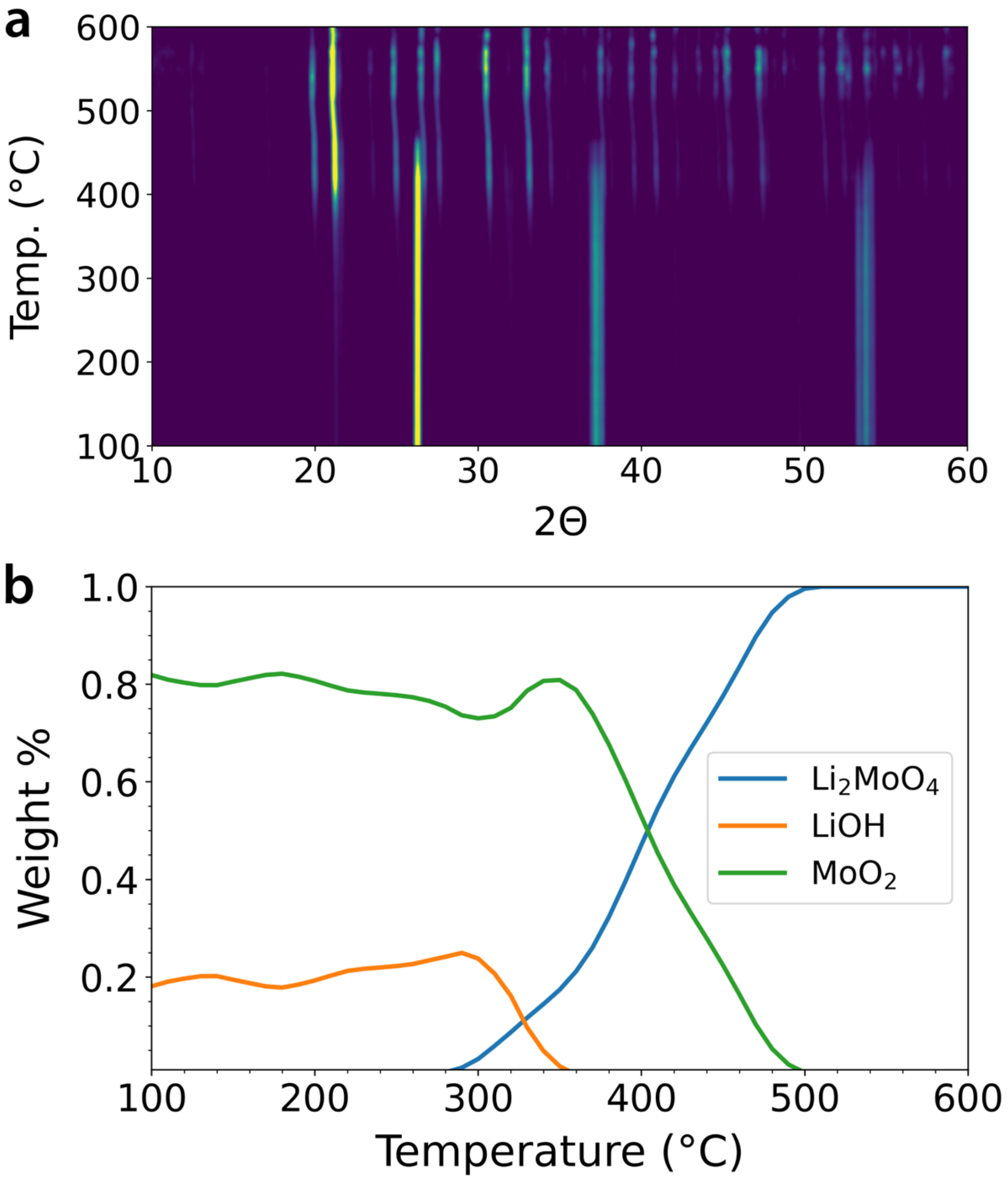

Supplementary Fig. 38 | *In-situ* characterization of the reaction sequence for LiOH and MoO_2. (**a**) The heatmap shows the temperature dependent XRD intensities measured from a sample of LiOH and MoO_2, mixed in a 1:1 ratio of Li to Mo. Heating was performed at a rate of 10 °C/min up to 600 °C, while XRD scans were carried out once every 10 °C. (**b**) The weight fractions of all phases detected from the XRD measurements are also plotted as a function of temperature.

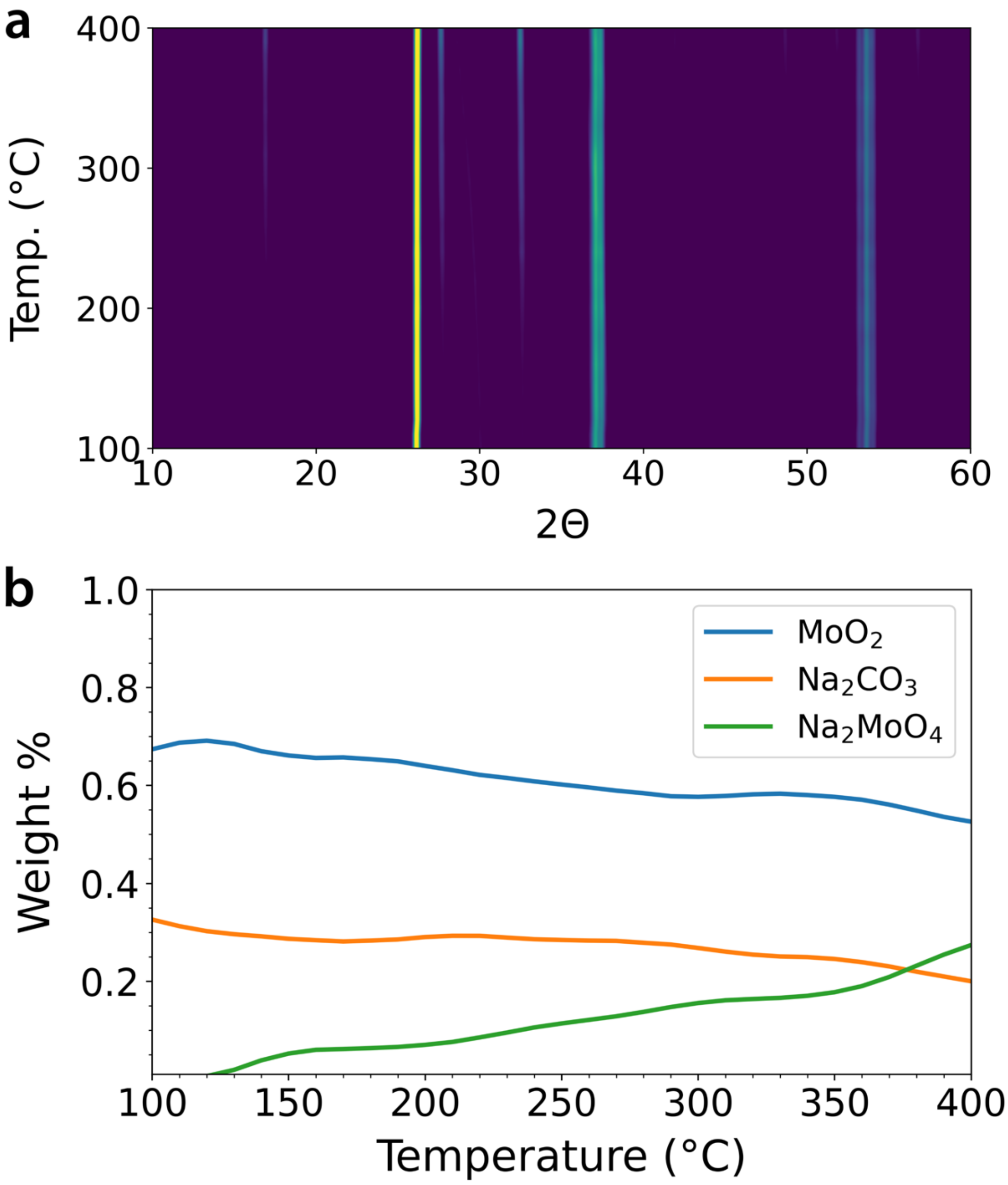

Supplementary Fig. 39 | *In-situ* characterization of the reaction sequence for Na_2CO_3 and MoO_2. (**a**) The heatmap shows the temperature dependent XRD intensities measured from a sample of Na_2CO_3 and MoO_2, mixed in a 1:1 Na:Mo ratio. Heating was performed at a rate of 10 °C/min up to 400 °C, while XRD scans were carried out once every 10 °C. (**b**) The weight fractions of all phases detected from the XRD measurements are also plotted as a function of temperature.

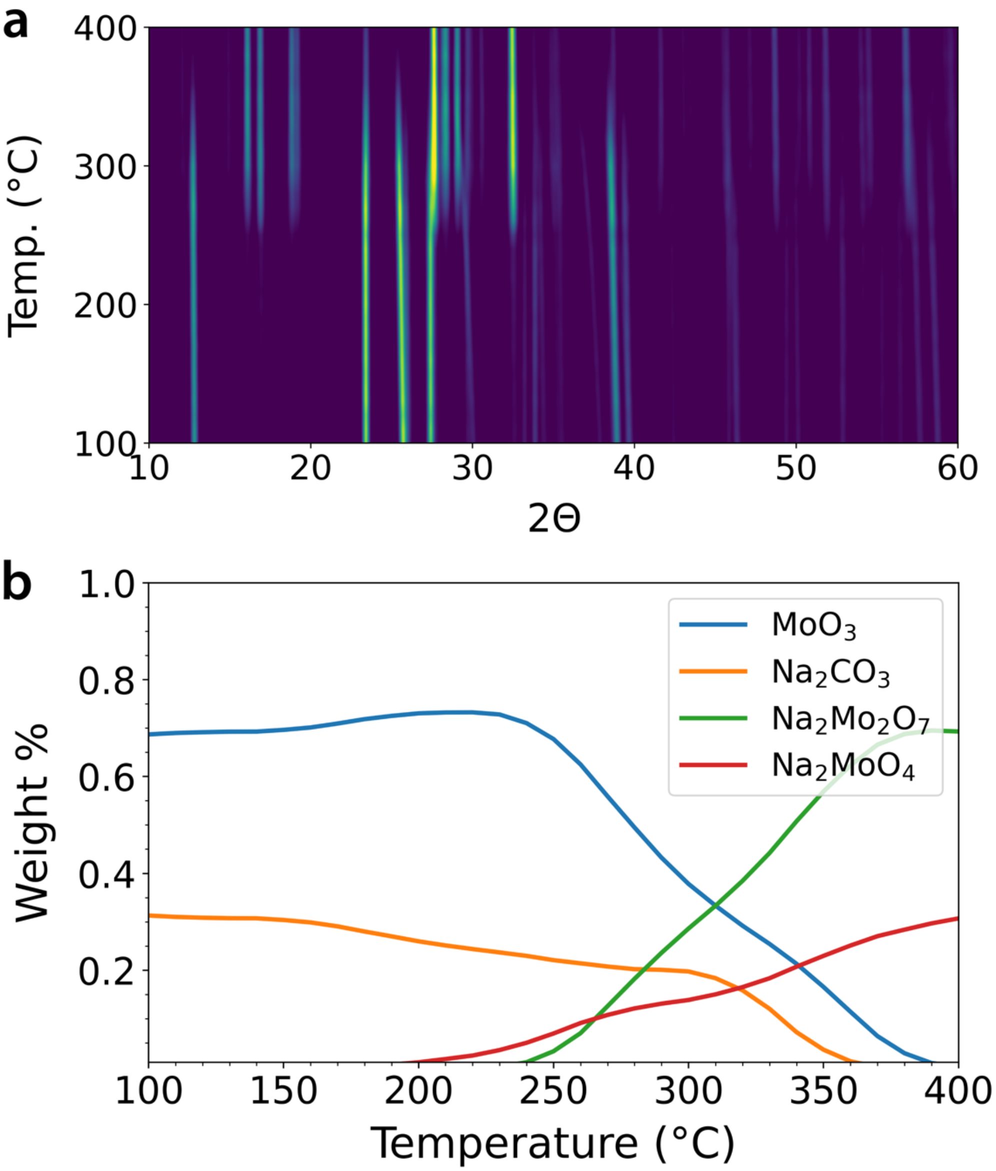

Supplementary Fig. 40 | *In-situ* characterization of the reaction sequence for Na_2CO_3 and MoO_3. (**a**) The heatmap shows the temperature dependent XRD intensities measured from a sample of Na_2CO_3 and MoO_3, mixed in a 1:1 Na:Mo ratio. Heating was performed at a rate of 10 °C/min up to 400 °C, while XRD scans were carried out once every 10 °C. (**b**) The weight fractions of all phases detected from the XRD measurements are also plotted as a function of temperature.

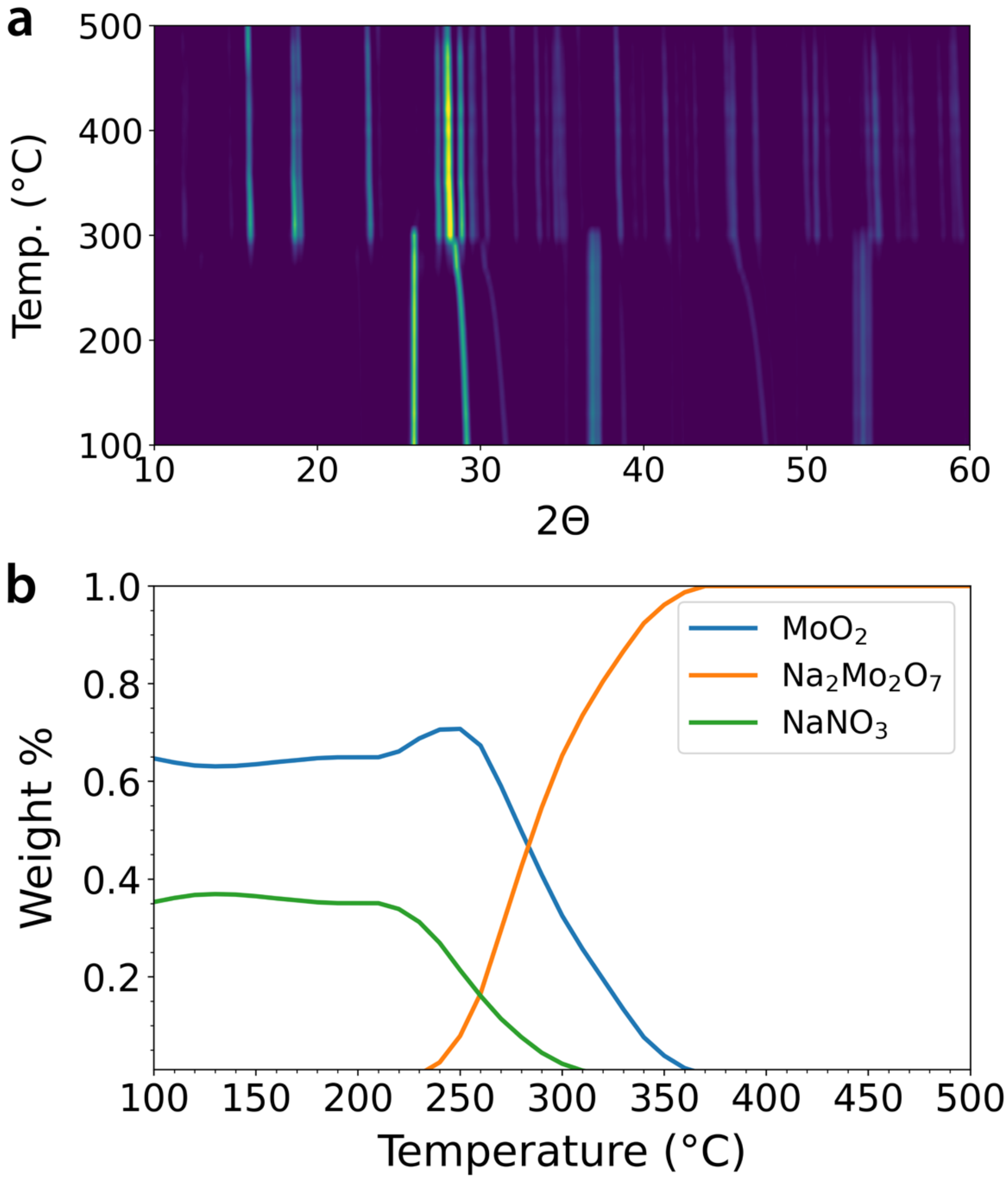

Supplementary Fig. 41 | *In-situ* characterization of the reaction sequence for $NaNO_3$ and MoO_2. (**a**) The heatmap shows the temperature dependent XRD intensities measured from a sample of $NaNO_3$ and MoO_2, mixed in a 1:1 Na:Mo ratio. Heating was performed at a rate of 10 °C/min up to 500 °C, while XRD scans were carried out once every 10 °C. (**b**) The weight fractions of all phases detected from the XRD measurements are also plotted as a function of temperature.

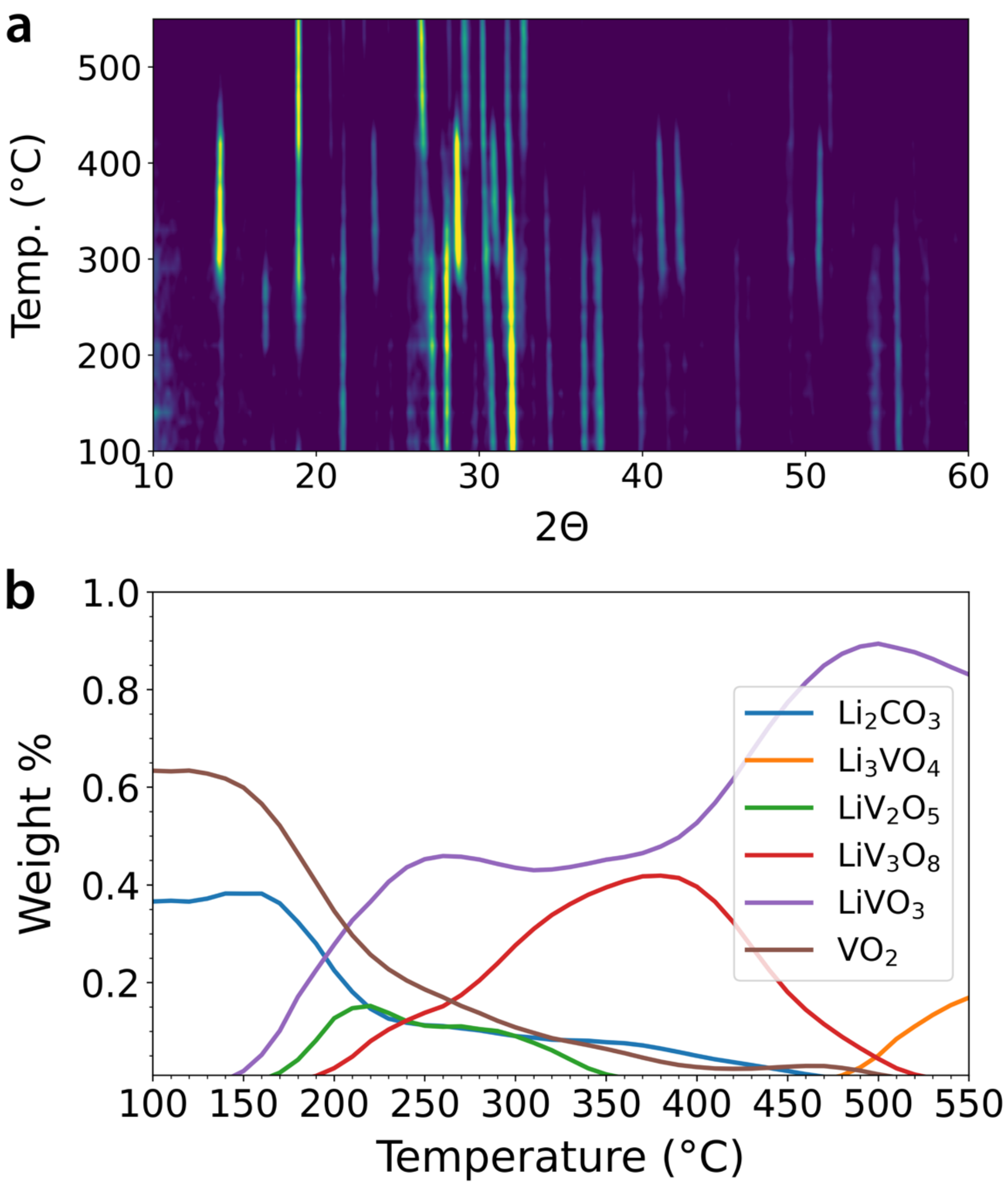

Supplementary Fig. 42 | *In-situ* characterization of the reaction sequence for Li_2CO_3 and VO_2. (**a**) The heatmap shows the temperature dependent XRD intensities measured from a sample of Li_2CO_3 and VO_2, mixed in a 1:1 ratio of Li to V. Heating was performed at a rate of 10 °C/min up to 550 °C, while XRD scans were carried out once every 10 °C. (**b**) The weight fractions of all phases detected from the XRD measurements are also plotted as a function of temperature.

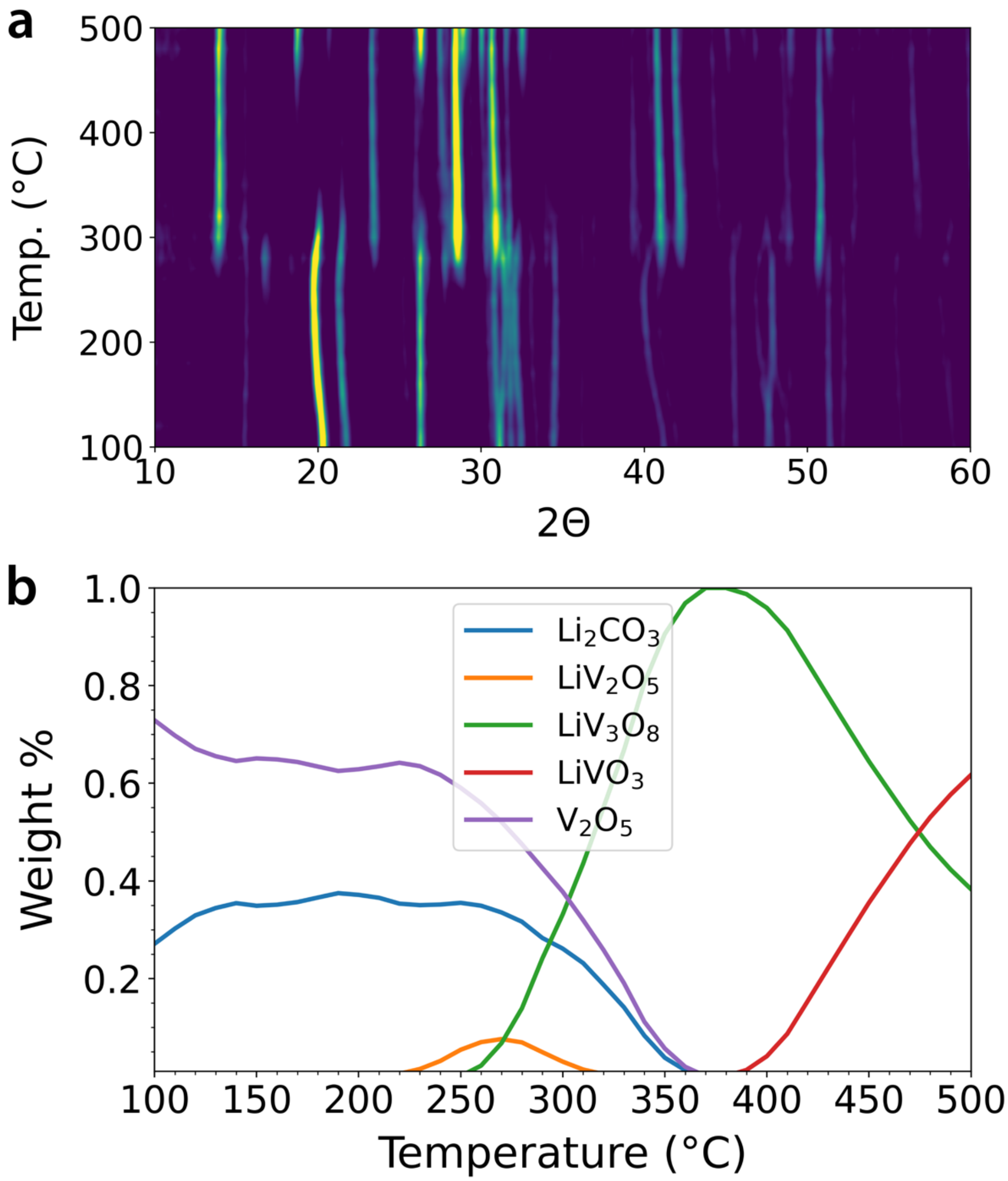

Supplementary Fig. 43 | *In-situ* characterization of the reaction sequence for Li_2CO_3 and V_2O_5. (**a**) The heatmap shows the temperature dependent XRD intensities measured from a sample of Li_2CO_3 and V_2O_5, mixed in a 1:1 ratio of Li to V. Heating was performed at a rate of 10 °C/min up to 500 °C, while XRD scans were carried out once every 10 °C. (**b**) The weight fractions of all phases detected from the XRD measurements are also plotted as a function of temperature.

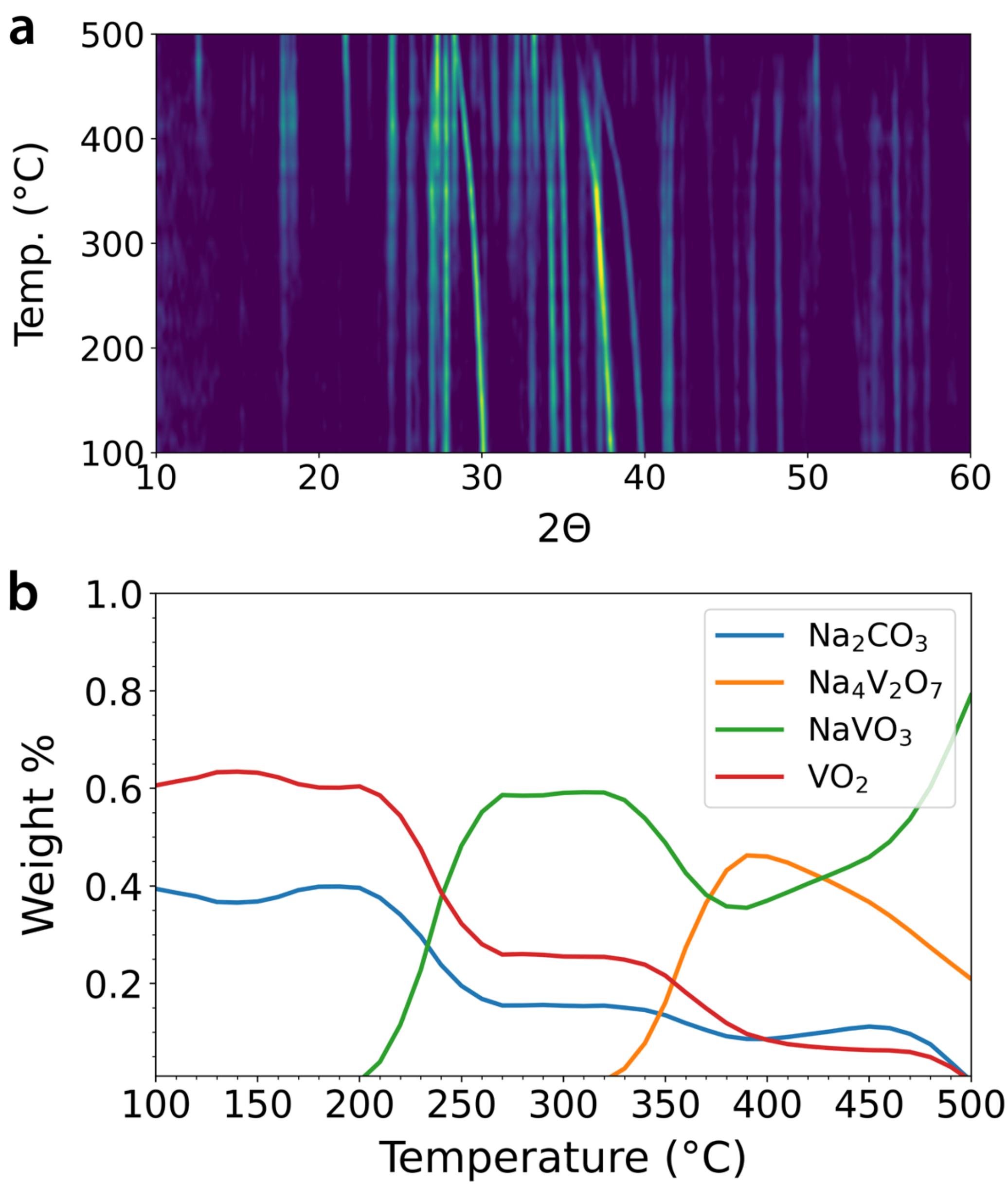

Supplementary Fig. 44 | *In-situ* characterization of the reaction sequence for Na_2CO_3 and VO_2. (**a**) The heatmap shows the temperature dependent XRD intensities measured from a sample of Na_2CO_3 and VO_2, mixed in a 1:1 ratio of Na to V. Heating was performed at a rate of 10 °C/min up to 500 °C, while XRD scans were carried out once every 10 °C. (**b**) The weight fractions of all phases detected from the XRD measurements are also plotted as a function of temperature.

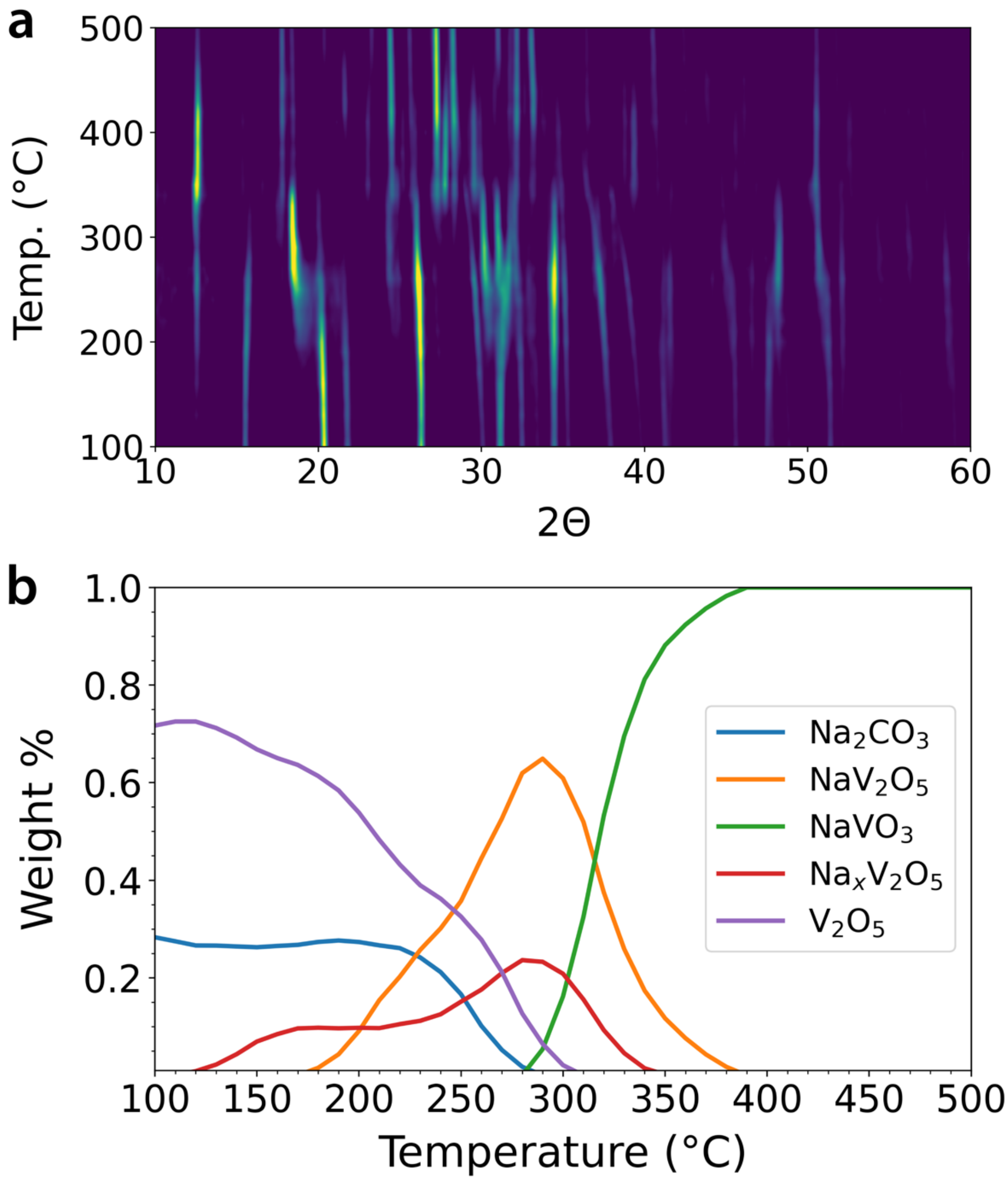

Supplementary Fig. 45 | *In-situ* characterization of the reaction sequence for Na_2CO_3 and V_2O_5. (**a**) The heatmap shows the temperature dependent XRD intensities measured from a sample of Na_2CO_3 and V_2O_5, mixed in a 1:1 ratio of Na to V. Heating was performed at a rate of 10 °C/min up to 500 °C, while XRD scans were carried out once every 10 °C. (**b**) The weight fractions of all phases detected from the XRD measurements are also plotted as a function of temperature.

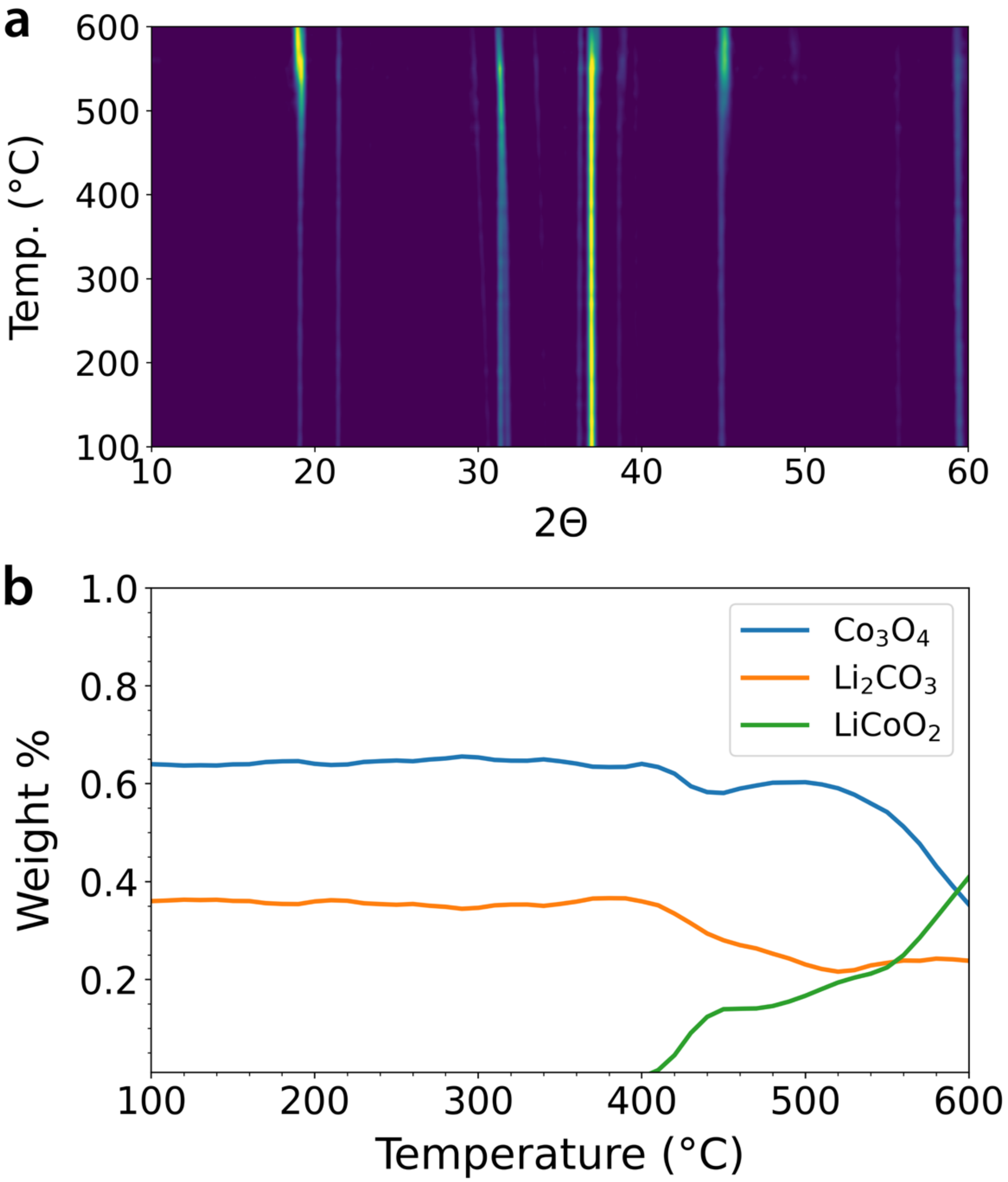

Supplementary Fig. 46 | *In-situ* characterization of the reaction sequence for Li_2CO_3 and Co_3O_4. (**a**) The heatmap shows the temperature dependent XRD intensities measured from a sample of Li_2CO_3 and Co_3O_4, mixed in a 1:1 ratio of Li to Co. Heating was performed at a rate of 10 °C/min up to 600 °C, while XRD scans were carried out once every 10 °C. (**b**) The weight fractions of all phases detected from the XRD measurements are also plotted as a function of temperature.

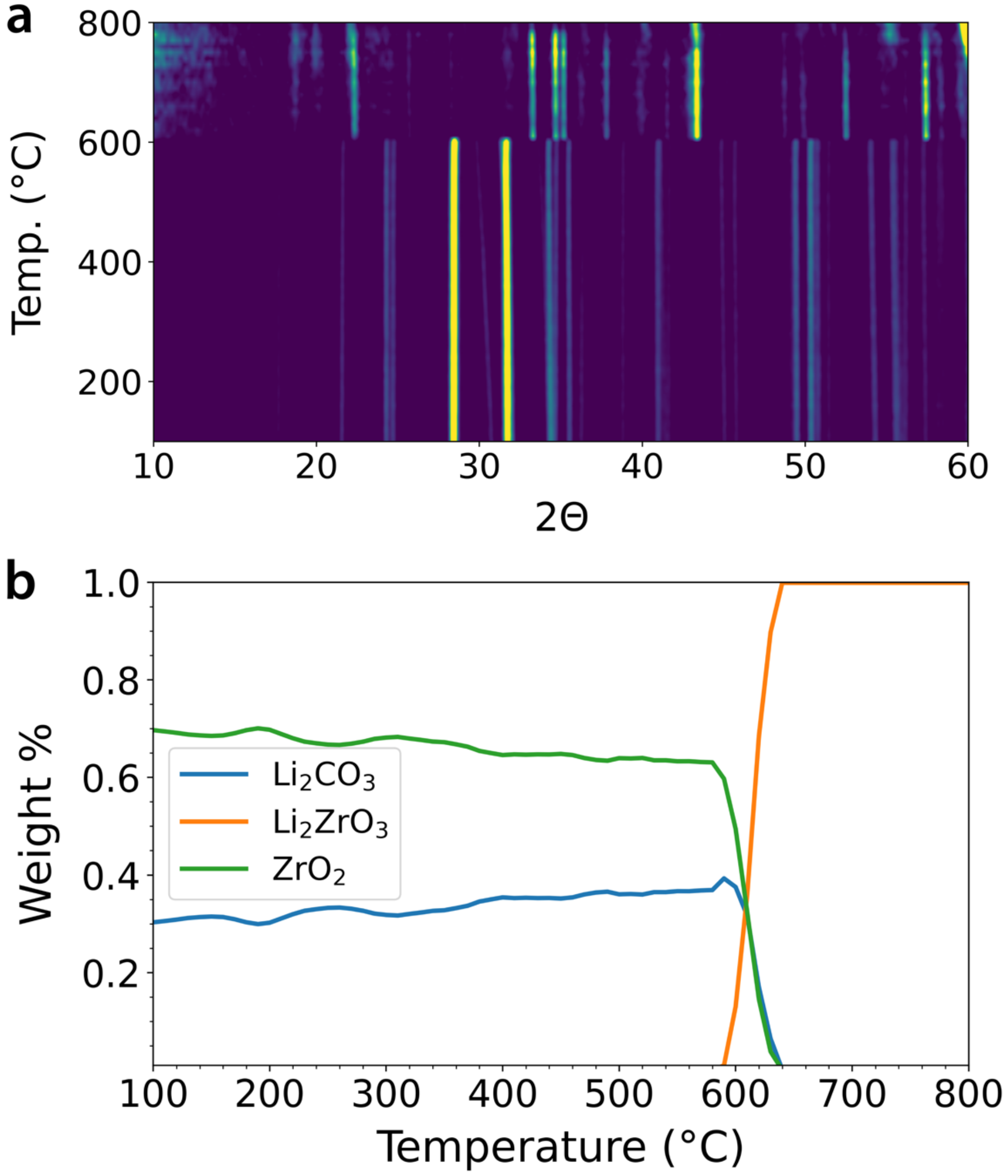

Supplementary Fig. 47 | *In-situ* characterization of the reaction sequence for Li_2CO_3 and ZrO_2. (**a**) The heatmap shows the temperature dependent XRD intensities measured from a sample of Li_2CO_3 and ZrO_2, mixed in a 1:1 ratio of Li to Zr. Heating was performed at a rate of 10 °C/min up to 800 °C, while XRD scans were carried out once every 10 °C. (**b**) The weight fractions of all phases detected from the XRD measurements are also plotted as a function of temperature.

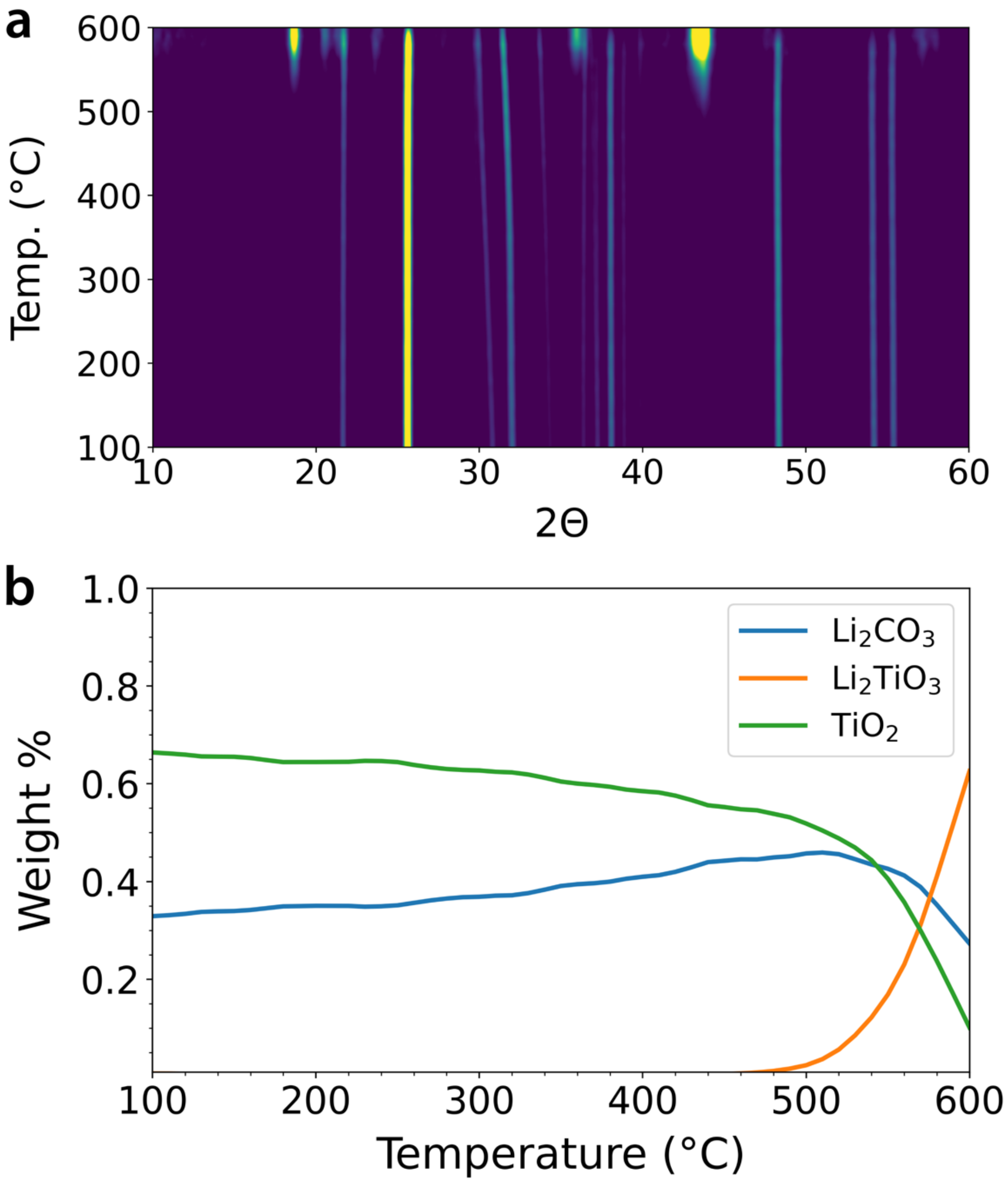

Supplementary Fig. 48 | *In-situ* characterization of the reaction sequence for Li_2CO_3 and TiO_2. (**a**) The heatmap shows the temperature dependent XRD intensities measured from a sample of Li_2CO_3 and TiO_2, mixed in a 1:1 ratio of Li to Ti. Heating was performed at a rate of 10 °C/min up to 600 °C, while XRD scans were carried out once every 10 °C. (**b**) The weight fractions of all phases detected from the XRD measurements are also plotted as a function of temperature.

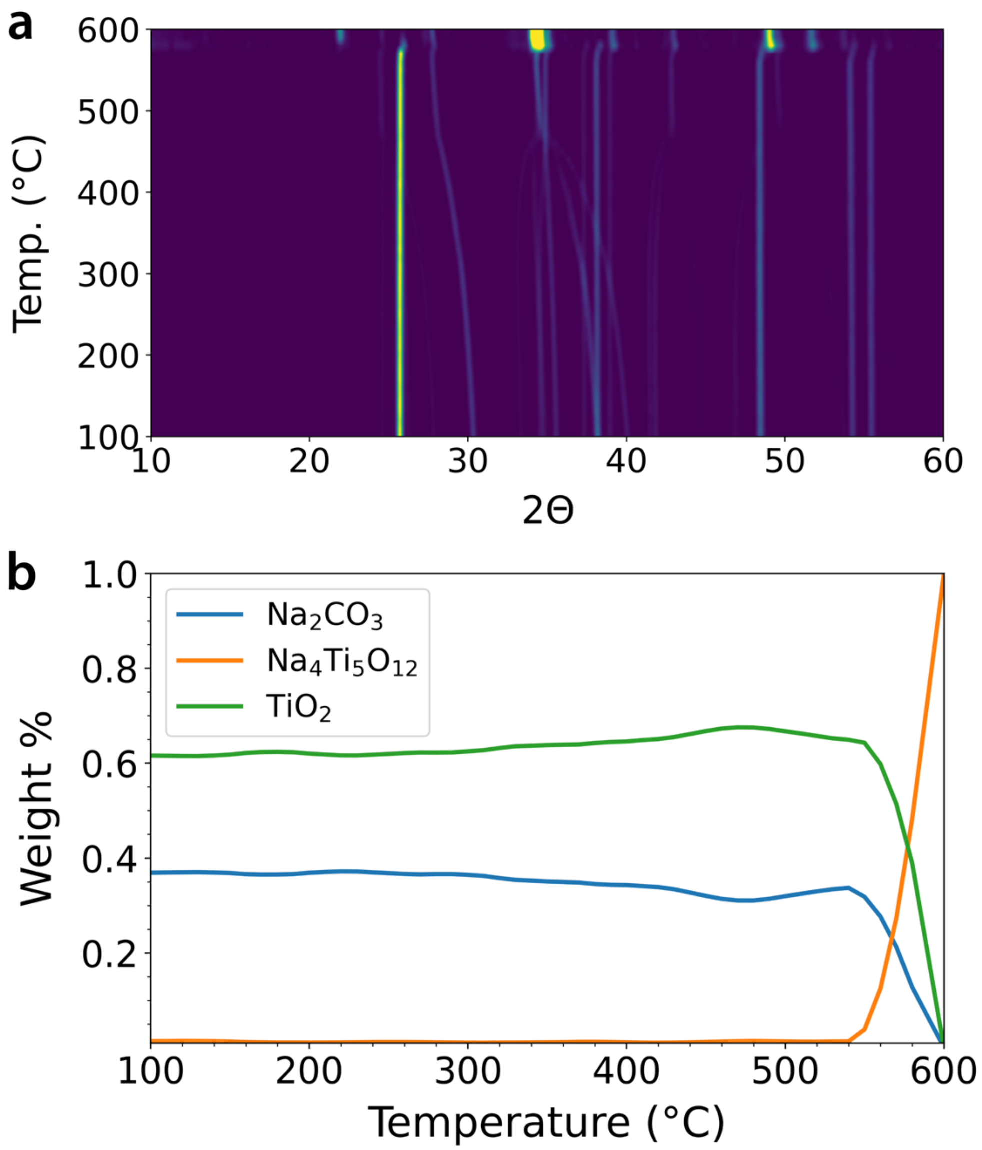

Supplementary Fig. 49 | *In-situ* characterization of the reaction sequence for Na_2CO_3 and TiO_2. (**a**) The heatmap shows the temperature dependent XRD intensities measured from a sample of Na_2CO_3 and TiO_2, mixed in a 1:1 ratio of Na to Ti. Heating was performed at a rate of 10 °C/min up to 600 °C, while XRD scans were carried out once every 10 °C. (**b**) The weight fractions of all phases detected from the XRD measurements are also plotted as a function of temperature.

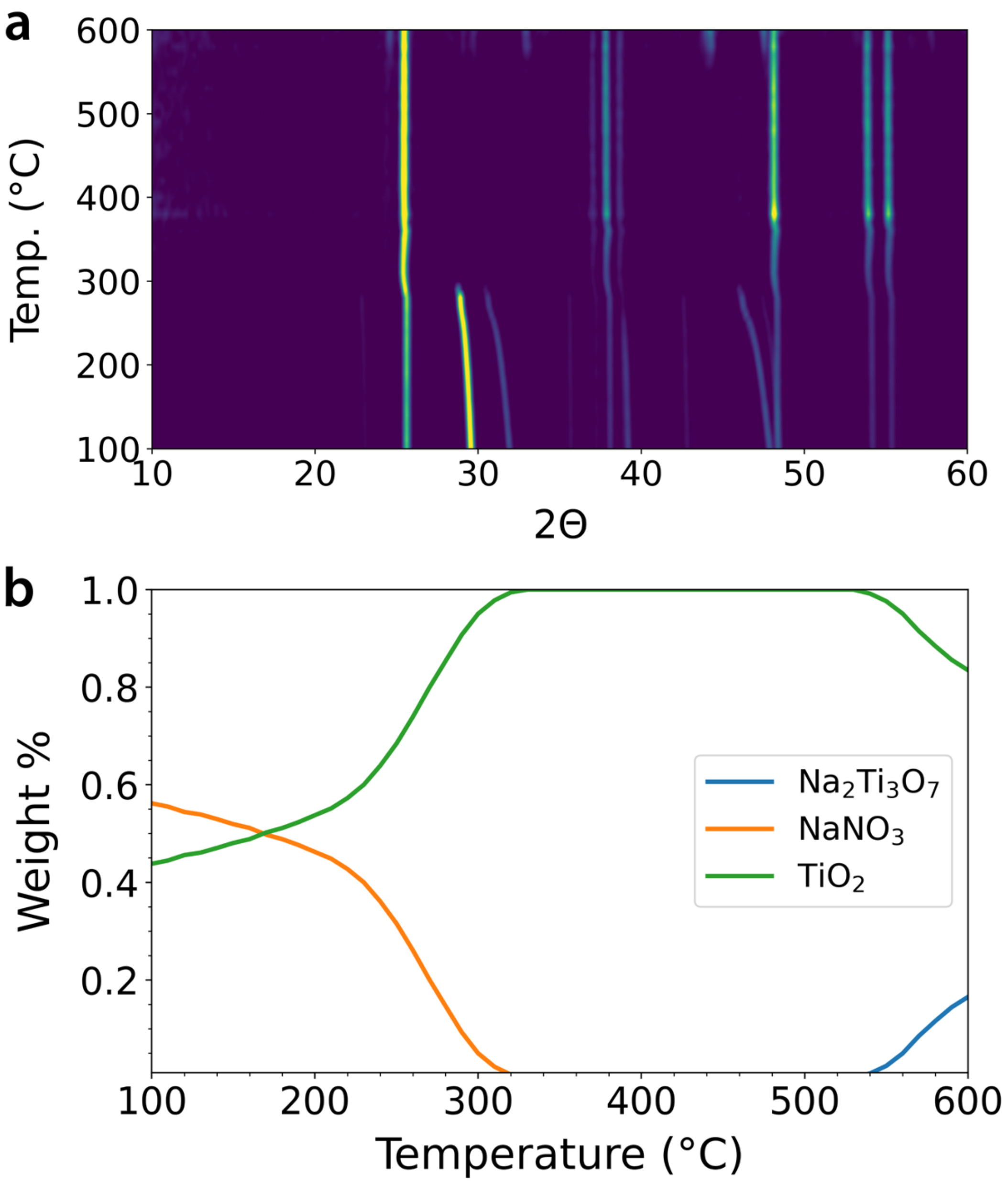

Supplementary Fig. 50 | *In-situ* characterization of the reaction sequence for $NaNO_3$ and TiO_2. (**a**) The heatmap shows the temperature dependent XRD intensities measured from a sample of $NaNO_3$ and TiO_2, mixed in a 1:1 ratio of Na to Ti. Heating was performed at a rate of 10 °C/min up to 600 °C, while XRD scans were carried out once every 10 °C. (**b**) The weight fractions of all phases detected from the XRD measurements are also plotted as a function of temperature.

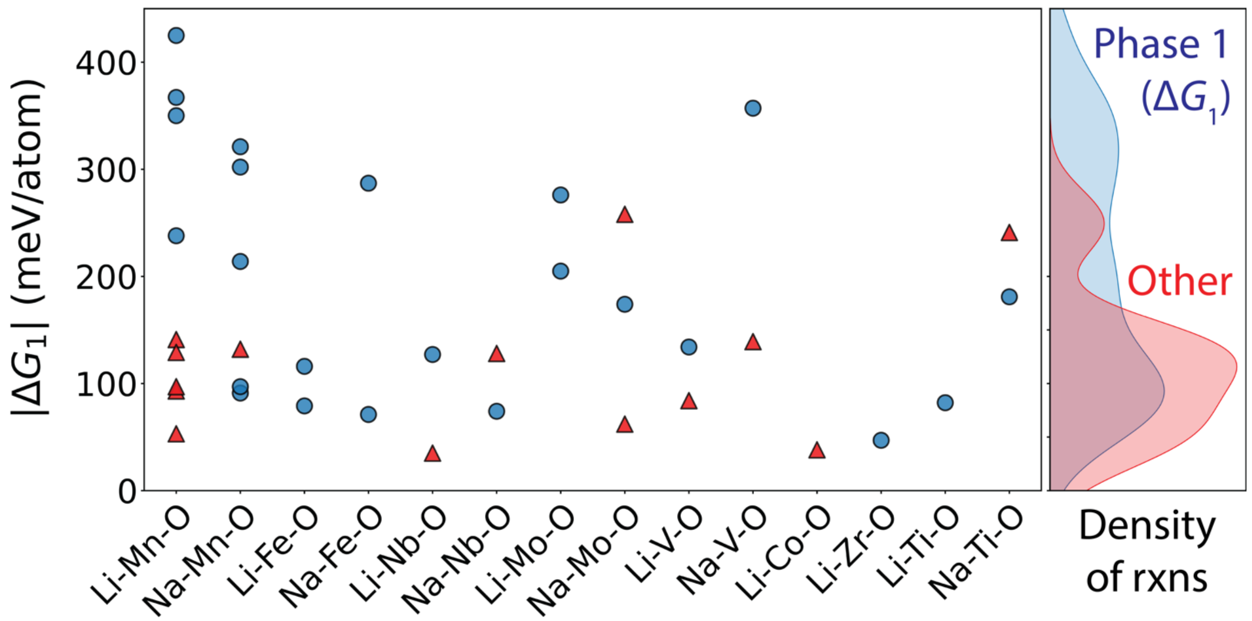

Supplementary Fig. 51 | Influence of the driving force magnitude on reaction outcomes. This plot is analogous to **Fig. 5** in the main text, except that all datapoints are now plotted with respect to the magnitude of the driving force associated with the most favorable product's formation (ΔG_1). Blue circles represent outcomes that obey max-ΔG theory while red triangles represent outcomes that do not. The right panel shows the kernel densities of these reactions. The results show that some degree of correlation exists between $|\Delta G_1|$ and the corresponding reaction outcomes. However, it is weaker than the correlation observed between $|\Delta G_1 - \Delta G_2|$ and the outcomes.

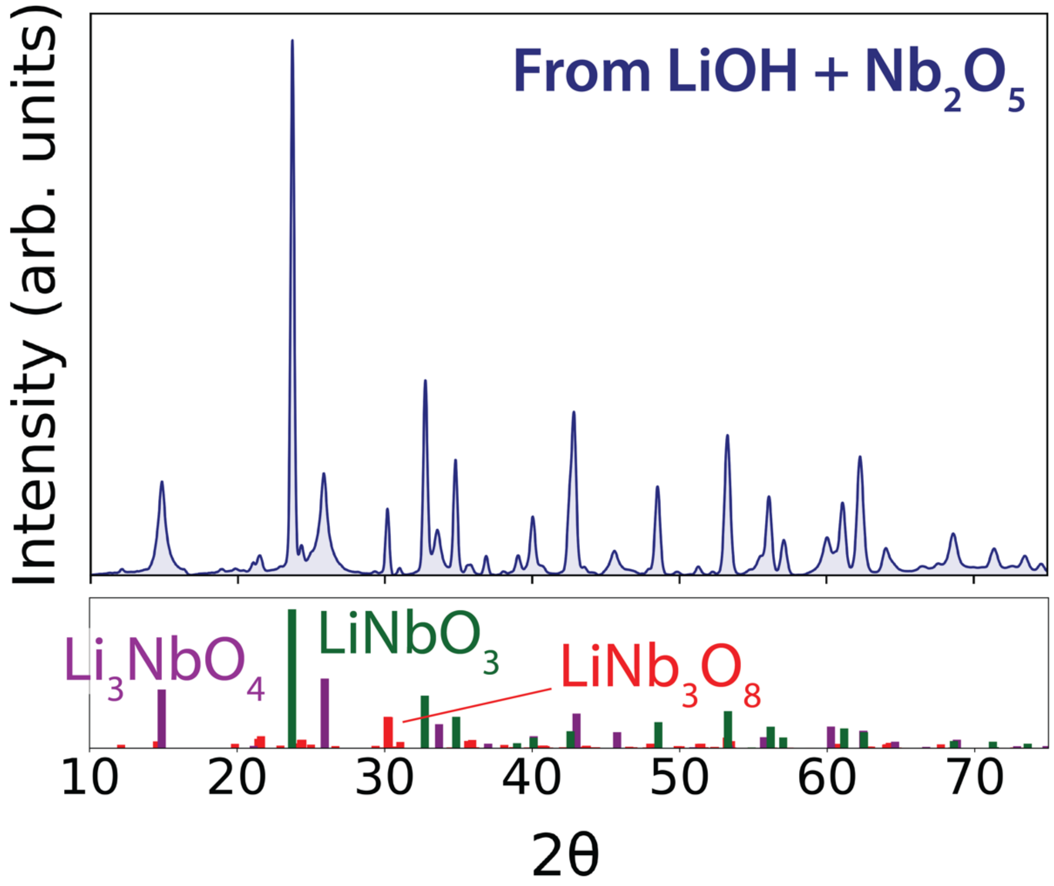

Supplementary Fig. 52 | Limited yield of $LiNbO_3$ obtained from LiOH and Nb_2O_5. The top panel shows the XRD pattern of the final sample produced by reactions between LiOH and Nb_2O_5. The bottom panel shows reference patterns for three phases detected in the XRD pattern: $LiNbO_3$, Li_3NbO_4, and $LiNb_3O_8$.

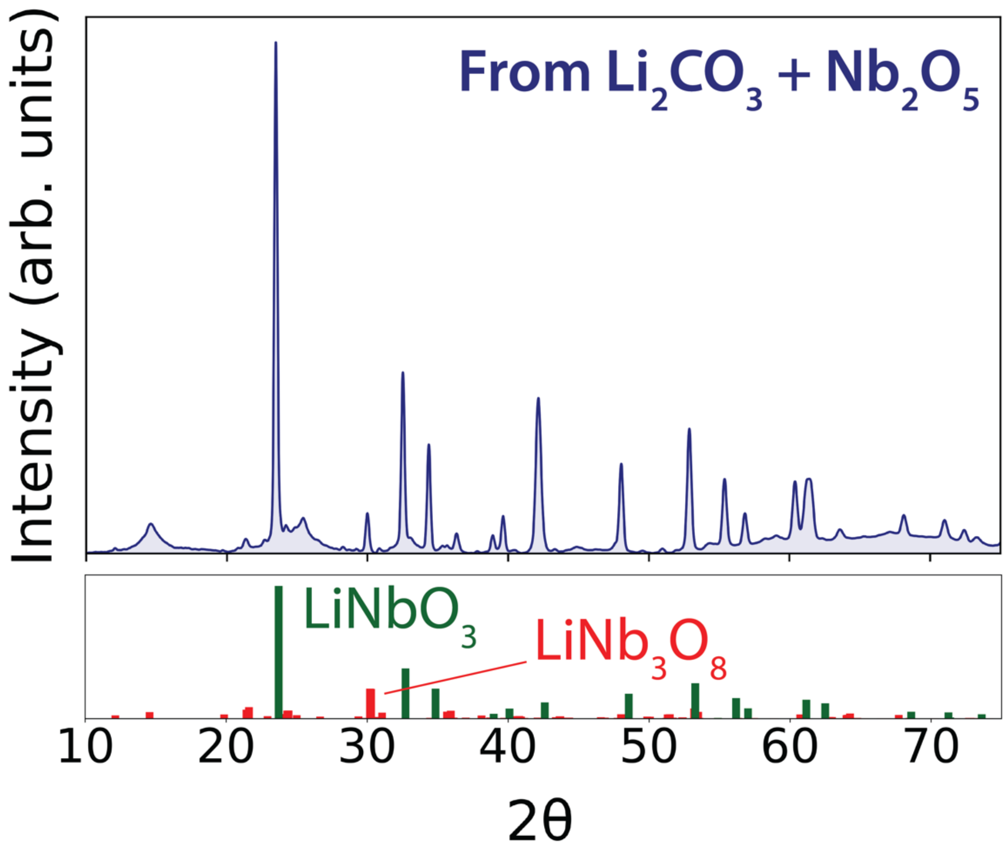

Supplementary Fig. 53 | High yield of $LiNbO_3$ obtained from Li_2CO_3 and Nb_2O_5. The top panel shows the XRD pattern of the final sample produced by reactions between Li_2CO_3 and Nb_2O_5. The bottom panel shows reference patterns for two phases detected in the XRD pattern: $LiNbO_3$ and $LiNb_3O_8$. There is also likely a small amount of Li_3NbO_4 impurity present in the sample.

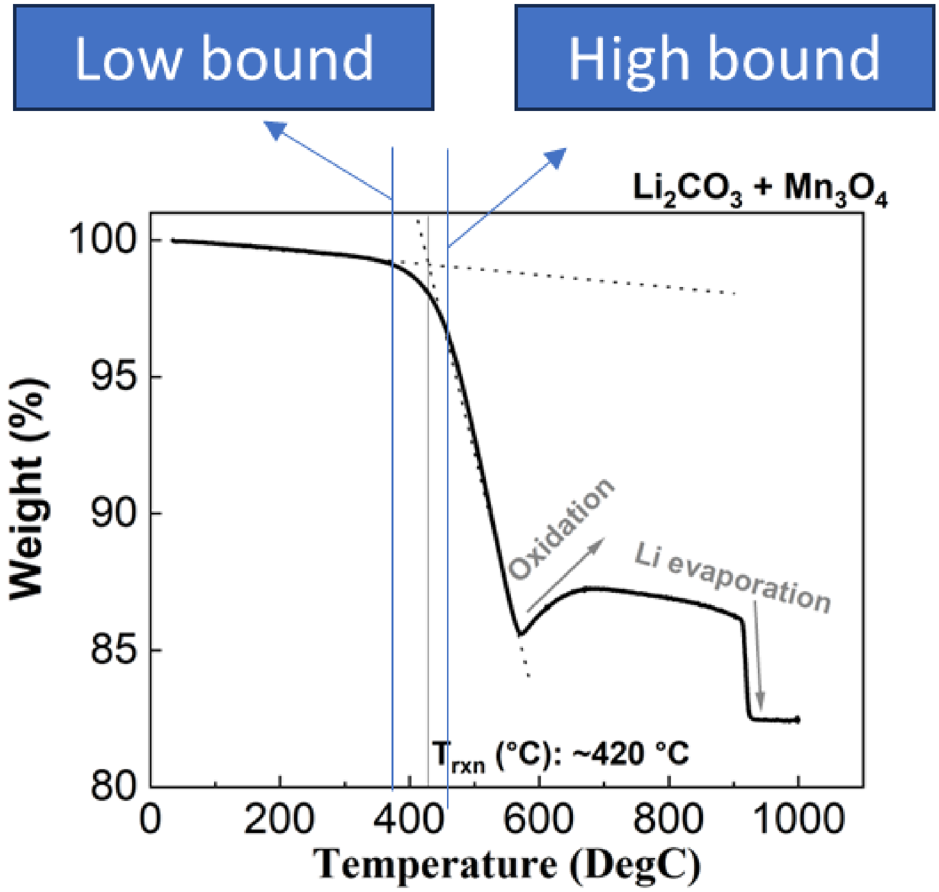

Supplementary Fig. 54 | Detection of reaction onset temperatures from TGA. The temperature at which a decomposition reaction begins to occur is determined by detecting weight loss in TGA. The lower bound on this temperature is equal to the point where the weight (%) curve first deviates from the linear fit of the low-temperature regime. Similarly, the upper bound is equal to the point where the weight (%) curve first reaches the linear fit of the higher-temperature regime.

Supplementary Table 1 | Reactants evaluated using in-house XRD. All pairwise combinations of the compounds below were heated to 600 °C and characterized using *in-situ* XRD. One pair of these compounds (Li_2CO_3 and ZrO_2) required a higher temperature of 800 °C to facilitate their reaction. Each combination included one alkali metal source and one transition metal source, mixed in a 1:1 ratio of the alkali to transition metal. We excluded any cases from our analysis in which either reactant decomposed or was oxidized before a ternary product could form. Also excluded were any samples that completely melted or became deformed prior to forming a ternary product. The final list of reactants included in our analysis is provided in Supplementary Table 2.

Element	Reactants tested
**Alkali metals**	
Li	Li_2CO_3, LiOH
Na	Na_2CO_3, $NaNO_3$
**Transition metals**	
Ti	TiO_2
V	VO_2, V_2O_5
Mn	MnO, Mn_3O_4, Mn_2O_3, MnO_2
Fe	Fe_3O_4, Fe_2O_3
Co	Co_3O_4
Zr	ZrO_2, $Zr(OH)_4$
Nb	Nb_2O_5
Mo	MoO_2, MoO_3

Supplementary Table 2 | Summary and analysis of pairwise reaction outcomes. For each pair of compounds included in our final analysis (Fig. 5 of the main text), we list the computed driving force (ΔG) to form each potential reaction product. These were calculated at the temperature where reactions were observed experimentally. In cases where there exist $>$ 3 possible reaction products, we only list those with the largest driving force to form. The driving force to form the most favorable product is bolded in each row. For comparison, we also list the initial product that formed experimentally in the right column.

Reactants	ΔG of each potential product (meV/atom)			Exp. outcome
	Li_3NbO_4	$LiNbO_3$	$LiNb_3O_8$	
Li_2CO_3, Nb_2O_5	**–35**	–30	–21	$LiNbO_3$
LiOH, Nb_2O_5	**–127**	–62	–37	Li_3NbO_4
	Li_2MnO_3	$LiMnO_2$	$LiMn_2O_4$	
Li_2CO_3, MnO	–224	–262	**–367**	$LiMn_2O_4$
Li_2CO_3, Mn_3O_4	**–93**	–47	–89	$LiMn_2O_4$
Li_2CO_3, Mn_2O_3	**–97**	–46	–37	$LiMn_2O_4$
Li_2CO_3, MnO_2	**–53**	–10	–39	$LiMn_2O_4$
LiOH, MnO	-255	–309	**–425**	$LiMn_2O_4$
LiOH, Mn_3O_4	**–141**	–108	–124	$LiMnO_2$
LiOH, Mn_2O_3	**–129**	–94	–98	$LiMnO_2$
LiOH, MnO_2	**–238**	–71	–141	Li_2MnO_3
Li_2O, MnO_2	**–250**	–67	–147	Li_2MnO_3
	$NaMnO_2$	$Na_2Mn_3O_7$		
Na_2CO_3, MnO	–278	**–321**		$Na_2Mn_3O_7$
Na_2CO_3, Mn_3O_4	–57	**–91**		$Na_2Mn_3O_7$
Na_2CO_3, MnO_2	–82	**–214**		$Na_2Mn_3O_7$
$NaNO_3$, MnO	–240	**–302**		$Na_2Mn_3O_7$
$NaNO_3$, Mn_2O_3	–86	**–97**		$Na_2Mn_3O_7$
$NaNO_3$, MnO_2	**–132**	–114		$Na_2Mn_3O_7$
	Li_5FeO_4	Li_2FeO_3	$LiFeO_2$	
Li_2CO_3, Fe_3O_4	38	–28	**–79**	$LiFeO_2$
LiOH, Fe_3O_4	92	–56	**–116**	$LiFeO_2$

	Na_3FeO_3	$NaFeO_2$		
Na_2CO_3, Fe_3O_4	42	**–71**		$NaFeO_2$
$NaNO_3$, Fe_3O_4	–156	**–287**		$NaFeO_2$
	Na_3NbO_4	$NaNbO_3$	$NaNb_3O_8$	
Na_2CO_3, Nb_2O_5	–7	**–74**	–24	$NaNbO_3$
$NaNO_3$, Nb_2O_5	–107	**–128**	–66	$Na_2Nb_4O_{11}$
	Li_4MoO_5	Li_2MoO_4		
Li_2CO_3, MoO_2	–81	**–205**		Li_2MoO_4
LiOH, MoO_2	–165	**–276**		Li_2MoO_4
	Na_4MoO_5	Na_2MoO_4	$Na_2Mo_2O_7$	
Na_2CO_3, MoO_2	–43	–211	**–258**	Na_2MoO_4
Na_2CO_3, MoO_3	32	–44	**–62**	Na_2MoO_4
$NaNO_3$, MoO_2	21	–72	**–174**	$Na_2Mo_2O_7$
	Li_3VO_4	$LiVO_3$	LiV_3O_8	
Li_2CO_3, VO_2	–112	**–134**	–132	$LiVO_3$
Li_2CO_3, V_2O_5	**–84**	–68	–31	LiV_2O_5
	Na_3VO_4	$NaVO_3$		
Na_2CO_3, VO_2	–153	**–357**		$NaVO_3$
Na_2CO_3, V_2O_5	–94	**–139**		$Na_xV_2O_5$ ($x < 1$)
	Li_6CoO_4	$LiCoO_2$	$LiCo_2O_4$	
Li_2CO_3, Co_3O_4	81	–32	**–38**	$LiCoO_2$
	$Li_6Zr_2O_7$	Li_2ZrO_3		
Li_2CO_3, ZrO_2	–37	**–47**		Li_2ZrO_3
	Li_4TiO_4	Li_2TiO_3	$Li_4Ti_5O_{12}$	
Li_2CO_3, TiO_2	–26	**–82**	–45	Li_2TiO_3
	$Na_8Ti_5O_{12}$	$Na_4Ti_5O_{12}$	$Na_2Ti_3O_7$	
Na_2CO_3, TiO_2	–139	**–181**	–63	$Na_4Ti_5O_{12}$
$NaNO_3$, TiO_2	–220	**–241**	–134	$Na_2Ti_3O_7$

Supplementary Table 3 | Enthalpy changes in the observed reactions. For each pair of compounds included in our final analysis (Fig. 5 of the main text), we list the computed enthalpy change (Δ*H*) associated with forming the observed product. Also provided is the entropy change (Δ*S*) of the reaction, and the temperature at which it occurs.

Reactants	Observed product	Δ*H* (meV/atom)	Reaction Temp. (°C)	Δ*S* (meV/atom/K)
Li_2CO_3, Nb_2O_5	$LiNbO_3$	54	500	0.167
LiOH, Nb_2O_5	Li_3NbO_4	-26	450	0.201
Li_2CO_3, MnO	$LiMn_2O_4$	-370	435	-0.007
Li_2CO_3, Mn_3O_4	$LiMn_2O_4$	-68	415	0.050
Li_2CO_3, Mn_2O_3	$LiMn_2O_4$	-54	425	0.078
Li_2CO_3, MnO_2	$LiMn_2O_4$	13	360	0.144
LiOH, MnO	$LiMn_2O_4$	-446	530	-0.039
LiOH, Mn_3O_4	$LiMnO_2$	-59	410	0.119
LiOH, Mn_2O_3	$LiMnO_2$	34	440	0.290
LiOH, MnO_2	Li_2MnO_3	-169	250	0.275
Li_2O, MnO_2	Li_2MnO_3	-250	230	0.000
Na_2CO_3, MnO	$Na_2Mn_3O_7$	-322	360	-0.004
Na_2CO_3, Mn_3O_4	$Na_2Mn_3O_7$	-77	340	0.040
Na_2CO_3, MnO_2	$Na_2Mn_3O_7$	-148	470	0.141
$NaNO_3$, MnO	$Na_2Mn_3O_7$	-214	380	0.232
$NaNO_3$, Mn_2O_3	$Na_2Mn_3O_7$	27	430	0.288
$NaNO_3$, MnO_2	$Na_2Mn_3O_7$	63	510	0.348
Li_2CO_3, Fe_3O_4	$LiFeO_2$	-42	400	0.110
LiOH, Fe_3O_4	$LiFeO_2$	-75	360	0.113
Na_2CO_3, Fe_3O_4	$NaFeO_2$	30	560	0.180
$NaNO_3$, Fe_3O_4	$NaFeO_2$	-41	550	0.447
Na_2CO_3, Nb_2O_5	$NaNbO_3$	30	310	0.141
$NaNO_3$, Nb_2O_5	$Na_2Nb_4O_{11}$	75	470	0.266
Li_2CO_3, MoO_2	Li_2MoO_4	-170	290	0.119
LiOH, MoO_2	Li_2MoO_4	-255	290	0.073

Na_2CO_3, MoO_2	Na_2MoO_4	-199	130	0.091
Na_2CO_3, MoO_3	Na_2MoO_4	-13	200	0.153
$NaNO_3$, MoO_2	$Na_2Mo_2O_7$	-123	240	0.211
Li_2CO_3, VO_2	$LiVO_3$	-123	150	0.074
Li_2CO_3, V_2O_5	LiV_2O_5	5	230	0.097
Na_2CO_3, VO_2	$NaVO_3$	-340	210	0.082
Na_2CO_3, V_2O_5	$Na_xV_2O_5$ ($x < 1$)	-71	120	0.071
Li_2CO_3, Co_3O_4	$LiCoO_2$	35	410	0.163
Li_2CO_3, ZrO_2	Li_2ZrO_3	106	600	0.255
Li_2CO_3, TiO_2	Li_2TiO_3	32	480	0.237
Na_2CO_3, TiO_2	$Na_4Ti_5O_{12}$	-90	550	0.165
$NaNO_3$, TiO_2	$Na_2Ti_3O_7$	10	550	0.454

Supplementary Data S1. Weight fractions obtained from *in-situ* X-ray diffraction.

The excel spreadsheet provided with the manuscript contains the weight fraction (%) of each phase identified from *in-situ* X-ray diffraction measurements. Each sheet corresponds to the results from a unique reactant pair. The first column contains temperature values, and the remaining columns contain weight fractions.

Supplementary Data S2. Rietveld refinements plots in the Li-Mn-O and Li-Nb-O spaces.

All Rietveld refinements included in this document are also attached as separate, high-resolution PDF files uploaded with the manuscript. The filename and title of each plots denote which reactant pair it was obtained from.
