# Supplementary material for: Quantifying the regime of thermodynamic control for solid-state reactions during ternary metal oxide synthesis
Source: Sci Adv. 2024 Jul 3;10(27):eadp3309. doi: 10.1126/sciadv.adp3309 (PMC11221506; doi:10.1126/sciadv.adp3309)
Supplement: Supplementary file 2 — Data S1 and S2 [file sciadv.adp3309_data_s1_and_s2.zip › adp3309_data_s2.zip!/Rietveld-Refinements/Li2O-MnO2.pdf]

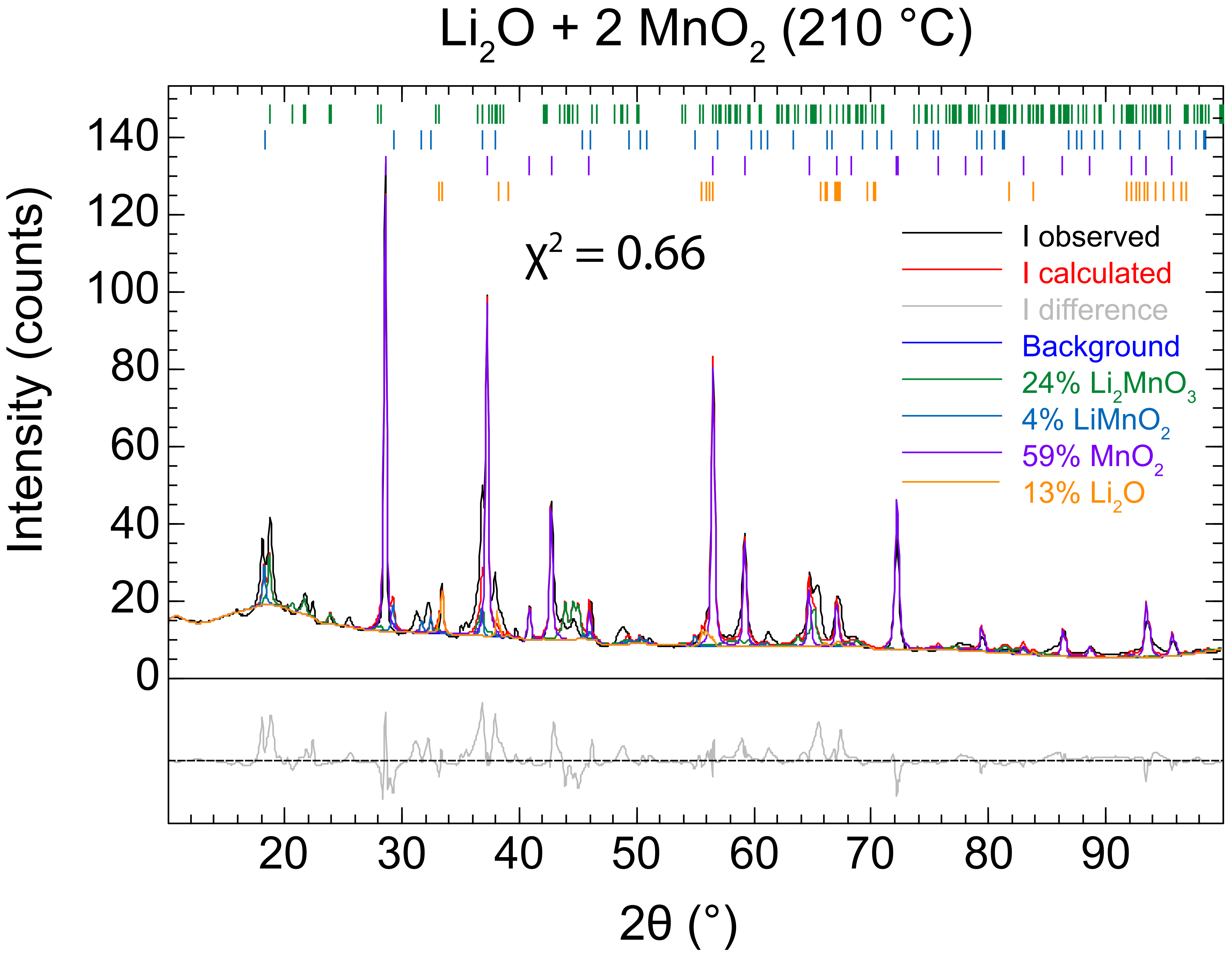

Li2O + 2 MnO2 (210 °C)
χ2 = 0.66
I observed
I calculated
I difference
Background
24% Li2MnO3
4% LiMnO2
59% MnO2
13% Li2O
Intensity (counts)
2θ (°)
0
20
40
60
80
100
120
140
20
30
40
50
60
70
80
90
