# Supplementary material for: Quantifying the regime of thermodynamic control for solid-state reactions during ternary metal oxide synthesis
Source: Sci Adv. 2024 Jul 3;10(27):eadp3309. doi: 10.1126/sciadv.adp3309 (PMC11221506; doi:10.1126/sciadv.adp3309)
Supplement: Supplementary file 2 — Data S1 and S2 [file sciadv.adp3309_data_s1_and_s2.zip › adp3309_data_s2.zip!/Rietveld-Refinements/Li2CO3-MnO.pdf]

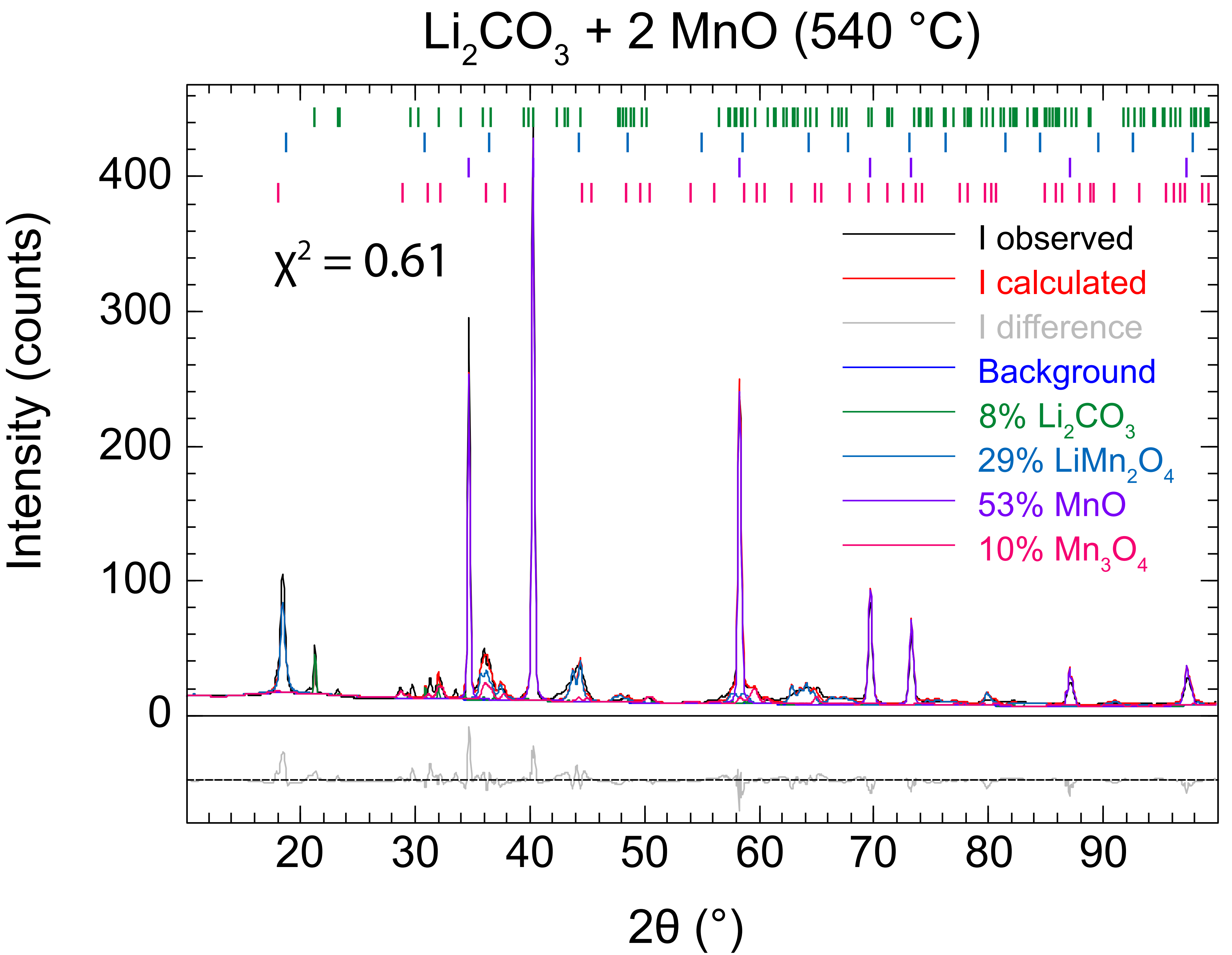

$Li_2CO_3 + 2\ MnO$ (540 °C)
$\chi^2 = 0.61$
I observed
I calculated
I difference
Background
8% Li_2CO_3
29% $LiMn_2O_4$
53% MnO
10% Mn_3O_4
Intensity (counts)
2θ (°)
0
100
200
300
400
20
30
40
50
60
70
80
90
