# Supplementary material for: Quantifying the regime of thermodynamic control for solid-state reactions during ternary metal oxide synthesis
Source: Sci Adv. 2024 Jul 3;10(27):eadp3309. doi: 10.1126/sciadv.adp3309 (PMC11221506; doi:10.1126/sciadv.adp3309)
Supplement: Supplementary file 2 — Data S1 and S2 [file sciadv.adp3309_data_s1_and_s2.zip › adp3309_data_s2.zip!/Rietveld-Refinements/LiOH-Mn2O3.pdf]

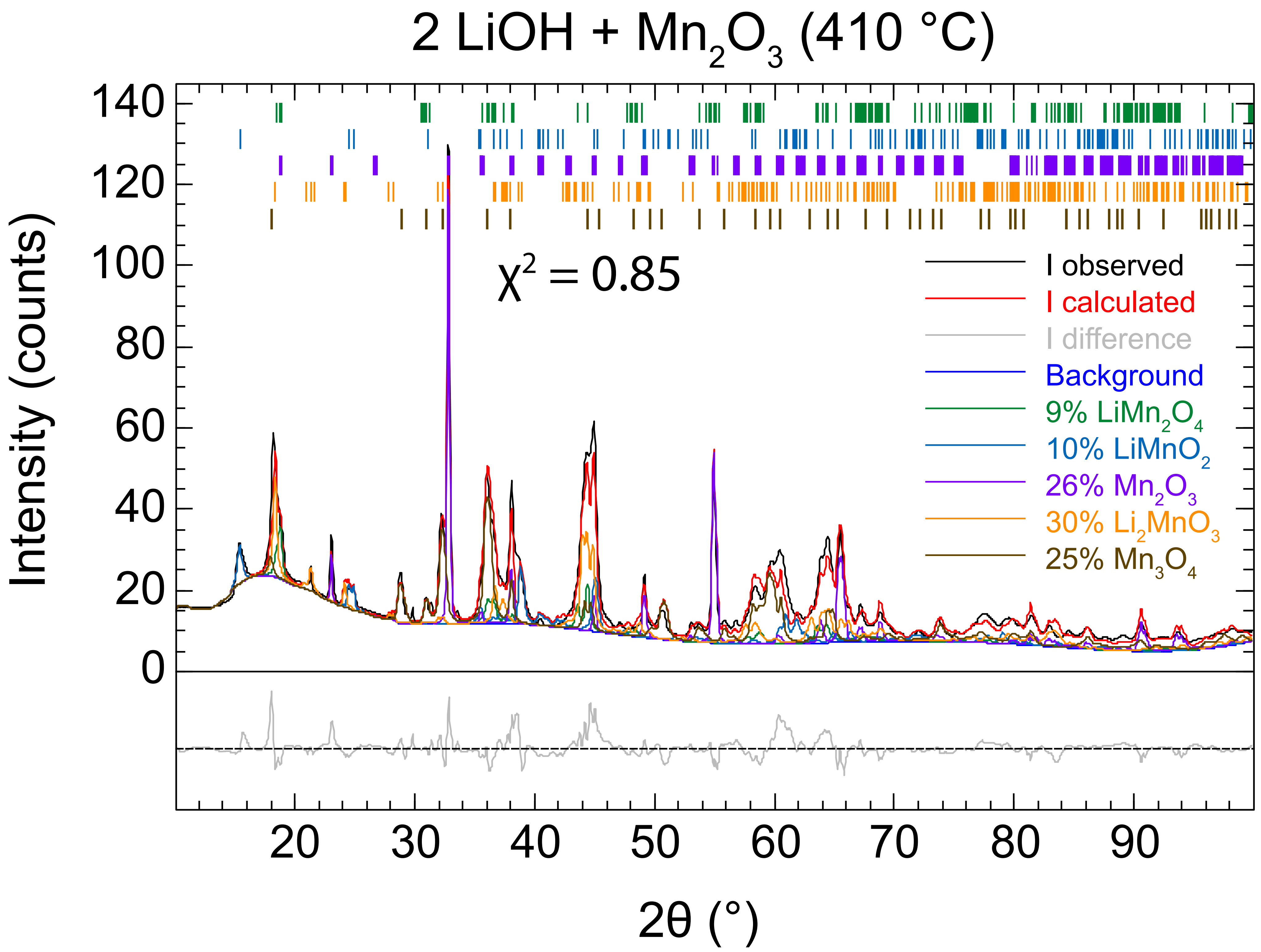

2 LiOH + Mn2O3 (410 °C)
Intensity (counts)
2θ (°)
χ2 = 0.85
I observed
I calculated
I difference
Background
9% LiMn2O4
10% LiMnO2
26% Mn2O3
30% Li2MnO3
25% Mn3O4
0
20
40
60
80
100
120
140
20
30
40
50
60
70
80
90
