# Supplementary material for: Quantifying the regime of thermodynamic control for solid-state reactions during ternary metal oxide synthesis
Source: Sci Adv. 2024 Jul 3;10(27):eadp3309. doi: 10.1126/sciadv.adp3309 (PMC11221506; doi:10.1126/sciadv.adp3309)
Supplement: Supplementary file 2 — Data S1 and S2 [file sciadv.adp3309_data_s1_and_s2.zip › adp3309_data_s2.zip!/Rietveld-Refinements/Li2CO3-Mn3O4.pdf]

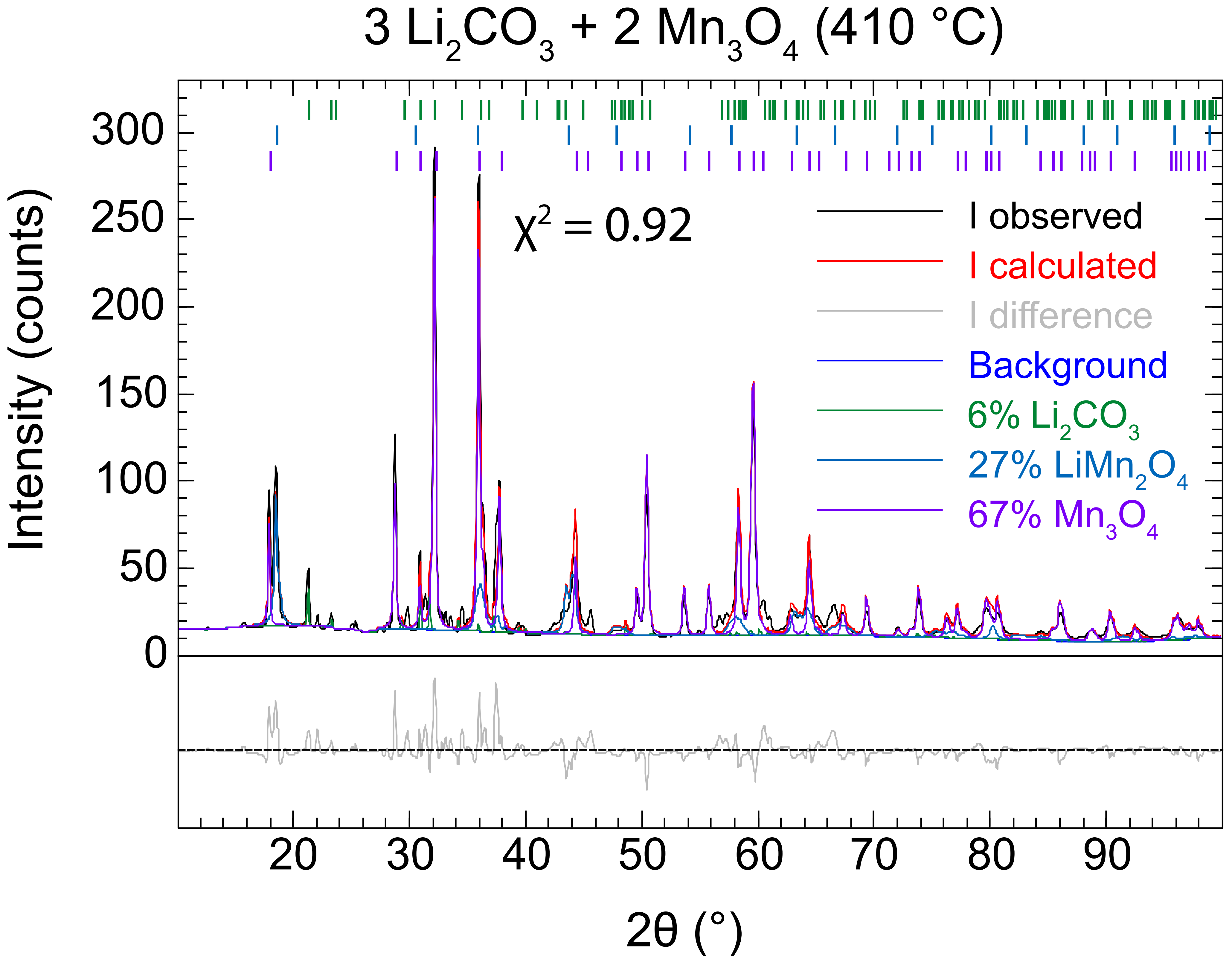
3 Li2CO3 + 2 Mn3O4 (410 °C)
χ2 = 0.92
I observed
I calculated
I difference
Background
6% Li2CO3
27% LiMn2O4
67% Mn3O4
Intensity (counts)
2θ (°)
0
50
100
150
200
250
300
20
30
40
50
60
70
80
90
