# Supplementary material for: Quantifying the regime of thermodynamic control for solid-state reactions during ternary metal oxide synthesis
Source: Sci Adv. 2024 Jul 3;10(27):eadp3309. doi: 10.1126/sciadv.adp3309 (PMC11221506; doi:10.1126/sciadv.adp3309)
Supplement: Supplementary file 2 — Data S1 and S2 [file sciadv.adp3309_data_s1_and_s2.zip › adp3309_data_s2.zip!/Rietveld-Refinements/LiOH-Nb2O5.pdf]

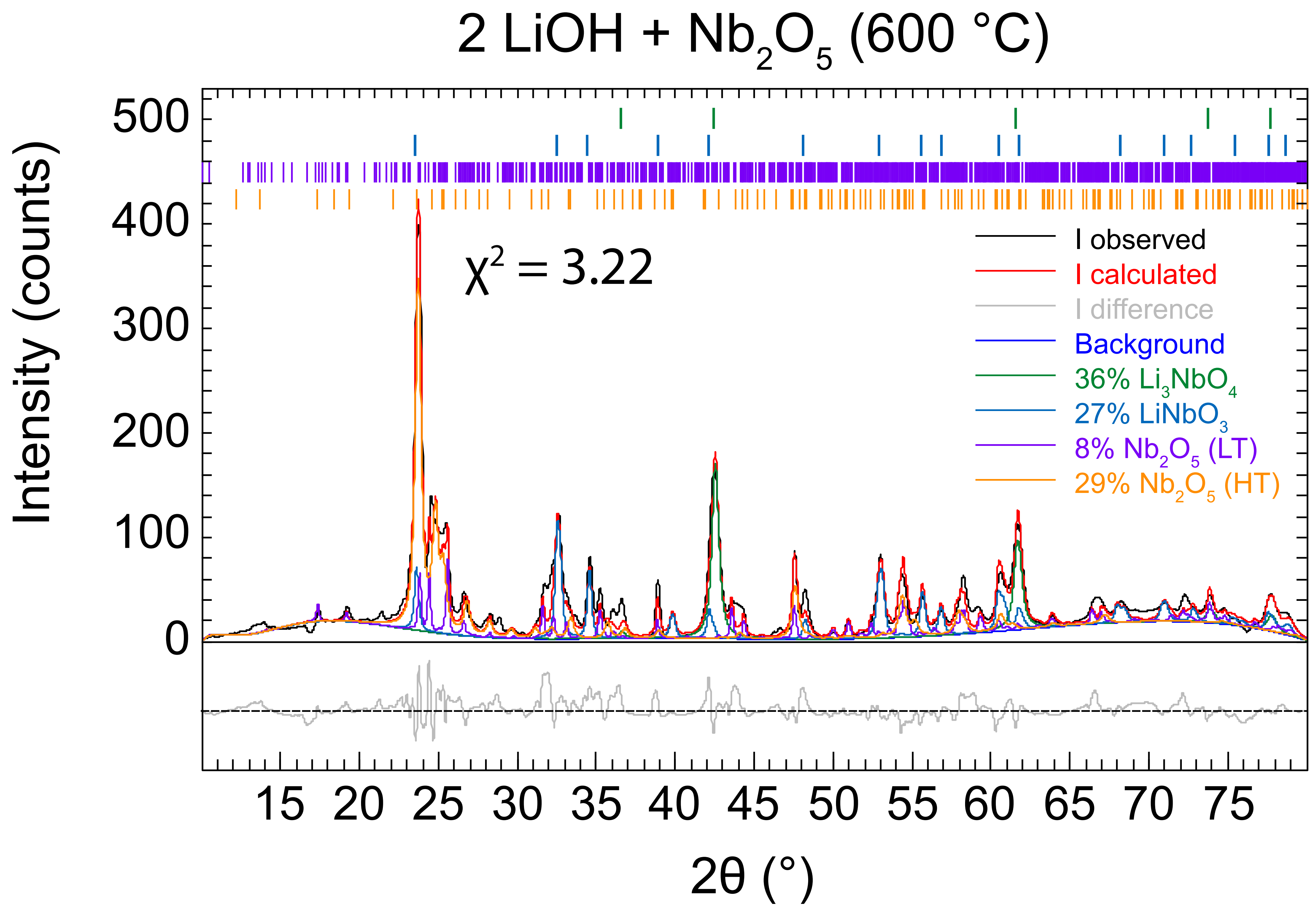

2 LiOH + Nb2O5 (600 °C)
Intensity (counts)
2θ (°)
χ2 = 3.22
I observed
I calculated
I difference
Background
36% Li3NbO4
27% LiNbO3
8% Nb2O5 (LT)
29% Nb2O5 (HT)
0
100
200
300
400
500
15
20
25
30
35
40
45
50
55
60
65
70
75
