# Supplementary material for: Quantifying the regime of thermodynamic control for solid-state reactions during ternary metal oxide synthesis
Source: Sci Adv. 2024 Jul 3;10(27):eadp3309. doi: 10.1126/sciadv.adp3309 (PMC11221506; doi:10.1126/sciadv.adp3309)
Supplement: Supplementary file 2 — Data S1 and S2 [file sciadv.adp3309_data_s1_and_s2.zip › adp3309_data_s2.zip!/Rietveld-Refinements/Li2CO3-Nb2O5.pdf]

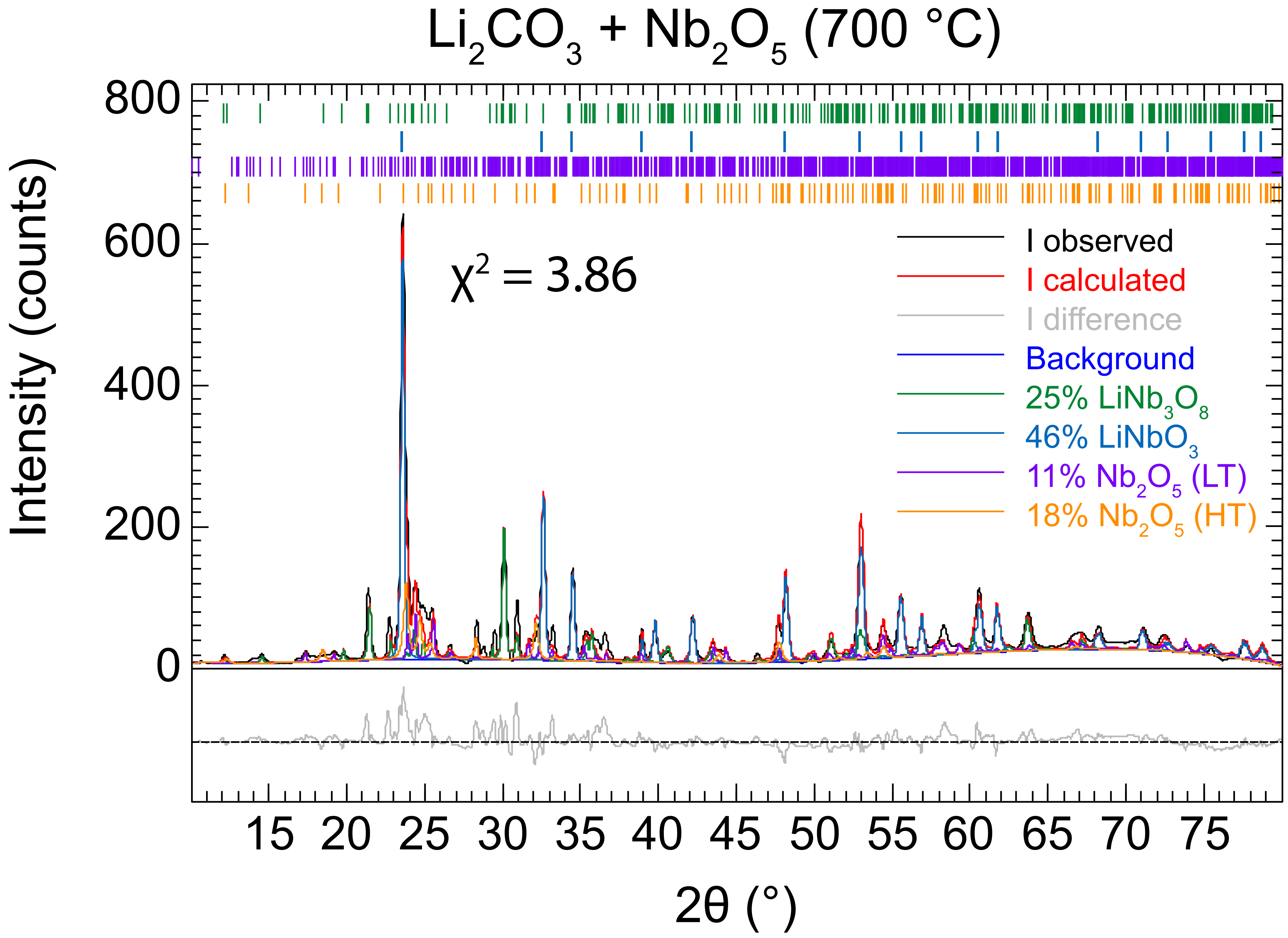

Li2CO3 + Nb2O5 (700 °C)
Intensity (counts)
800
600
400
200
0
χ2 = 3.86
I observed
I calculated
I difference
Background
25% LiNb3O8
46% LiNbO3
11% Nb2O5 (LT)
18% Nb2O5 (HT)
15
20
25
30
35
40
45
50
55
60
65
70
75
2θ (°)
