# Supplementary material for: Quantifying the regime of thermodynamic control for solid-state reactions during ternary metal oxide synthesis
Source: Sci Adv. 2024 Jul 3;10(27):eadp3309. doi: 10.1126/sciadv.adp3309 (PMC11221506; doi:10.1126/sciadv.adp3309)
Supplement: Supplementary file 2 — Data S1 and S2 [file sciadv.adp3309_data_s1_and_s2.zip › adp3309_data_s2.zip!/Rietveld-Refinements/LiOH-Mn3O4.pdf]

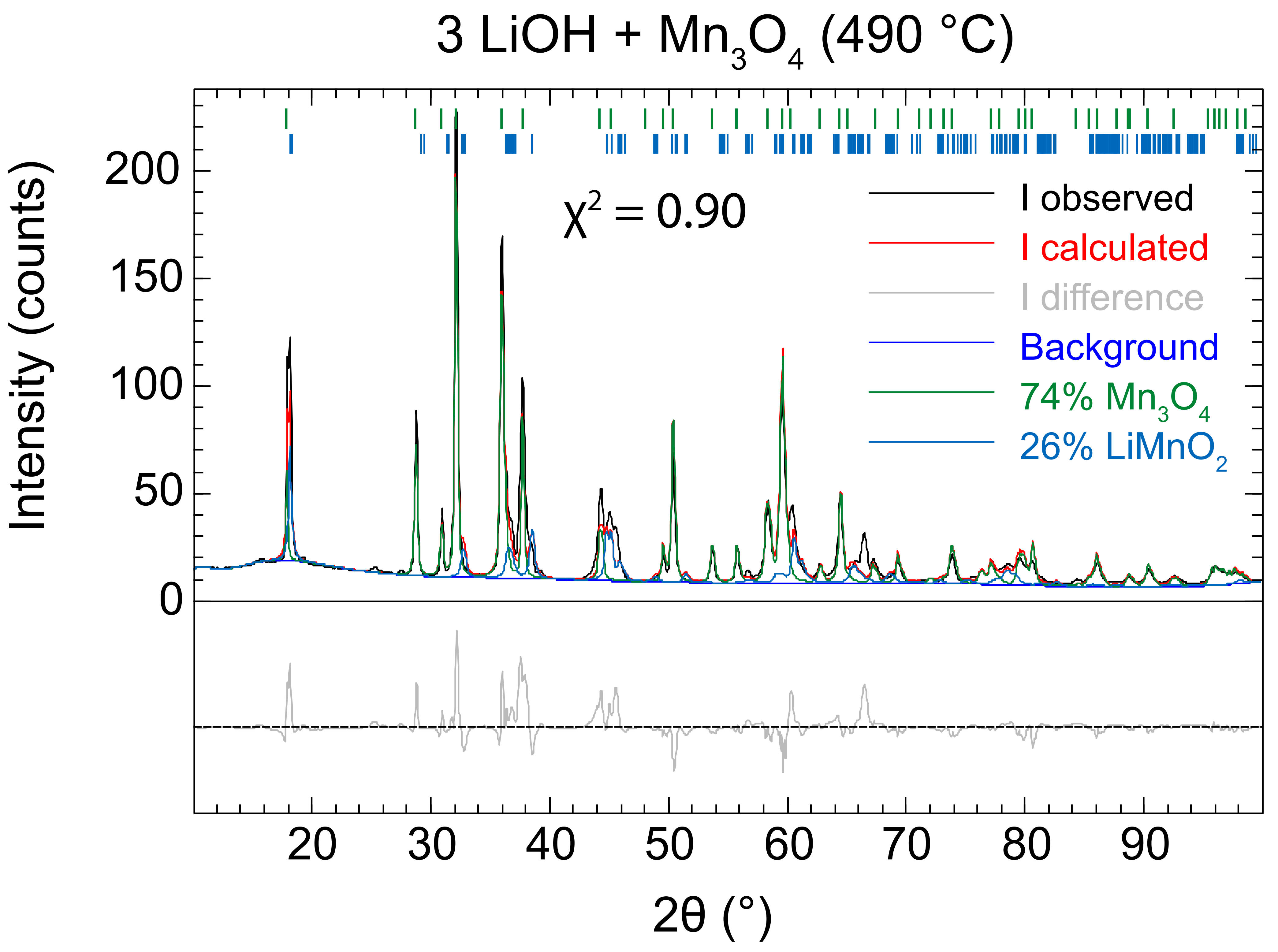

3 LiOH + Mn$_3$O$_4$ (490 °C)
χ^2 = 0.90
I observed
I calculated
I difference
Background
74% Mn$_3$O$_4$
26% LiMnO$_2$
Intensity (counts)
0
50
100
150
200
20
30
40
50
60
70
80
90
2θ (°)
